# Dispersal behaviour and riverine network connectivity shape the genetic diversity of freshwater amphipod metapopulations

**DOI:** 10.1111/mec.16201

**Published:** 2021-10-10

**Authors:** Roman Alther, Emanuel A. Fronhofer, Florian Altermatt

**Affiliations:** ^1^ Department of Aquatic Ecology Eawag, Swiss Federal Institute of Aquatic Science and Technology Dübendorf Switzerland; ^2^ Department of Evolutionary Biology and Environmental Studies University of Zurich Zürich Switzerland; ^3^ ISEM, CNRS, IRD, EPHE Université de Montpellier Montpellier France

**Keywords:** connectivity, *Gammarus fossarum*, landscape genetics, population genetics, stochastic simulation

## Abstract

Theory predicts that the distribution of genetic diversity in a landscape is strongly dependent on the connectivity of the metapopulation and the dispersal of individuals between patches. However, the influence of explicit spatial configurations such as dendritic landscapes on the genetic diversity of metapopulations is still understudied, and theoretical corroborations of empirical patterns are largely lacking. Here, we used microsatellite data and stochastic simulations of two metapopulations of freshwater amphipods in a 28,000 km^2^ riverine network to study the influence of spatial connectivity and dispersal strategies on the spatial distribution of their genetic diversity. We found a significant imprint of the effects of riverine network connectivity on the local and global genetic diversity of both amphipod species. Data from 95 sites showed that allelic richness significantly increased towards more central nodes of the network. This was also seen for observed heterozygosity, yet not for expected heterozygosity. Genetic differentiation increased with instream distance. In simulation models, depending on the mutational model assumed, upstream movement probability and dispersal rate, respectively, emerged as key factors explaining the empirically observed distribution of local genetic diversity and genetic differentiation. Surprisingly, the role of site‐specific carrying capacities, for example by assuming a direct dependency of population size on local river size, was less clear cut: while our best fitting model scenario included this feature, over all simulations, scaling of carrying capacities did not increase data‐model fit. This highlights the importance of dispersal behaviour along spatial networks in shaping population genetic diversity.

## 1 INTRODUCTION

The genetic diversity of populations is shaped by gene flow, selection, mutation, and genetic drift (Hartl & Clark, 2006; Manel et al., 2003). These processes interact with ecological processes, determining the organisms’ demography, population size and dynamics. Understanding both ecological and evolutionary processes affecting natural populations is thus central to the understanding of patterns and dynamics of biological diversity and for implementing appropriate conservation strategies (Balkenhol et al., 2016; Lande, 1988), especially in the context of habitat fragmentation.

Extensive theoretical and empirical work highlights that dispersal has a pronounced effect on the genetic diversity and effective size of populations (Bowler & Benton, 2005; Clobert et al., 2012) and community composition (Vellend, 2016). Dispersal is defined as the movement of organisms with potential consequences for gene flow (Ronce, 2007), and is especially relevant in spatially structured landscapes (Gilpin & Hanski, 1991; Hanski & Simberloff, 1997). Of course, the effects of dispersal can be modulated by features of landscape connectivity or spatial distributions of patch sizes, for example (Hanski & Gaggiotti, 2004). These need to be considered to understand the overall effects of dispersal on the genetic diversity of natural populations. Understanding the importance of different aspects of dispersal for natural populations is empirically challenging because both the processes influencing individual dispersal and its population genetic consequences need to be explored simultaneously.

The study of dispersal has a long tradition in both landscape ecology and metapopulation ecology, respectively, using slightly different tools and perspectives (Clobert et al., 2012; Hanski & Gaggiotti, 2004; Leibold et al., 2004; Vellend, 2016). Ideally, approaches combine measures of genetic diversity and landscape connectivity, thereby linking physical connectivity to population genetics (Manel & Holderegger, 2013). The metapopulation approach provides the means of including connectivity more explicitly (Hanski & Gaggiotti, 2004). Studies about the influence of landscape connectivity on the genetic diversity of populations mostly study lattice‐like landscapes (2D), such as grasslands or forests (Dyer et al., 2012; Fortuna et al., 2009; Rozenfeld et al., 2008), or n‐island models (Wright, 1931), using least‐cost path methods with landscape resistance to integrate their spatial complexity (Adriaensen et al., 2003; Pinto & Keitt, 2009; Wang et al., 2009). However, this may not be generalized to the spatial structure of all ecosystems, and dispersal of organisms may be more strongly confined in spatially more complex ecosystems.

Riverine systems are a prominent example thereof. Their connectivity is highly characteristic, can be explicitly quantified, and generally follows a universal dendritic network structure. These systems are formed by geological processes leading to general topological patterns (Altermatt, 2013; Carraro et al., 2020; Rodríguez‐Iturbe & Rinaldo, 1997). Ecological consequences of the spatial configuration in such networks are well‐studied, and encompass effects on species richness, on beta‐diversity, as well as on population sizes (Altermatt et al., 2013; Carrara et al., 2012; Henriques‐Silva et al., 2019; Muneepeerakul et al., 2008; Tonkin et al., 2018). In contrast, the evolutionary consequences of the network structure on the intraspecific genetic diversity are less well understood, even though one could apply the same approaches to study them. Comparative studies focusing on the effect of riverine network structures on intraspecific genetic diversity within populations are still rare (Blanchet et al., 2020; Brauer et al., 2018; Fourtune et al., 2016), but generally show an increase in diversity in more downstream parts of the network (“downstream increase in intraspecific genetic diversity” (DIGD; Paz‐Vinas et al., 2015) or in highly‐connected sections such as confluences (Paz‐Vinas & Blanchet, 2015). Importantly, these studies highlight that various processes, such as dendritic connectivity (i.e., habitat fragmentation), asymmetric gene flow, or remnant signals of past colonization histories can lead to the empirically observed patterns (Blanchet et al., 2020; Cyr & Angers, 2011; Paz‐Vinas et al., 2015). In parallel, theoretical models that address the effect of spatial connectivity of riverine networks on genetic variation (Morrissey & de Kerckhove, 2009; Paz‐Vinas & Blanchet, 2015; Paz‐Vinas et al., 2015), on evolution of dispersal (Henriques‐Silva et al., 2015), and emergence of neutral genetic structure (Fronhofer & Altermatt, 2017; Stokes & Perron, 2020; Thomaz et al., 2016) have demonstrated that dispersal along riverine networks has a direct imprint on the genetic structure and diversity of the inhabiting organisms. While these theoretical models provide direct testable predictions, a direct comparison between within‐population genetic diversities estimated from empirical data and predictions from theoretical models assuming an identical riverine network has been largely lacking (but see Chiu, Li, et al., 2020; Chiu, Nukazawa, et al., 2020).

Here, we studied the influence of connectivity in a real‐world riverine network on within‐ and between‐populations genetic diversity of freshwater amphipods (crustaceans), by combining large‐scale empirical data on their population genetic diversity with a simulation model making analogue predictions of their population genetic diversity using a graph theoretic approach. Graph theory has not yet been widely used in landscape genetics although it allows concise presentation of spatial configuration of natural populations (Dyer & Nason, 2004; Fortuna et al., 2009; Garroway et al., 2008; Manel & Holderegger, 2013; but see McRae et al., 2008; McRae & Beier, 2007). Based on previous work (Altermatt & Fronhofer, 2018; Blanchet et al., 2020; Fronhofer & Altermatt, 2017; Muneepeerakul et al., 2007), we wanted to gain a better understanding of the relative importance of different ecologically relevant aspects of dispersal on shaping the genetic diversity of populations. In particular, we expected allelic richness, observed, and expected heterozygosity to be higher in more central sections (i.e., downstream or confluences) of the riverine network (Paz‐Vinas & Blanchet, 2015; Ritland, 1989). This increase in genetic diversity in central sections of the network might be caused by a strong signal of dispersal rate, upstream movement probability, and habitat carrying capacity, leading to those sections receiving more migrants and sustain larger populations (centrality aspect; Altermatt, 2013). We addressed this with microsatellite data from 3319 amphipod individuals collected from 95 sites across a riverine network covering more than 28,000 km^2^ and compared it to the output of stochastic simulation models examining alternative parameter combinations influencing dispersal, but conducted on the identical riverine network structure.

## 2 MATERIALS AND METHODS

### 2.1 Study system

*Gammarus fossarum* (Koch) is a common and wide‐spread freshwater amphipod species complex (Crustacea, Amphipoda), predominantly found in smaller streams and distributed throughout Central Europe (Karaman & Pinkster, 1977; Wattier et al., 2020; Weiss et al., 2014) and adjacent biogeographic regions. As a major decomposer, it has an important role in aquatic food webs (Hieber & Gessner, 2002; Little & Altermatt, 2018). The species complex contains a high cryptic diversity, with several to dozens of species being reported, but not yet formally described (Müller, 2000; Wattier et al., 2020; Weiss et al., 2014). In Switzerland, two of those cryptic lineages are widely distributed (Altermatt et al., 2014, 2019; Westram et al., 2011, 2013). These lineages are reproductively isolated, and are considered valid species that diverged ~15 Ma years ago (Wattier et al., 2020), herein referred to as *Gammarus fossarum* type A (*G*. *fossarum* A) and *Gammarus fossarum* type B (*G*. *fossarum* B, both sensu Müller, 2000). While reproductively isolated, their distributional range and their ecological functions have a substantial overlap (Eisenring et al., 2016; Müller et al., 2000; Wattier et al., 2020). This allows treating them as two biological replicates of species to study effects of spatial network structure on the genetic diversity of (meta)populations (Altermatt et al., 2019; Eisenring et al., 2016).

### 2.2 Genetic data collection

We conducted the study in the river Rhine drainage within Switzerland, which encompasses about 28,000 km^2^ of its headwater area. We sampled *Gammarus fossarum* amphipods from 281 sites evenly and representatively spaced across the river Rhine headwaters between 2007 and 2015 by a kicknet approach. We morphologically identified all individuals to the species‐complex level (Altermatt et al., 2019). We further genotyped a subset of individuals of the *G*. *fossarum* complex using microsatellites (Westram et al., 2013), conventional 16S sequencing, or SNP pyrosequencing (Westram et al., 2011). We used the 16S mitochondrial gene sequence, or three SNPs therein, to reconstruct the realized distribution of both *G*. *fossarum* type A and type B (see Westram et al., 2011 for detailed methods). We relied on published and unpublished sequences and SNPs (Altermatt et al., 2014; Westram et al., 2013; Westram et al. unpublished data; Alther et al. unpublished data). We used the microsatellite data for subsequent population genetic analyses.

We extracted DNA for microsatellite analyses from complete individuals or their heads using a hotshot approach (Montero‐Pau et al., 2008). We amplified fragments using multiplex amplifications with the qiagen Multiplex PCR Kit chemicals. We used nine different microsatellite markers (gf08, gf10, gf13, gf18, gf19, gf22, gf24, gf27, gf28 sensu Westram et al. (2010)), specifically designed for *G*. *fossarum* and previously established in several studies (Altermatt et al., 2014; Westram et al., 2013) (note that microsatellite marker gf21 by Westram et al., 2010 was also included initially, but then discarded because of signs of null alleles, as also reported by Westram et al., 2013). We used primers in different concentrations (see Table 1 in Westram et al., 2010) in reaction volumes of 12.5 μl, with 6.25 μl of PCR Master Mix, 1.25 μl Q solution and 1 μl DNA template. The PCR consisted of an initial denaturation step at 95°C (15 min), 35 cycles at 94°C (30 s), 60°C (90 s), 72°C (60 s), and a final elongation step at 60°C (30 min). We diluted the resulting amplicons (1:20) and combined them with size standard (GeneScan 500 LIZ, Applied Biosystems). We sequenced fragments on an Applied Biosystems 3730xl DNA Analyser at the Genomic Diversity Centre of ETH Zurich, Switzerland. We analysed and manually edited the electropherograms using softgenetics genemarker software (v. 1.80). In total, we genotyped 3577 individuals. We used the microsatellite data to quantify genetic diversity within these two species. For the detailed molecular procedure on DNA extraction, microsatellite sequencing and microsatellite interpretation, see Westram et al. (2010), Westram et al. (2013), in which some of the individuals used here have already been analysed for different purposes.

**TABLE 1:** Chosen simulation parameters, explored values and their biological meaning

Parameter	Values	Meaning
Varying parameters		
*d*	0.001; 0.01; 0.1	Dispersal rate
*W*	0; 0.5; 1	Upstream movement probability
*K*	Fixed = 1000; scaled = (2; 8992)	Carrying capacity
Fixed parameters		
*μ*	0.0001	Mutation rate neutral alleles
*λ_0_*	2	Fecundity
*m*	0	Dispersal mortality

### 2.3 Spatial data preparation

The spatial riverine network used for the subsequent analysis represents a restricted version of the full Rhine network within Switzerland. We constructed a digital representation of the riverine network (Figure S1), based on a graph theory approach and following topological connectivity along the river lines. The riverine network is based on a 2 km^2^ subcatchment representation of streams and rivers of Switzerland (BAFU, 2012). Specifically, we interpreted these subcatchments as being nodes within the network and stream flow direction being directed vertices between these nodes. Based on the coordinates of the outlet site of each subcatchment (or on the centroid coordinates for headwater subcatchments), we constructed the riverine network connecting the outlet coordinates to each other. Distances from one subcatchment outlet to the adjacent downstream subcatchment outlet can be approximated as Euclidean distances on a small‐scale basis, and were included as vertex weights. Additionally, the graph object also contained information on the summed upstream catchment area for each subcatchment. The detailed methods of how we prepared the extensive graph object are described in Alther and Altermatt (2018).

We then restricted the analysis to the part of the riverine network that is actually inhabitable by either one or both of the studied amphipod species, based on an empirically validated cropping of the network. We used a database on amphipod occurrences in Switzerland with >2000 sites covered (Altermatt et al., 2019) to distinguish between nodes containing *G*. *fossarum* from unoccupied nodes. After preparation of the initial complete Rhine riverine network, we selected nodes (subcatchments) that contain one or both species of the *G*. *fossarum* complex and all their spatially interconnecting nodes (Figure S1). This resulted in the truncated riverine network that is empirically validated to be accessible and inhabitable to *G*. *fossarum*, containing 2401 nodes (referred to as *G*. *fossarum* network). The corresponding graph object is available on GitHub (see data accessibility section).

We subsequently mapped our microsatellite data from *G*. *fossarum* complex sites of Switzerland to the nodes of the prepared graph using arcgis 10.5.1 (ESRI Inc.). We refer to sites as nodes hereafter. Removing nodes that had <15 individuals successfully genotyped resulted in 95 nodes for subsequent analysis, harbouring 3319 individuals. The corresponding microsatellite data are available on GitHub (see data accessibility section). Data were available for 67 nodes for *G*. *fossarum* type A (2257 individuals) and 33 nodes for *G*. *fossarum* type B (1062 individuals), with five nodes having data on both (Figure S1). This preparation step resulted in a vector containing the corresponding node IDs where microsatellite data of either one or both species of the *G*. *fossarum* complex were available.

### 2.4 Stochastic simulation

To compare the empirical data to simulated data, we used a discrete‐time and stochastic individual‐based simulation (adapted from Fronhofer & Altermatt, 2017; Fronhofer et al., 2013, 2014). The model is analogous to the one used by Fronhofer and Altermatt (2017) and a detailed model description can be found there. In brief, we model a metapopulation of amphipods where we assume that local populations of amphipods compete for local and limited resources, which is captured by a Beverton‐Holt density‐regulation function (Beverton & Holt, 1957). The mean carrying capacity equals 1,000 individuals. Individuals are diploid and reproduce sexually (sex ratio: 0.5). Individuals perform natal, nearest‐neighbour dispersal, which is governed by a dispersal rate (*d*), and by the connectivity matrix of the metapopulation that is identical to the one derived for the empirical data (*G*. *fossarum* network). Most of the parameters of the simulation were fixed (see Table 1) but informed by the study system or the empirical methods used. We assume ten neutral, diploid loci that can take any of 100 different values as alleles to explore genetic diversity. The mutation rate of those alleles is set to 0.0001, which is in the range of empirically observed values (Estoup & Angers, 1998). We analysed two different mutational models: (1) a random mutational model, where, upon mutation, the value of the allele is randomly chosen with uniform probability from the 100 possible allele values; and (2) a stepwise mutational model (Kimura & Ohta, 1978), where, upon mutation, the value of the allele changes by +/− 1 with equal probability. In the latter case, we assume reflecting boundary conditions at 0 and 100.

We subsequently explored the full‐orthogonal parameter space along three free parameters. This included (1) three different dispersal rates (*d *= 0.001, 0.01, or 0.1), (2) three different upstream movement probabilities (*W*), and (3) two scenarios for the distribution of carrying capacities. Upstream movement probability described the effects of downstream water flow, where there was either no upstream movement (*W *= 0), where upstream and downstream movements were equally likely (*W *= 1), or where downstream movements was twice as likely as upstream movement (*W *= 0.5). Carrying capacity (*K*) was either identical for all nodes (*K* = 1,000 per node), or it scaled with the square‐root of the total catchment area as described by Rodriguez‐Iturbe and Rinaldo (1997), such that the highest carrying capacity corresponded to the most downstream node while keeping the total metapopulation size constant (2401 nodes × 1000 individuals). All simulations were run with ten replicates, and for 10,000 generations each, which is sufficient to reach (quasi‐)equilibrium (checked by plotting dynamics over 10,000 generations, data not shown). All population genetic analyses where performed on the individuals of the last generation (*t *= 10,000). The explored parameter space is detailed in Table 1. The simulation code is available on GitHub (see data accessibility section).

### 2.5 Statistical analyses

For the spatial data (explanatory variables), we calculated a series of network metrics in order to identify the influence of network topology based on the *G*. *fossarum* network containing 2401 nodes and the subset of 95 nodes with microsatellite data available. The calculations were done in r 3.6.1 (R Core Team, 2019) with the package igraph 1.2.4.2 (Csárdi & Nepusz, 2006). The network metrics for single nodes were upstream distance from the outlet node, total upstream catchment area, directed and undirected betweenness centrality, directed and undirected closeness centrality, and degree centrality. Directed and undirected measures correspond to either considering flow direction, or ignoring it. The upstream distance corresponds to the instream distance from the outlet node of the riverine network near Basel, where the river Rhine continues to France and Germany and gets into a different biogeographic zone, thereby naturally separating the catchments considered here. The closeness centrality corresponds to the reciprocal of the sum of the distances between a node and all other nodes in the riverine network. We standardized the closeness centrality (*c)* for analysis using the following approach: $\frac{c_{i}‐\min\left(c\right)}{\max\left(c\right)‐\min\left(c\right)}$. In biological terms, all these network metrics capture the connectivity of single populations to the other populations, with higher values translating to reduced connectivity.

For the genetic data (response variables), we calculated mean allelic richness, observed heterozygosity, expected heterozygosity, and pairwise genetic differentiation (Nei *F*_ST_; Nei, 1987) for *G*. *fossarum* type A and *G*. *fossarum* type B using the packages hierfstat 0.04–22 (Goudet, 2005) and adegenet 2.1.1 (Jombart, 2008; Jombart & Ahmed, 2011) within r 3.6.1 (R Core Team, 2019). We calculated these measures for both the empirical data and the simulated data.

Prior to modelling the empirical genetic response variables, we excluded highly correlated explanatory variables (Kendall's Tau >0.8; Figure S2), specifically undirected betweenness centrality, degree centrality, and directed closeness centrality. Additionally, we log‐transformed the total upstream catchment area and the directed betweenness centrality to reduce skewedness (Figure S3). We modelled the genetic response variables separately using linear models (LM) using the lm() function since the residuals followed a Gaussian distribution. We included all network metrics (upstream distance, total catchment area, directed betweenness centrality, undirected closeness centrality) and species as factors with all higher‐level interaction terms. We applied backward stepwise selection (function step()) using AIC scores to reduce interaction terms. Additionally, we ran models without interaction terms and selected the most parsimonious one with a dredge approach using function dredge() from mumin package (Bartoń, 2020) based on AICc scores. We also calculated variance‐inflation factors (VIF) for all explanatory variables in the interaction and simple linear models in order to detect signals of strong collinearity. We selected the overall best fitting model for each genetic response variable comparing the AIC score of the selected interaction model and the selected model without interaction terms, additionally requiring variance inflation factors to be around 1–2. We additionally conducted separate LMs for all explanatory variables individually for a qualitative comparison. Figures were plotted using the fitted values retrieved from the separate LMs with only one explanatory variable each and species included if AIC was lower, using the predict() function. We modelled pairwise genetic differentiation and isolation‐by‐distance using linear models, with instream distance as an explanatory variable, including species as factor. We ran models with or without interaction, with untransformed or log‐transformed *F*_ST_ values, or including a power term. Five negative *F*_ST_ values arose from a calculation artefact and we manually set them to zero prior to modelling. For the models using a power term and for selecting the best fitting one, we ran 100 models varying the power term from 0 to 1 in steps of 0.01, subsequently checking for minimum AIC score. Finally, we selected the overall best fitting model for *F*_ST_ based on AIC scores. We retrieved F‐test statistics and *R* ^2^ as coefficient of determination for all models directly from the lm() function. To assess isolation‐by‐distance, we used a Mantel test using the function mantel() from the package vegan 2.5‐6 (Oksanen et al., 2019).

To assess which parameter combination for the simulations best fit the observed data, we correlated simulated to empirical population genetic variables (mean allelic richness, mean observed heterozygosity, expected heterozygosity, and genetic differentiation). If simulation results and empirical data were identical, they would lie on the 1:1 diagonal line when plotting empirical versus simulated data from identical nodes (Figure S4a). So in order to formalize simulation‐empirical data discrepancies we calculated deviations from this 1:1 line fit using the perpendicular offset (distance). This approach is straightforward and requires very few assumptions. However, unlike a conventional correlation, this also assesses the fits to both the range (intercept) and the explicit arrangement (slope) of response variables (see Figure S4 for different scenarios of correlations 1, –1, and 0). We used the sum of perpendicular offsets (SPO) as well as the median of the perpendicular offsets (MPO) as goodness‐of‐fit measures. The SPO takes into account the overall spread of simulated values from their empirical counterpart, where a larger SPO indicates a poorer fit (e.g., Figure S4b vs. S4d). Considering the median using MPO partially takes into account outliers of individual nodes. An MPO closer to zero indicates that most of the simulated values fell close to the empirical counterpart. Since the perpendicular offset does not take into account if the offset is above or below the vertical (1:1) line, we additionally computed the directed median of the perpendicular offset (DMPO, e.g., Figure S4b vs. S4c). To assess which specific parameter value for each of the varying parameters (dispersal rate, upstream movement probability, scaling of carrying capacity) generally best fitted to the observed data, we compared simulations with a specific parameter value to all corresponding simulations with the remaining parameter values of the same type. Specifically, we subtracted their goodness‐of‐fit measures (both, SPO and MPO) and assessed the sign (positive or negative). For example, the SPO for mean allelic richness with *d *= 0.001, *W *= 0, *K *= 0 was subtracted from the SPO for mean allelic richness with *d *= 0.01; *W *= 0; *K *= 0. If the simulation fit was higher for *d *= 0.001, this subtraction would result in a negative sign. Repeating this across all simulation combinations and all response variables (mean allelic richness, mean observed heterozygosity, expected heterozygosity) resulted in a fraction of comparisons with a negative sign. If this fraction was higher than 0.5, the former parameter value was considered superior, since models with this parameter value combination performed better in more than half of all comparisons. We calculated the SPO, the MPO, and the DMPO using our own functions, included in the analysis script available on GitHub (see data accessibility section). All calculations were done in r version 3.6.1 (R Core Team, 2019).

## 3 RESULTS

### 3.1 Population genetics of the *Gammarus fossarum* complex

**FIGURE 1:**
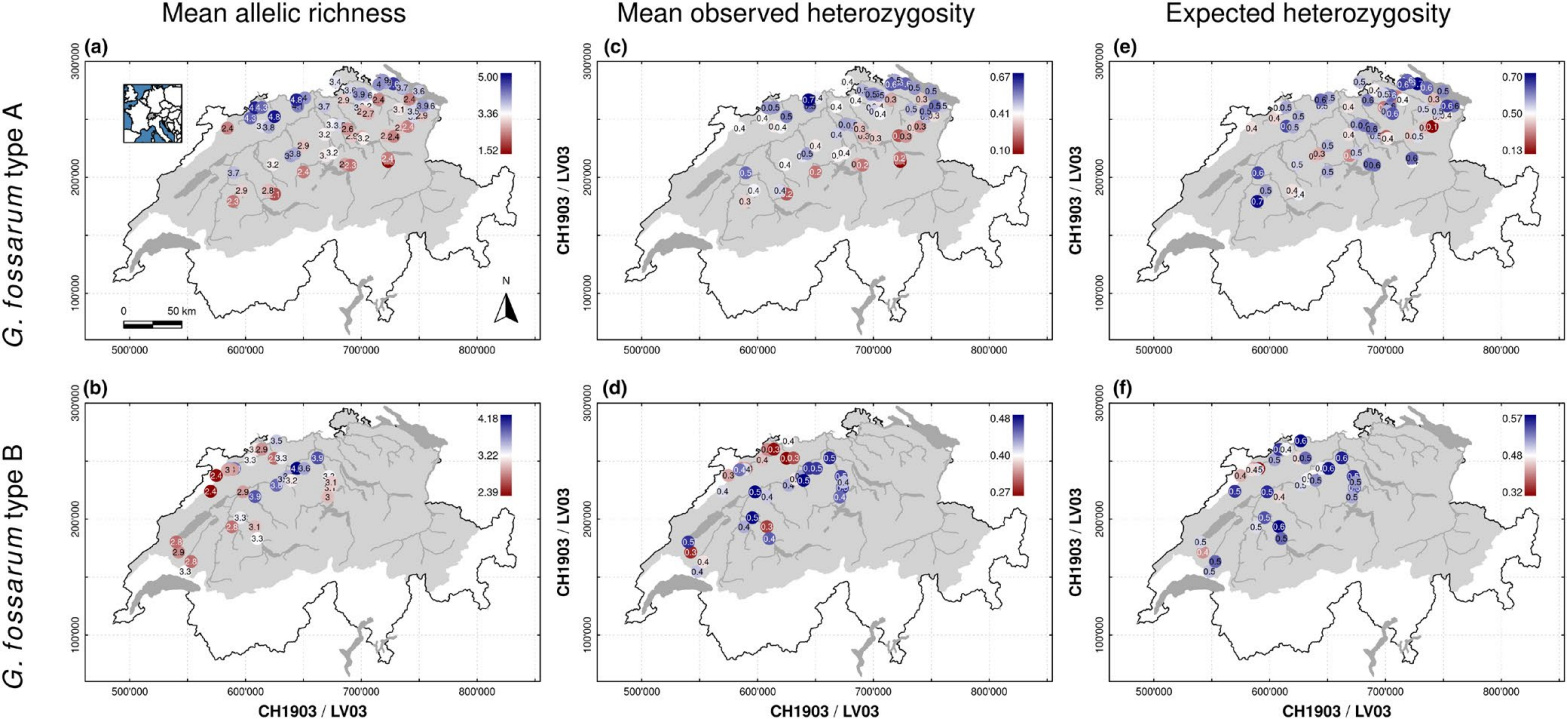
Empirically assessed mean allelic richness (a, b), mean observed heterozygosity (c, d), and expected heterozygosity (e, f) of the two cryptic amphipod species *Gammarus fossarum* type A (a, c, e) and *G*. *fossarum* type B (b, d, f) in the river Rhine network in Switzerland. The river Rhine catchment is highlighted by the grey contour area, with the major river and lakes indicated. Both species of the *Gammarus fossarum* complex are widely distributed at elevations below 1000 m a.s.l., with type A being more common in the North Eastern part of the catchment, and type B more common in the Western part. Geodata source: Federal Office of Topography & Federal Office for the Environment

Empirically assessed mean allelic richness ranged from 1.5 to 5.0 (mean: 3.3; median: 3.4; SD: 0.7) for *G*. *fossarum* type A, and from 2.4 to 4.2 (mean: 3.2; median: 3.2; SD: 0.4) for *G*. *fossarum* type B. Both species showed spatial gradients of within‐population genetic diversity when using mean allelic richness as a diversity metric, with higher values in more central nodes, visually apparent when plotted on a map (Figure 1a,b). Empirically assessed mean observed heterozygosity ranged from 0.10 to 0.67 (mean: 0.41; median: 0.41; SD: 0.11) for *G*. *fossarum* type A, and from 0.27 to 0.48 (mean: 0.39; median: 0.40; SD: 0.06) for *G*. *fossarum* type B. The geographic distribution of mean observed heterozygosity was only apparent in *G*. *fossarum* type A (Figure 1c) but not in *G*. *fossarum* type B (Figure 1d). Expected heterozygosity ranged from 0.13 to 0.70 (mean: 0.48; median: 0.50; SD: 0.12) for *G*. *fossarum* type A, and from 0.32 to 0.56 (mean: 0.48; median: 0.48; SD: 0.06) for *G*. *fossarum* type B. A clear geographic distribution of expected heterozygosity was neither apparent in *G*. *fossarum* type A (Figure 1e) nor in *G*. *fossarum* type B (Figure 1f). Genetic differentiation quantified as *F*_ST_ ranged from 0 to 0.758 (mean: 0.372; median: 0.373; SD: 0.138) for *G*. *fossarum* type A, and from 0 to 0.384 (mean: 0.178; median: 0.165; SD: 0.103) for *G*. *fossarum* type B, comparable to Westram et al. (2013).

**FIGURE 2:**
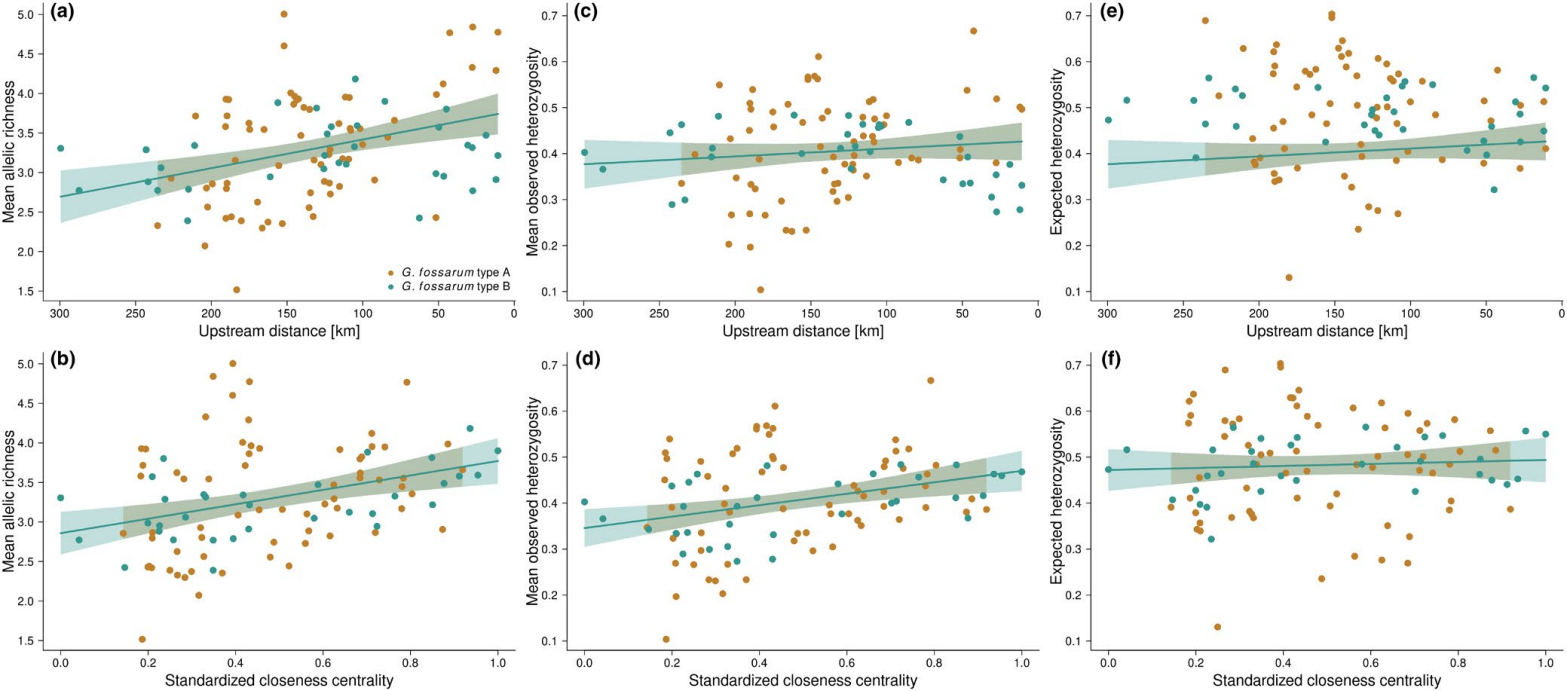
Empirically assessed mean allelic richness, mean observed heterozygosity, and expected heterozygosity of both species of the *Gammarus fossarum* complex (type A: orange points, type B: cyan points) with respect to different river network metrics. Raw data points as well as model fits of linear models (solid lines) and their 95% confidence intervals (shading) are given. Mean allelic richness as a function of (a) upstream distance from the outlet node within the riverine network and as a function of (b) standardized undirected closeness centrality. Mean observed heterozygosity as a function of (c) upstream distance from the outlet node within the riverine network and as a function of (d) standardized undirected closeness centrality. Expected heterozygosity as a function of (e) upstream distance from the outlet node within the riverine network and as a function of (f) standardized undirected closeness centrality

Linear models explained allelic richness and observed heterozygosity by network topology. Models with higher‐level interactions performed worse than models without interactions based on AIC and VIF (data not shown) and we used the latter. Mean allelic richness was best explained by upstream distance in combination with directed betweenness centrality and undirected closeness centrality (Figure 2a; *F*_3,96_ = 8.5; *p *< .001; *R* ^2^_adj_ = 0.19). It significantly decreased with upstream distance from the outlet node within the riverine network in both species of the *Gammarus fossarum* complex. This translates to higher allelic richness in more central and better‐connected nodes of the network. In addition, higher carrying capacity generally increased allelic richness, both in the simulations as well as in the empirical data (data not shown). Mean observed heterozygosity was best explained by undirected closeness centrality and directed betweenness centrality (Figure 2d; *F*_2,97_ = 7.94; *p* < .001; *R* ^2^_adj_ = 0.12). Observed heterozygosity was higher in more central and better‐connected nodes of the network, hence it increased with increasing closeness centrality and higher betweenness centrality for both species of the *G*. *fossarum* complex. Expected heterozygosity could not be explained by any network metric (Figure 2e–f), in neither of the two species of the *G*. *fossarum* complex.

*F*_ST_ significantly increased with increasing instream distance between nodes in the network, which is a clear signal of isolation‐by‐distance (Figure 5). The corresponding Mantel tests for both species using 1000 permutations separately confirmed this finding (Pearson *r *= 0.623 and 0.541; both *p *< .001). A visual check of the data suggested a case‐IV relationship (sensu Hutchison & Templeton, 1999), with a monotonically increasing *F*_ST_ up to a certain instream distance, after which no such relationship persists. The scatter (variance) increased with instream distance. A interaction LM including species as a factor with a power term of 0.55 for instream distance captured well the saturating response, with instream distance, species, and their interaction being highly significant (Figure 5; *F*_3,2735_ = 1094.0; *p *< .001; *R* ^2^_adj_ = 0.55).

### 3.2 Simulation – data comparison

**FIGURE 3:**
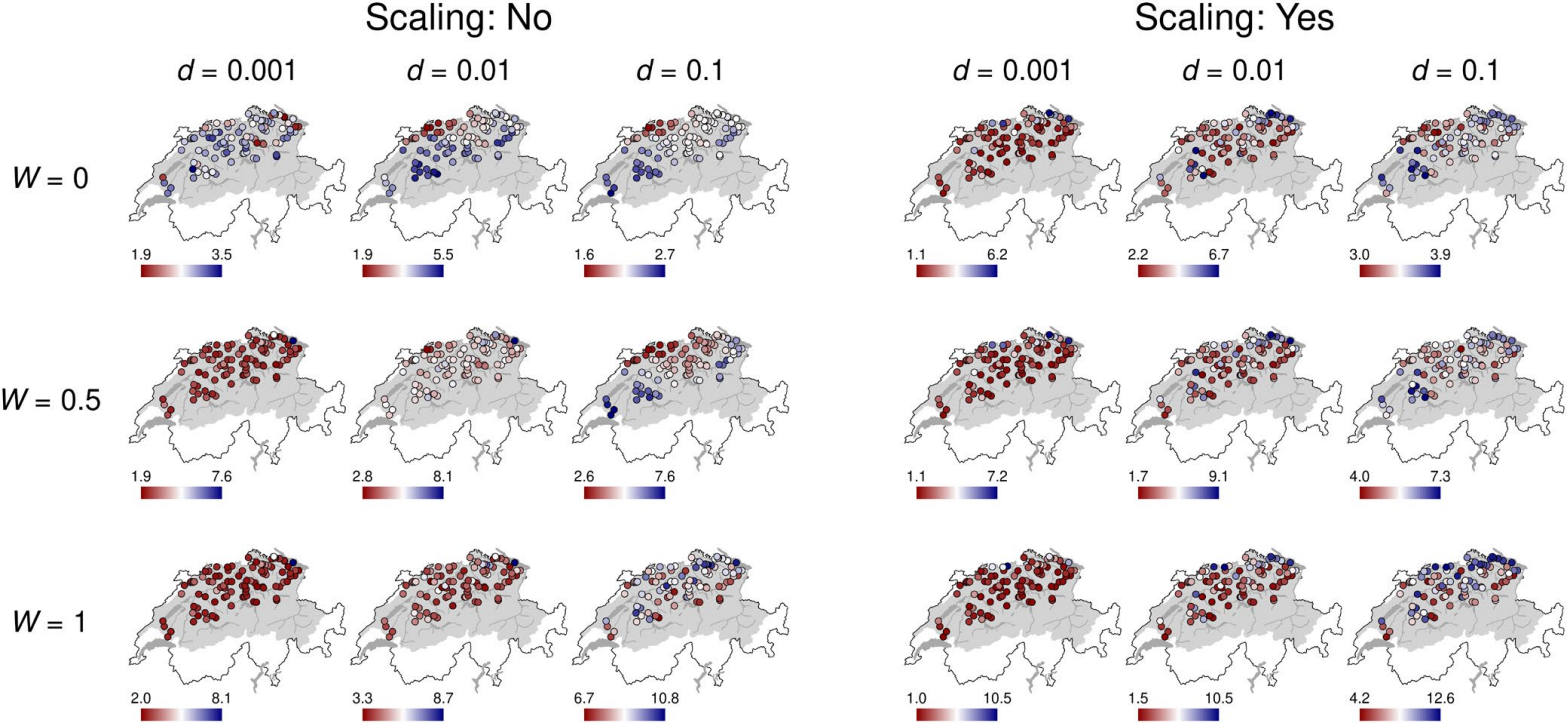
Maps depicting the predicted mean allelic richness for all 18 stochastic simulation scenarios show different spatial structuring along the Rhine riverine network of Switzerland. The gradient legends show mean allelic richness. Their scale is individually adjusted in each map for the best representation of spatial structuring. *d* is the dispersal rate and *W* is the upstream movement probability (*W *= 0 corresponds to no upstream movement, *W *= 1 represents equal probability of moving up‐ and downstream). The corresponding figure for mean observed heterozygosity is given in Figure S5, the one for expected heterozygosity is given in Figure S6. Geodata source: Federal Office of Topography & Federal Office for the Environment

The stochastic simulations assuming a stepwise mutation model resulted in highly differentiated spatial patterns of population genetic diversity depending on the set of parameter values used (Figure 3, Figure S5 and Figure S6). Of the three different parameters considered (dispersal rate, upstream movement probability, scaling of carrying capacity), upstream movement probabilities generally showed the strongest effect on the response variable with respect to the parameter space covered, with unidirectional movement (*W *= 0) generally resulting in better model fits (Figure S7). Using different dispersal rates in the stochastic simulations resulted in comparable effects on the genetic diversity (shift of overall median of perpendicular offsets), with low dispersal rates generally resulting in better model fits (Figure S8). Scaling the carrying capacity (*K* = 1) consistently worsened the model fits and showed a smaller effect on the response variable compared to upstream movement probability and dispersal rate (Figure S9).

Comparing the simulation outputs to empirical data showed that simulations based on low dispersal rates (*d* = 0.001) outperformed the corresponding ones with higher dispersal rates (*d* = 0.01 or *d* = 0.1) according to both goodness‐of‐fit measures (smaller SPO and smaller MPO) in 73% of the cases (105 comparisons out of 144). Simulations with no upstream dispersal (*W* = 0) outperformed simulations allowing some level of upstream dispersal (*W* = 0.5 and *W* = 1) in 93% of the cases (134 comparisons out of 144). Simulations with no scaling of carrying capacity (*K* = 0) outperformed their counterparts with scaling in 57% of the cases (62 comparisons out of 108).

**FIGURE 4:**
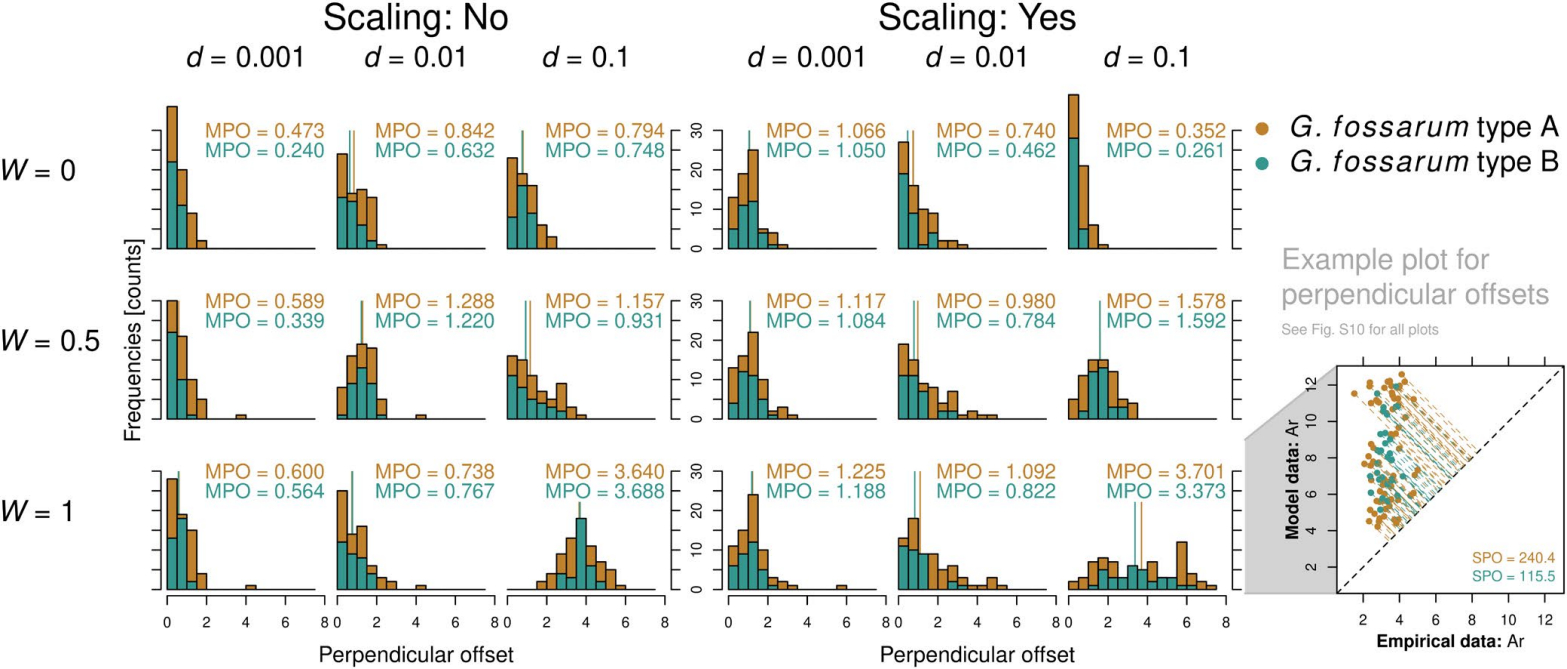
Histograms and medians of the perpendicular offsets (MPO) between all 18 stochastic simulation scenarios and the empirically assessed mean allelic richness values for both species of the *Gammarus fossarum* complex (type A: orange colour, type B: cyan colour). *d* is the dispersal rate and *W* is the upstream movement probability (*W *= 0 corresponds to no upstream movement, *W *= 1 represents equal probability of moving up‐ and downstream). The more left‐skewed a distribution, the better the fit of simulated values to empirical data. The example plot on the right hand side illustrates the concept of perpendicular offsets (compare to Figure S10 for all 18 scenarios)

The best fitting simulations for allelic richness according to SPO for both species were based on high dispersal rates (*d* = 0.1), no upstream movement (*W* = 0), and scaling of carrying capacity (Figure 4 and Figure S10, Table S11). However, the next best fitting simulations were based on low dispersal rate and no scaling of habitat capacity, indicating interactions between parameters. For mean observed heterozygosity we found that the best fitting simulation according to SPO was based on low dispersal rates (*d* = 0.001), no upstream movement (*W* = 0), and no scaling of carrying capacity in *G*. *fossarum* type A. In *G*. *fossarum* type B, the best simulation fit required low dispersal (*d* = 0.001), moderate upstream movement (*W* = 0.5), and no scaling of carrying capacity (Figure S12 and S13, Table S14). For expected heterozygosity we saw that the best fitting simulation according to SPO was also based on low dispersal rates (*d* = 0.001), no upstream movement (*W* = 0), and no scaling of carrying capacity in *G*. *fossarum* type A, whereas in *G*. *fossarum* type B, the best simulation fit required low dispersal (*d* = 0.001), moderate upstream movement (*W* = 0.5), and no scaling of carrying capacity (Figure S15 and Figure S16, Table S17).

When accommodating for outlier nodes by comparing MPO, the best fitting simulations for allelic richness for *G*. *fossarum* type A was based on high dispersal rates (*d* = 0.1), no upstream movement (*W* = 0), and scaling of carrying capacity (Figure 4 and Table S18). For mean observed heterozygosity, it was based on low dispersal rates (*d* = 0.001), no upstream movement (*W* = 0), and no scaling of carrying capacity (Figure 4 and Table S19). Hence, SPO and MPO were congruent in *G. fossarum* type A. With *G*. *fossarum* type B, the best fitting simulation for allelic richness required low dispersal (*d* = 0.001), no upstream movement (*W* = 0), and no scaling of carrying capacity (Figure 4 and Table S18), deviating from SPO. For mean observed heterozygosity, it required low dispersal (*d* = 0.001), moderate upstream movement (*W* = 0.5), and no scaling of carrying capacity (Figure S12 and Table S19) as with SPO. For expected heterozygosity, the best fitting simulation according to MPO was based on high dispersal rates (*d* = 0.1), no upstream movement (*W* = 0), and scaling of carrying capacity in *G*. *fossarum* type A, deviating from SPO, but low dispersal (*d* = 0.001), moderate upstream movement (*W* = 0.5), and no scaling of carrying capacity in *G*. *fossarum* type B (Figure S15 and Table S20), as with SPO. The directed median perpendicular offsets (DMPO) showed that simulations with low dispersal (*d* = 0.001) mostly fell within the range of observed empirical data assuming no scaling of carrying capacity. With scaling of carrying capacity, simulations with low dispersal rate (*d* = 0.001) consequently underestimated genetic diversity. Simulations with moderate dispersal rates (*d* = 0.01) had a small median perpendicular offset but a higher variance compared to their unscaled counterparts. High dispersal rates (*d* = 0.1) generally overestimated all three measures of population genetic diversity (Figures S21, S22, and S23), except when a high dispersal rate (*d* = 0.1) was coupled with no upstream dispersal (*W* = 0).

Comparing simulated and empirically observed genetic differentiation *F*_ST_ (Figure S24) revealed that some simulated IBD patterns generally fit the empirical data, while others were completely off. Simulations based on moderate dispersal rates (*d* = 0.01) outperformed the corresponding ones with lower or higher dispersal rates (*d* = 0.001 or *d* = 0.1) according to SPO and MPO in 76% of the cases (32 comparisons out of 42). Simulations with no upstream dispersal (*W* = 0) outperformed simulations with upstream dispersal (*W* = 0.5 and *W* = 1) in 64% of the cases (28 comparisons out of 44). Simulations with no scaling of carrying capacity (*K* = 0) outperformed their counterparts in 90% of the cases (27 comparisons out of 30). The best fitting simulations for *F*_ST_ according to SPO for *G*. *fossarum* type A was based on moderate dispersal rates (*d* = 0.01), upstream and downstream movements being equally likely (*W* = 1), and no scaling of carrying capacity (Table S26). With *G*. *fossarum* type B, the best fitting simulation required high dispersal (*d* = 0.1), equal up‐ and downstream movement probabilities (*W* = 1), and no scaling of carrying capacity (Table S26). When considering outliers by comparing MPO, the best fitting simulations were identical to the ones considering SPO (moderate to high dispersal rates (*d* = 0.01 or *d* = 0.1), upstream and downstream movements being equally likely (*W* = 1), and no scaling of carrying capacity (Figure S25 and Table S27). Better fits of simulations without scaling of carrying capacity was mostly due to the smaller variance of the perpendicular offset (Figure S25).

Comparing the simulations based on a random mutational model to the empirical data also confirmed the major influence of upstream movement probability and dispersal rate. However, the effect of dispersal rate was stronger than the effect of upstream movement probability. Models with low dispersal rates (*d* = 0.001) outperformed the ones with higher dispersal rates (*d* = 0.01 or *d* = 0.1) in 88% of the cases, simulations with no upstream dispersal (*W* = 0) outperformed simulations allowing some level of upstream dispersal (*W* = 0.5 and *W* = 1) in 76% of the cases, and simulations with no scaling of carrying capacity outperformed their counterparts with scaling in 65% of the cases. The best models according to SPO and MPO always required low dispersal rates (Figures and Tables S28–S52).

## 4 DISCUSSION

Combining stochastic simulations and empirical data from two amphipod species within a large riverine network, we showed a clear signature of spatial configuration and connectivity on their genetic diversity across both metapopulations. The stochastic simulations embraced the specific nature of riverine networks by using a realistic representation of the riverine network (Carraro et al., 2020) with 2401 nodes, helping to dissect the relevant processes explaining the genetic diversity.

Past theoretical (Blanchet et al., 2020; Fronhofer & Altermatt, 2017; Morrissey & de Kerckhove, 2009; Paz‐Vinas et al., 2015) and empirical (Fourtune et al., 2016; Hughes et al., 2009; Meffe & Vrijenhoek, 1988; Paz‐Vinas et al., 2018; Seymour et al., 2016) studies have postulated specific effects of riverine network configuration on the genetic diversity of aquatic organisms. Here, we assessed empirical data across large natural metapopulations of freshwater amphipods (Figure 1), and found that some measures of local genetic diversity such as allelic richness and mean observed heterozygosity were higher in more central nodes of the network (Paz‐Vinas et al., 2015), while expected heterozygosity showed no clear imprint of network position. This generally supports theoretical expectations when the network entails some dispersal limitation. Allelic richness was best explained by upstream distance from the outlet node and directed betweenness centrality, whereas mean observed heterozygosity was best explained by undirected closeness centrality and directed betweenness centrality (Figure 2). The three different measures of genetic diversity hence were each best explained by different network metrics. This different relevance of different network metrics on these measures of genetic diversity implies biologically different processes (or a different focus of the measures). Upstream distance probably captures aspects of colonization legacy (either on the species itself or on some of its competitors), which may correlate better with larger biogeographic regions. This legacy seems best captured by allelic richness where higher values suggest a higher evolutionary potential at the population level. By contrast, closeness centrality better captures overall connectivity within the metapopulation, hence the more short‐term effect of dispersal seems to manifest in the mean observed heterozygosity, the proportion of heterozygotes in the sample.

So while network position partially explained allelic richness (and mean observed heterozygosity), expected heterozygosity could not be explained. However, nodes with low closeness centrality showed a smaller mean observed heterozygosity compared to their expected heterozygosity (Figure 2d,f). Hence, the average proportion of heterozygotes did not match the assumption that the population is in Hardy‐Weinberg equilibrium (randomly mating), potentially indicating inbreeding or recent bottleneck events. Hence, either riverine network position may have influenced inbreeding or bottleneck events, or the mismatch may be due to colonization legacy. On one hand, the latter seems less likely as there was no apparent difference when plotting mean observed heterozygosity and expected heterozygosity against upstream distance (Figure 2c,e). On the other hand, overall empirical F_IS_ values were 0.13 for *G*. *fossarum* type A and 0.22 for *G*. *fossarum* type B, respectively, implying some degree of inbreeding. A preliminary analysis of M‐ratios across all loci of the two species revealed all values being smaller than 0.68 (data not shown), being indicative of a recent reduction of population sizes (Garza & Williamson, 2001).

**FIGURE 5:**
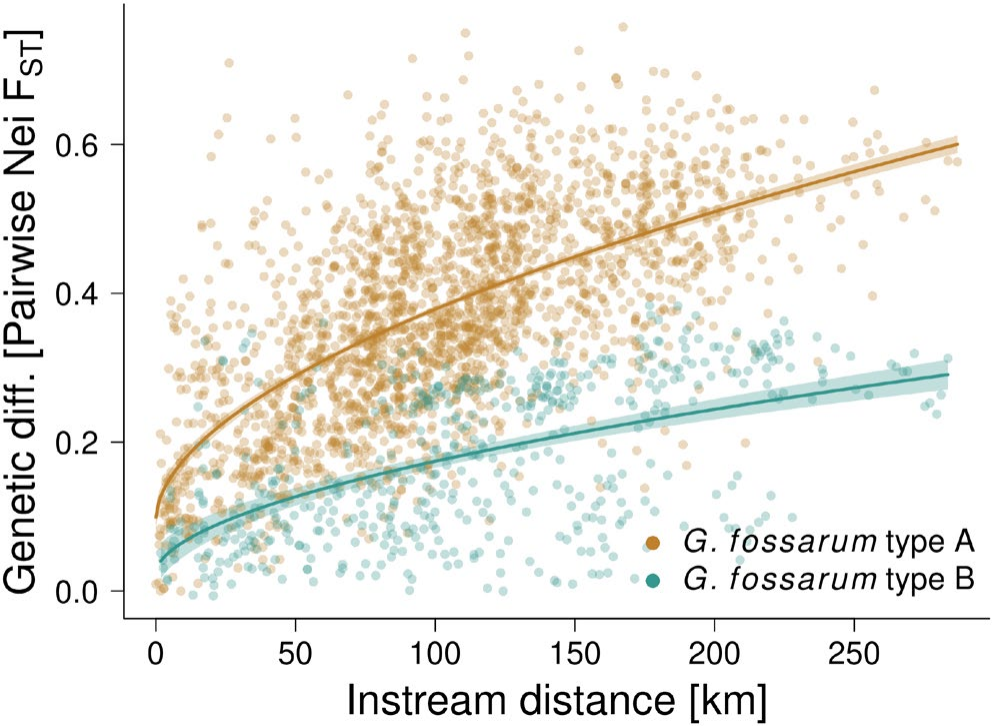
Isolation‐by‐distance pattern, shown as genetic differentiation *F*_ST_ increasing with increasing instream distance between nodes of the riverine network (Lines: LM with power term; shading depicts 95% confidence interval). The data support a case‐IV relationship (sensu Hutchison & Templeton, 1999)

Genetic differentiation between populations increased with increasing instream distance (Figure 5). We confirmed the isolation‐by‐distance pattern in the studied amphipod species over hundreds of kilometres across a large riverine network (Westram et al., 2013). Overall, genetic diversity in *Gammarus fossarum* type A and type B did not differ significantly, despite being functionally (Eisenring et al., 2016) and phylogeographically distinct, with different colonization history in Switzerland and Europe in general (Wattier et al., 2020; Westram et al., 2013). This suggests that the riverine network entails similar constraints on the genetic diversity of organisms with comparable life histories.

We then compared the empirical data to stochastic simulations ran under different scenarios, to identify the main drivers of population genetic diversity (Figure 3). Importantly, the simulations ran on the same, and spatially realistic, graph representation of the empirical river network from where the population genetics data originated, whereas previous studies on population genetic diversity in riverine networks strongly relied on more artificial representations of riverine networks (e.g. Paz‐Vinas et al., 2015). The comparison using the sum of perpendicular offsets (SPO; Figures S10, S13, and S16) and the median of perpendicular offsets (MPO; Figures 4, S12, and S15) showed that simulations with no upstream movement matched best the observed patterns. We varied upstream movement considerably in our simulations, from upstream and downstream movements being equally likely (*W *= 1) to excluding upstream movement completely (*W *= 0). This biologically meaningful change did result in considerable differences in simulation matches. Among the best fitting scenarios, we usually find simulations without or moderate upstream movement, (Tables S11, S14, S17–S20). This highlights that dispersal asymmetry clearly contributes to explaining the observed spatial distribution of genetic diversity of the particular species in the studied river basin. The riverine network imposes a low and restricted connectivity compared to a lattice‐type landscape and seems to reinforce the role of dispersal, rendering its directionality an important component, probably interacting with dispersal rate.

Dispersal rate showed a comparable and consistent response, with simulations based on a low dispersal rate (*d* = 0.001) generally outperforming simulations with higher dispersal rates (Figure S8). This strongly suggests that the magnitude of dispersal has a pronounced effect on population genetic diversity. Higher dispersal rates may homogenize populations (Bohonak, 1999). Hence, observing clear geographic patterns in the genetic diversity of a metapopulation suggests moderate to low dispersal or connectivity. However, even the highest dispersal rates used in our model (*d* = 0.1) led to clear geographic pattern in population genetic diversity in combination with restricted or moderate upstream movement (Figure 3, S5, and S6). Thus, the comparison between empirical data and simulations support the notion that riverine networks impose such strong restrictions on connectivity between nodes that spatial genetic diversity may be maintained despite high dispersal rates. Contrary to these general observations, the best model fits for allelic richness were based on high dispersal rate coupled with scaling of habitat capacity and no upstream movement according to SPO and MPO in *Gammarus fossarum* type A and according to SPO in *Gammarus fossarum* type B. This result contrasts to the general picture, where lower dispersal rates and no scaling of habitat capacity usually perform better. This finding is most probably caused by the good fit to the range of empirically observed allelic richness values, whereas the spatial configuration seems less fitting (Figure S10). An alternative explanation suggests that in order to maintain high allelic richness in central parts of the riverine network, either one assumes similar population sizes coupled with low connectivity (resulting from low dispersal rate and asymmetric dispersal; see also discussion about random mutational model), or one assumes larger populations downstream coupled with high dispersal rates, especially upstream. Note that these last conclusions depend on the mutational model we assume. They hold only in the stepwise mutational model and not in the random mutational model. The differences between the two mutational models mainly stem from the fact that in the stepwise mutational model there is a 50% chance of back‐mutations since mutations always happen to neighbouring allele values which reduces genetic diversity in comparison to the random mutational model (where back‐mutations have a probability of 1/100 in our case). Overall, this result clearly supports the notion that the genetic diversity in the fluvial network is shaped by the interaction between the parameters, that is, if dispersal rate is high but there is no upstream movement, the scaling of habitat capacities becomes important.

When comparing simulations with identical dispersal rates, those including restricted upstream movement (lower values of *W*) fitted better than simulations with no movement directionality (Figure S7), indicating the influence of asymmetric gene flow in generating the observed patterns (Fraser et al., 2004). With low dispersal rates, the upstream movement probability did not play a major role in improving the model fit. Whereas a previous study already suggested an effect of asymmetric gene flow on the genetic diversity patterns in *G*. *fossarum* (Alp et al., 2012), fully directional gene flow seems unrealistic, given the subtle differences within simulations using the same dispersal rate (Morrissey & de Kerckhove, 2009). Interestingly, results for *G*. *fossarum* type A slightly differed from *G*. *fossarum* type B. In the former, the best fitting simulations usually relied on asymmetric dispersal (W = 0), whereas for the latter they often required some upstream dispersal (W = 0.5). This could be indicative of their slightly different ecological role, where *G*. *fossarum* type B tends to be adapted to larger streams and being rather mobile, whereas *G*. *fossarum* type A represents a headwater specialist (Altermatt et al., 2019; Eisenring et al., 2016). One major unknown, requiring further empirical studies, is how mobile the studied species actually are; often, they are considered comparably poor dispersers (Elliott, 2003; Weiss & Leese, 2016) or mostly transported passively by drift, while some studies suggest them being rather mobile (Meijering, 1972; Žganec et al., 2013). Our results do not allow us to draw a conclusion regarding the process that drives upstream movement, and how much of the dispersal is active versus passive (e.g., downstream transport or dislocation by vectors). Possibly, our view of the riverine network being downstream oriented is not what the studied amphipods experience, and their benthic life‐style may mitigate downstream flow considerably (Statzner & Holm, 1989).

Surprisingly, scaling the carrying capacity of nodes with the square‐root of the total catchment area (Ozerov et al., 2012), and thus making the habitat capacity presumably more realistic, lowered simulation fits to empirical data when compared to the unscaled counterparts in almost 60% of the simulation cases, irrespective of the species (Figure S9). The scaling generally increased the range of the response values, making SPO and MPO increase. In the empirical data, however, we did not find comparably high levels of allelic richness. We thus cannot exclude that the chosen scaling function does not correspond to the realized distribution of carrying capacities nor that the carrying capacity does not scale at all. Long‐term data from the lower part of the Rhine in Switzerland, that is, in a very large stream, indicate very high densities of *G*. *fossarum* species compared to commonly observed densities in upstream reaches (Mürle et al., 2008), contradicting the nonscaling of carrying capacity. In addition, *G*. *fossarum* type B is a slightly more tolerant species and can also be found in anthropogenically more affected streams, while *G*. *fossarum* type A is the typical amphipod species of near‐natural headwater streams (Eisenring et al., 2016). Hence, we expected *G*. *fossarum* type B to be more common in larger streams (i.e., larger total catchment area) and therefore showing less variance in occupied carrying capacities. However, a simple two‐sample Kolmogorov‐Smirnov test indicated that the samples we had at hand did not differ significantly in their total catchment area and consequently their stream width (data not shown). This could explain that the model fits were worse in both *G*. *fossarum* type A and type B when scaling the carrying capacity.

Comparing simulated *F*_ST_ values to the empirically observed ones showed that the best matches required different parameter values than for within population genetic diversity (Figures S24 and S25, Table S26 and S27). While the low dispersal rates (*d *= 0.001), required to match simulations to empirically observed within population genetic diversity, resulted in too high genetic differentiation, high dispersal rates (*d *= 0.1) turned out to result in too low values (Figure S24). The influence of upstream movement probability seemed to be overruled by the other two parameters (Figure S24). But again, simulations assuming no scaling of carrying capacity usually performed better than their scaled counterpart, confirming our finding from the within population genetic diversity. Therefore the main difference between comparisons of the within and between population genetic diversity was the required dispersal rate in order to maximize simulation fit to empirical data. This could be indicative that although *F*_ST_ values suggested an intermediate dispersal rate, recent bottleneck events might have rendered the local populations less diverse. Generally, the simulated *F*_ST_ values saturated faster than the empirically observed ones, highlighting that the empirical differentiation is probably driven by additional factors. Finally, we note that the observed *F*_ST_ values are considerably high, as has also been found and noted by Westram et al. (2013). Actually, some of the values are so high that a hidden diversity of genetically incompatible lineages cannot be excluded (see also Wattier et al., 2020 on the cryptic diversity wit*hin Gammarus foss*arum). However, the study of such hidden cryptic diversity may require further markers not yet available.

Simulations that were based on the random mutational model resulted in slightly different fits to empirical data, with dispersal rate being the main driver and upstream movement probability contributing to the observed pattern (Figures and Tables S28–S52). Here, the best models always relied on low dispersal rate, often coupled with no upstream movement and no scaling of habitat capacity (Tables S32, S33, S38, S39, S44, S45). Therefore, if mutations arise randomly with respect to the locally occurring alleles, low dispersal rate presumably maintains genetic diversity by lowering exchange between populations. If coupled with asymmetric movement probability, this effect seems often pronounced.

In conclusion, our study showed a pronounced effect of upstream movement probability and dispersal rate within the riverine network on the genetic diversity of two amphipod species across a large spatial extent. The impact of assuming increasing local population sizes with increasing downstream distance was less clear cut but overall probably less important in shaping population genetic diversity. This suggests that for understanding and protecting genetic diversity of strictly riverine organisms, network connectivity is a key aspect to be considered.

## AUTHOR CONTRIBUTIONS

Florian Altermatt and Emanuel A. Fronhofer designed the research. Florian Altermatt, Emanuel A. Fronhofer, Roman Alther performed coding and planning of molecular work. Emanuel A. Fronhofer and Roman Alther analysed data. Roman Alther and Florian Altermatt wrote the first draft of the manuscript. All authors commented on the final draft. All authors contributed to revisions.

## Supporting information

Supplementary Material

## Data Availability

The data are deposited on GitHub under the following digital object identifier 10.5281/zenodo.4321239: Microsatellite data (Gammarus_all_microsat_data_Rhein_2018.txt); Graph object (Gfos_Network.RData); Simulation code (pop_gen_gammaridae_v0.cpp); Analysis script for R (PopGenNet20210915.R).
