# Supplementary material for: Dispersal behaviour and riverine network connectivity shape the genetic diversity of freshwater amphipod metapopulations
Source: Mol Ecol. 2021 Oct 10;30(24):6551–65. doi: 10.1111/mec.16201 (PMC9293088; doi:10.1111/mec.16201)
Supplement: Supplementary file 1 — Supplementary Material [file MEC-30-6551-s001.docx]

***Supporting Information:*
Dispersal behaviour and riverine network connectivity shape the genetic diversity of freshwater amphipod metapopulations**

Roman Alther^1,2^, Emanuel A. Fronhofer^1,2,3^ & Florian Altermatt^1,2^

^1^ Eawag, Swiss Federal Institute of Aquatic Science and Technology, Department of Aquatic Ecology, Überlandstrasse 133, CH-8600 Dübendorf, Switzerland.

^2^ University of Zurich, Department of Evolutionary Biology and Environmental Studies, Winterthurerstr. 190, CH-8057 Zürich, Switzerland.

^3^ ISEM, Université de Montpellier, CNRS, IRD, EPHE, Montpellier, France.

*corresponding authors: Roman.Alther@eawag.ch and Florian.Altermatt@eawag.ch

**Number of words:** 2942

**Number of figures:** 36

**Number of tables:** 16


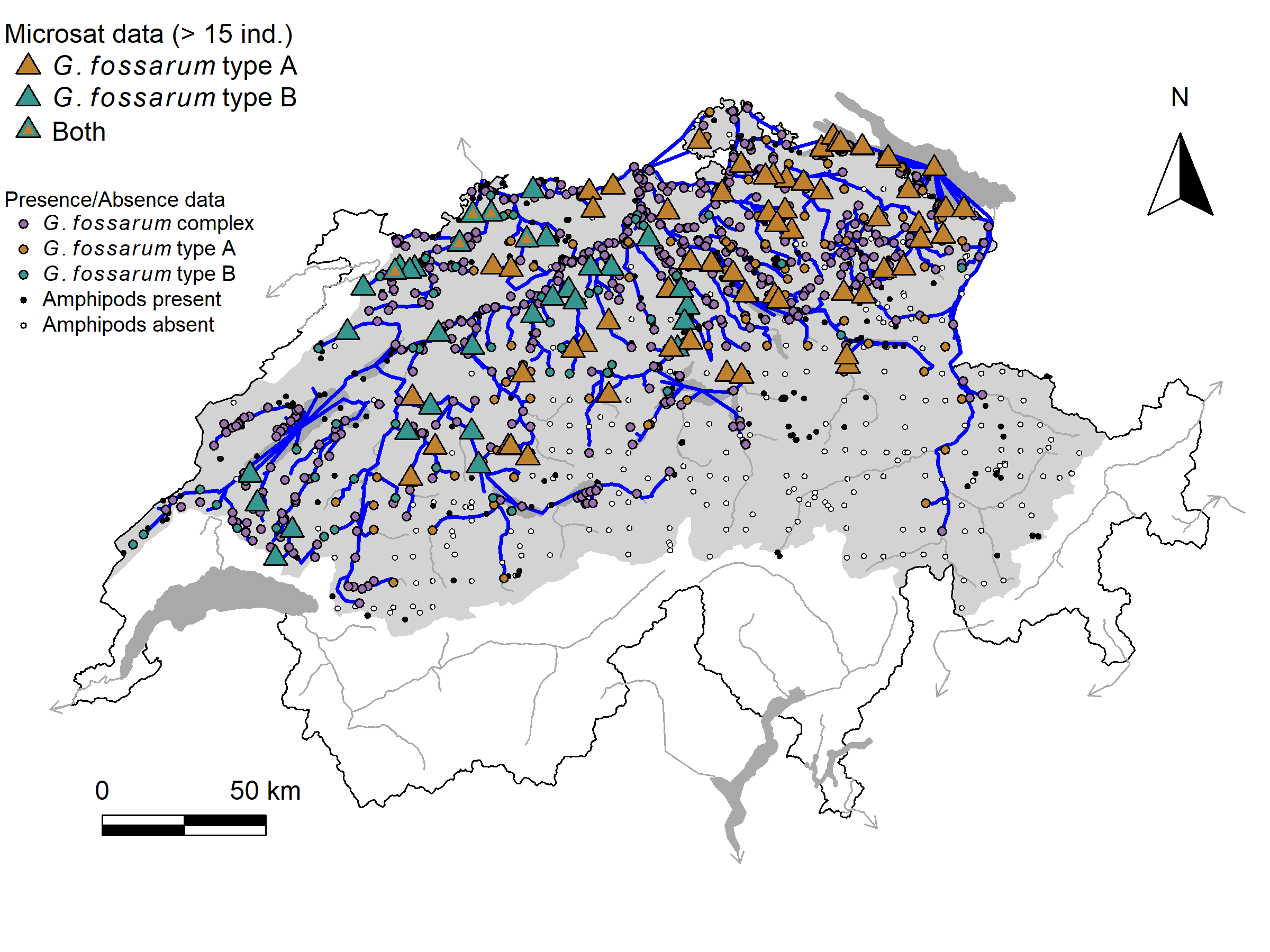


**Figure S1** Graph representation of the riverine network (blue lines) that is accessible to *Gammarus fossarum* type A and type B within the total Rhine catchment (shading). It is based on a 2 km^2^ subcatchment representation of streams and rivers of Switzerland. Geodata source: Federal Office of Topography & Federal Office for the Environment


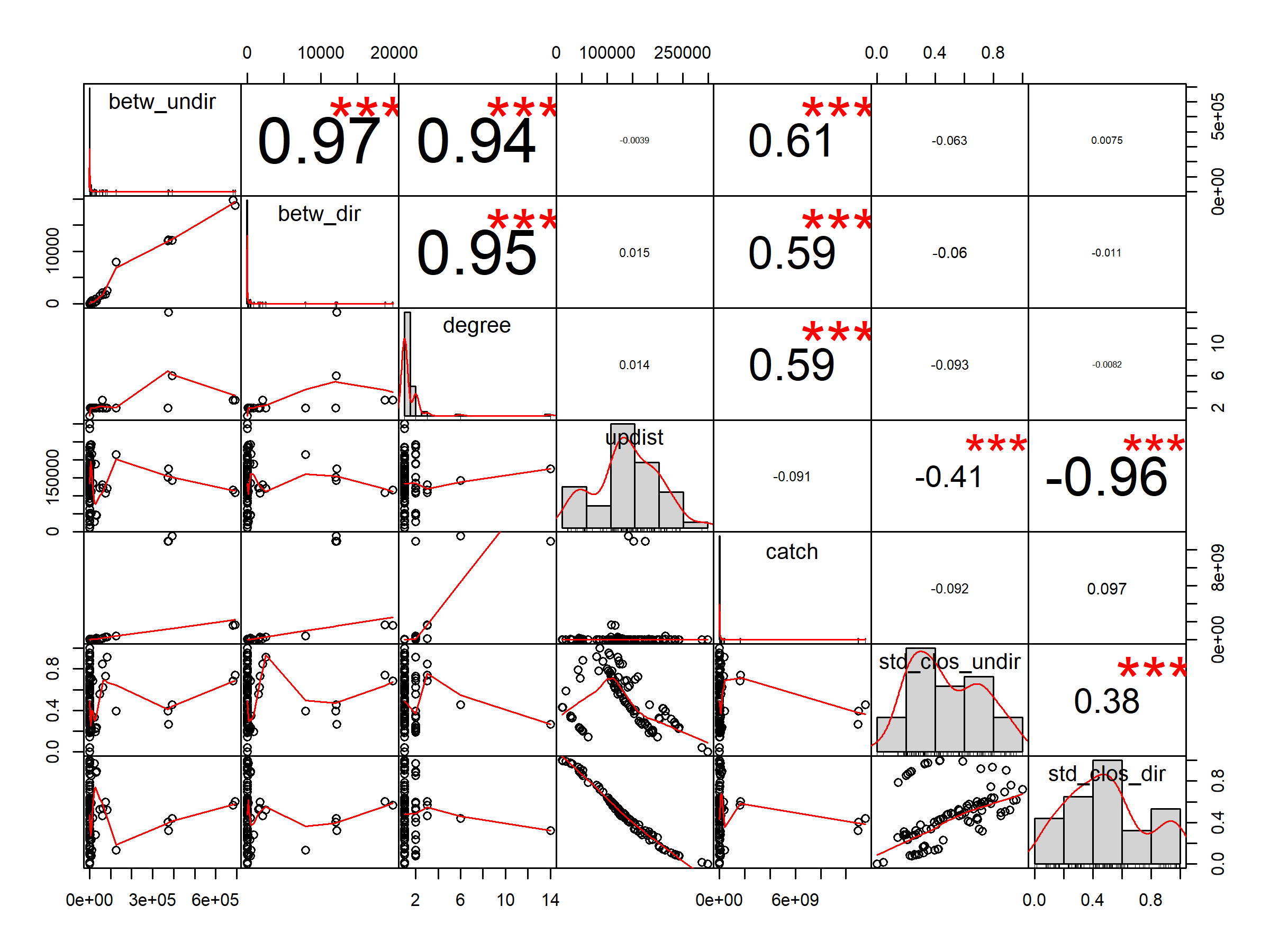


**Figure S2** Correlation plot with the distribution of each explanatory variable on the diagonal, the bivariate scatter plots with a fitted line in the bottom triangular, and the value of the correlation plus the significance level as stars in the upper triangular. Highly correlated explanatory variables (Kendall’s Tau > 0.8) were excluded, specifically undirected betweenness centrality, degree centrality, and directed closeness centrality (see Figure S3).
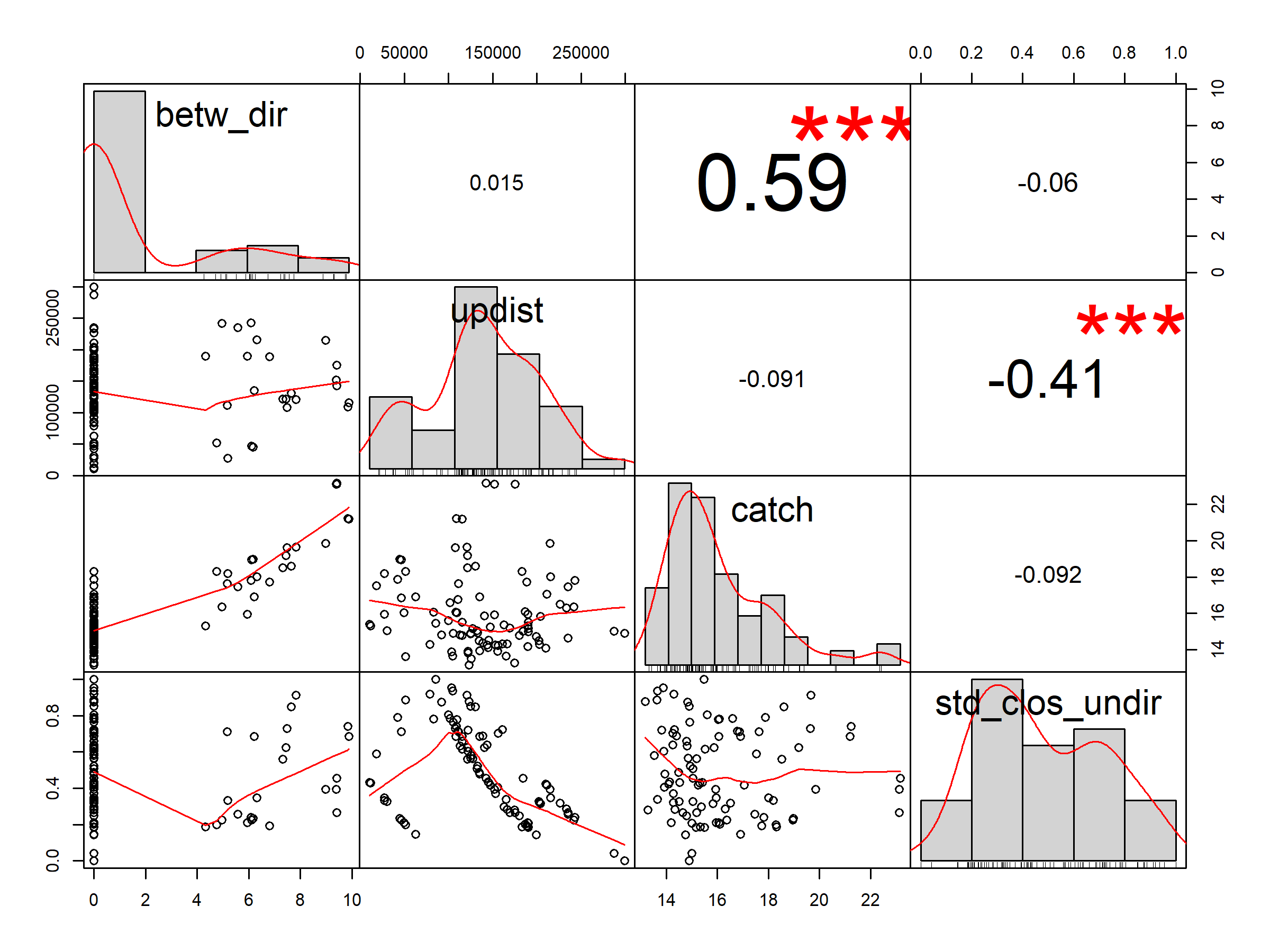


**Figure S3** Correlation plot with the distribution of the selected and transformed explanatory variables on the diagonal, the bivariate scatter plots with a fitted line in the bottom triangular, and the value of the correlation plus the significance level as stars in the upper triangular.
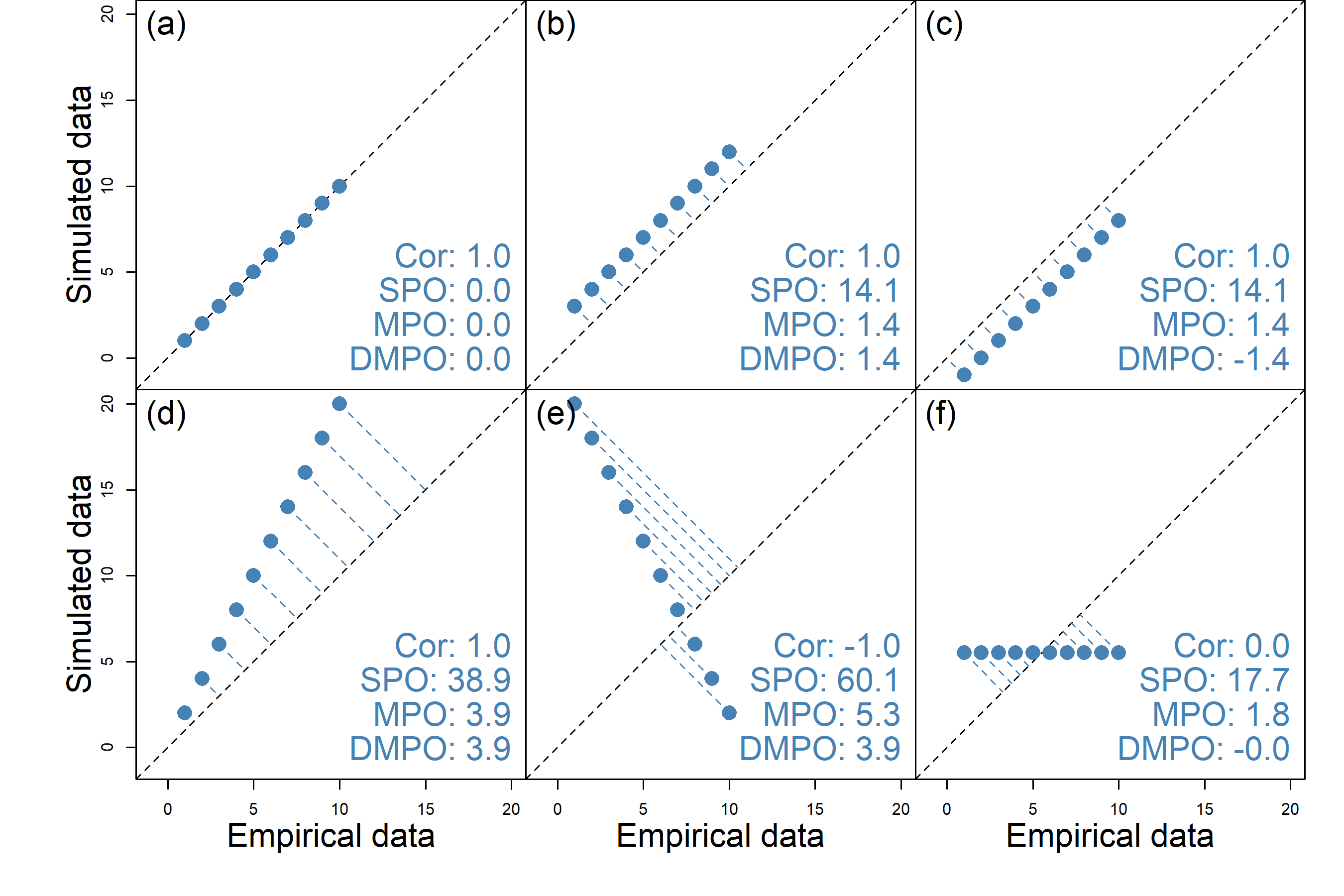


**Figure S4** Comparing simulated data and empirical data using the perpendicular offset (distance; dashed lines). The figures report the correlation coefficient, the sum of perpendicular offsets (SPO), the median of the perpendicular offsets (MPO), and the directed median of the perpendicular offset (DMPO). (a) If simulation data and empirical data were identical, they would lie on the 1:1 diagonal line. (b,c,d) There are other situations resulting in a perfect correlation, but with the simulations consequently underestimating values, overestimating values or even deviating increasingly with higher empirical values. (e) A situation with a negative correlation coefficient is also picked up using the perpendicular offset. (f) If simulated data are uncorrelated to the empirical data, they might still lie in the range or not, which is also picked up by the perpendicular offset.


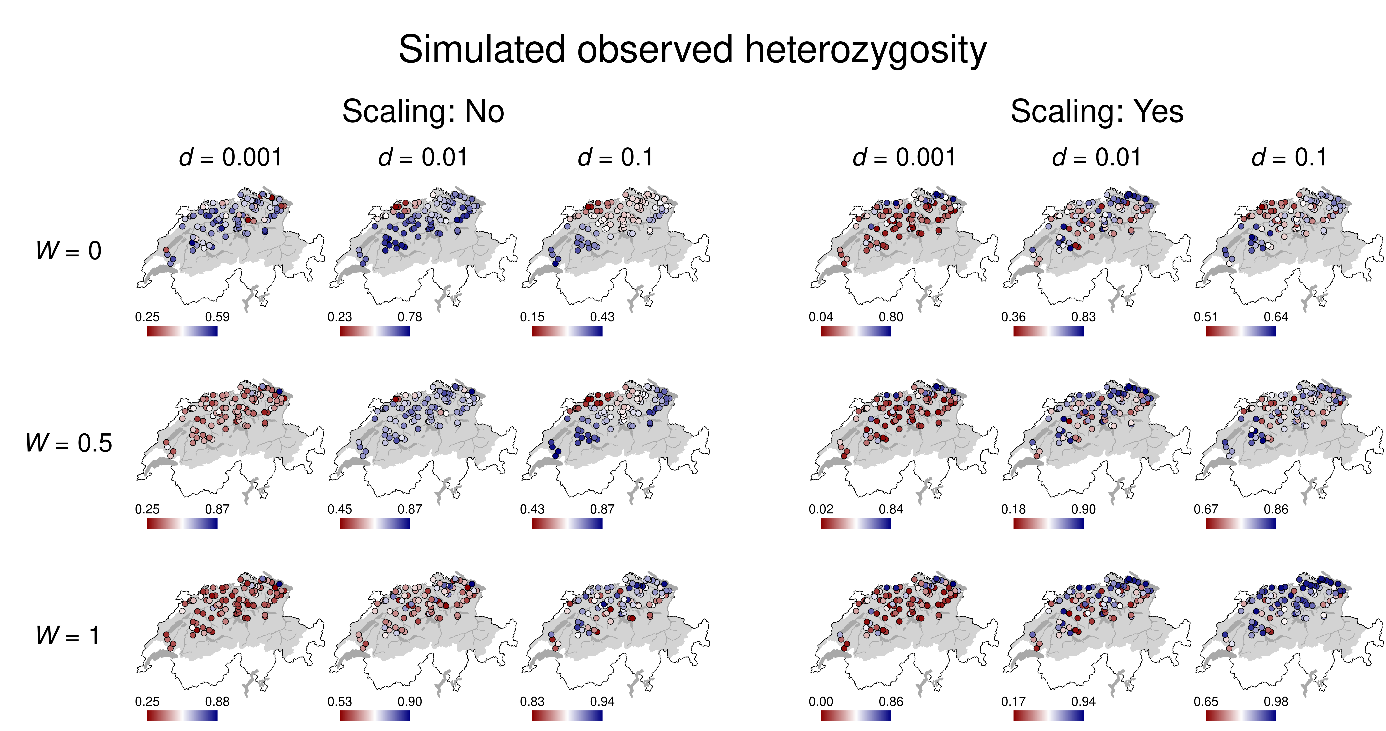


**Figure S5** Maps depicting the predicted mean observed heterozygosity for all 18 stochastic simulation scenarios show different spatial structuring along the Rhine riverine network of Switzerland. The gradient legends show mean observed heterozygosity. Their ranges are adjusted for each map for the best visual representation of spatial structuring. Geodata source: Federal Office of Topography & Federal Office for the Environment
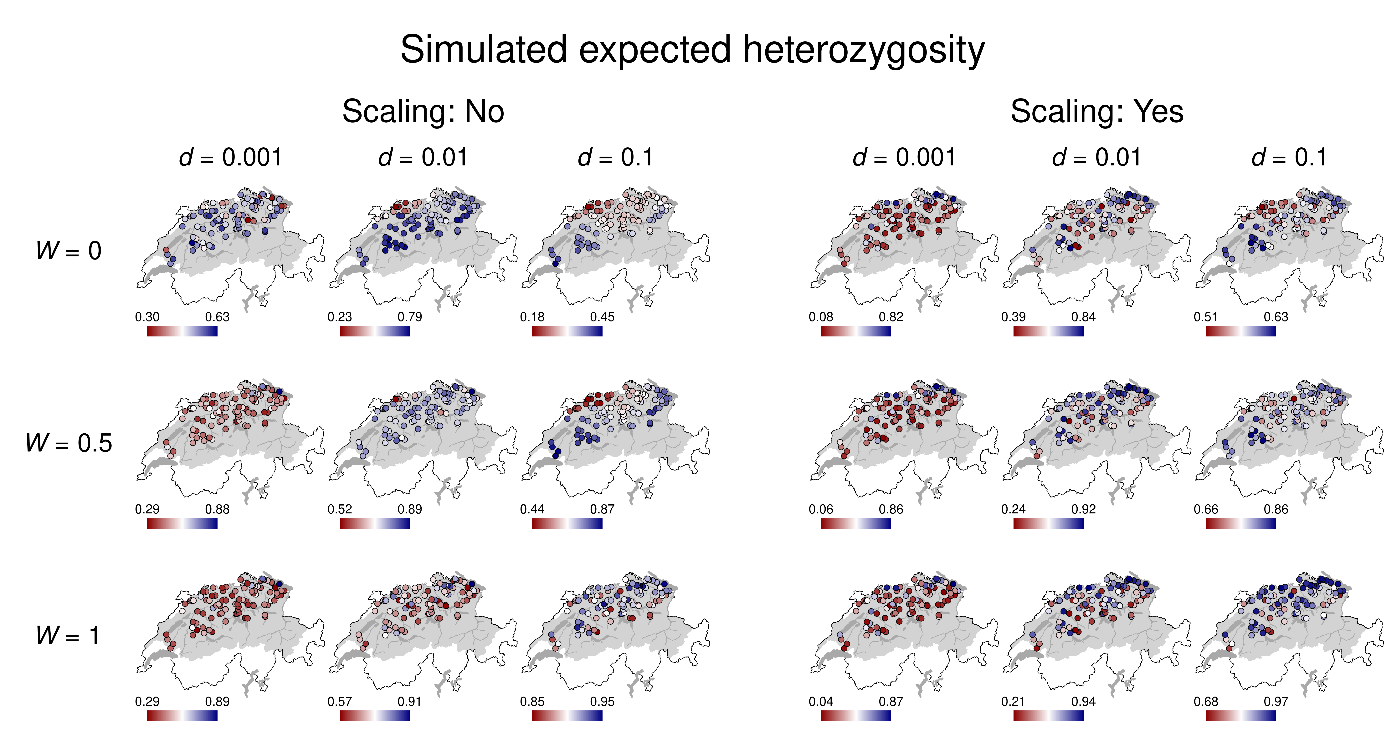


**Figure S6** Maps depicting the predicted expected heterozygosity for all 18 stochastic simulation scenarios show different spatial structuring along the Rhine riverine network of Switzerland. The gradient legends show expected heterozygosity. Their ranges are adjusted for each map for the best visual representation of spatial structuring. Geodata source: Federal Office of Topography & Federal Office for the Environment


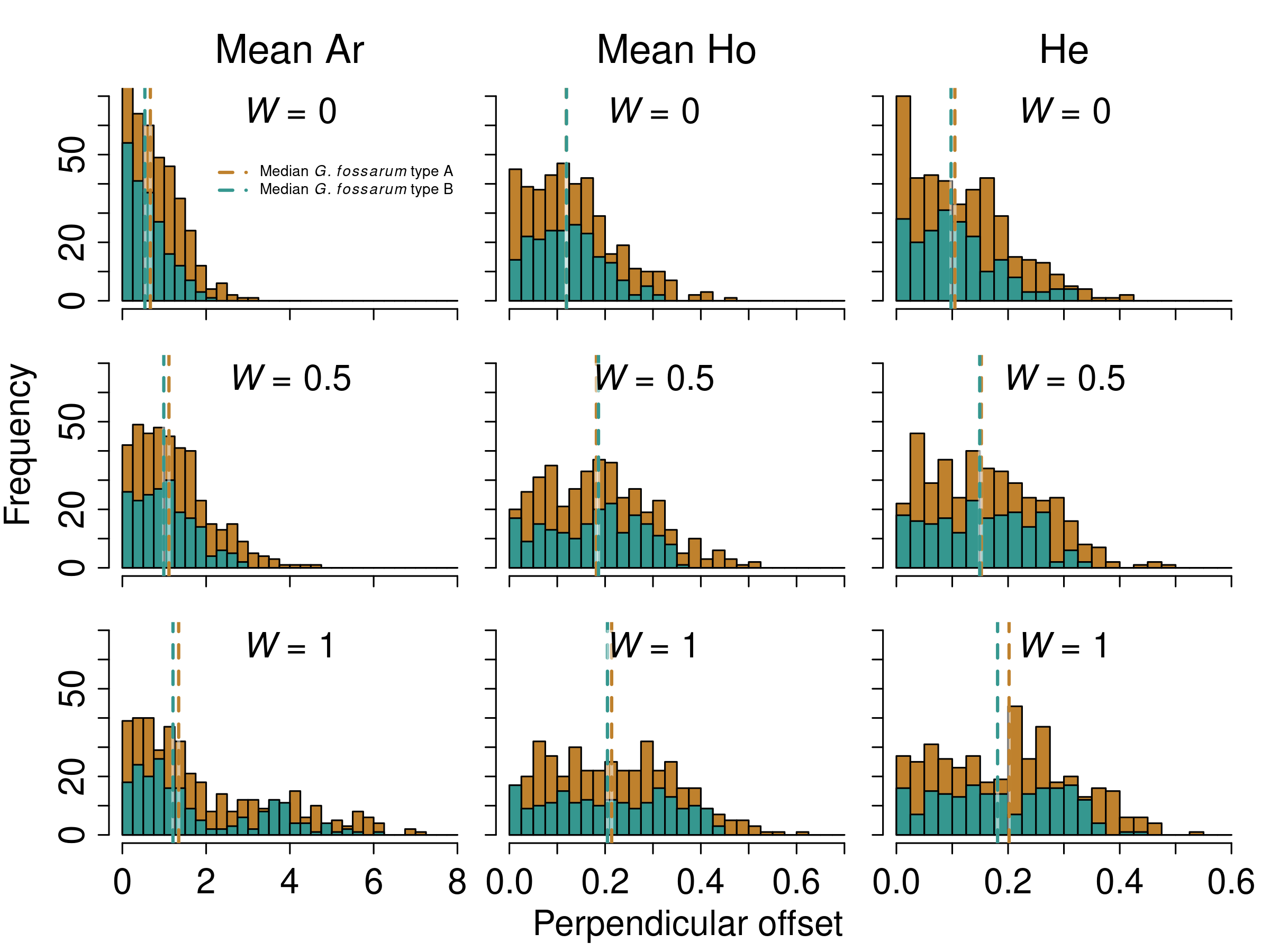


**Figure S7** Changing upstream movement probabilities in the simulations resulted in clear shifts of model fits to empirical data. The clearest signal results when allowing for upstream dispersal (*W* = 0.5 and *W* = 1), weakening model fits as reflected in perpendicular offset differences. Simulations with no upstream dispersal (*W* = 0) were superior to those simulations.


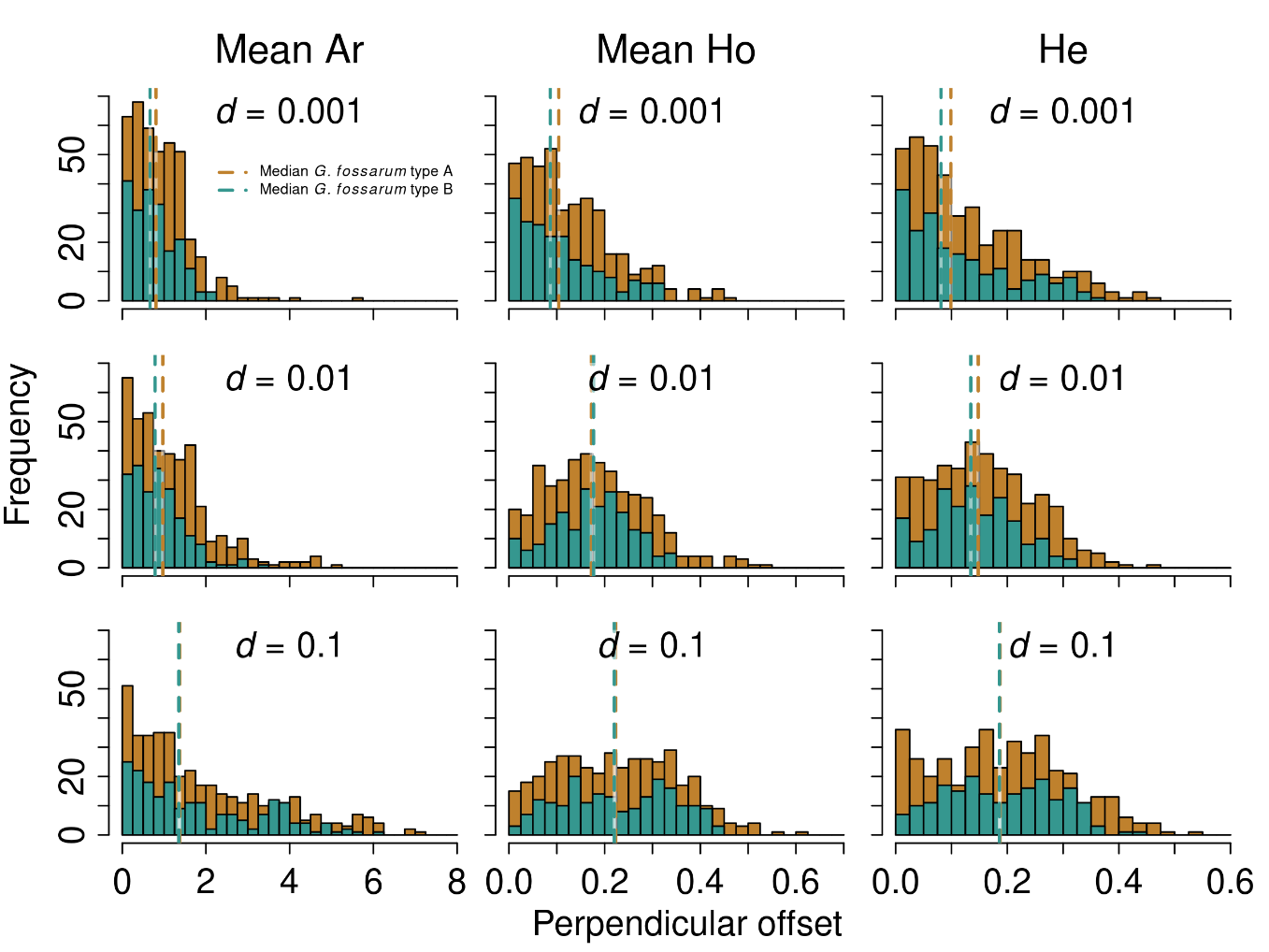


**Figure S8** Different dispersal rates in the simulations resulted in comparable variability on the simulation fit as changing the upstream movement probability (see Figure S7), reflected in perpendicular offset differences. Simulations with low dispersal rates (*d* = 0.001) were superior to simulations with higher dispersal rates. High dispersal rates generally resulted in the worst model fits.


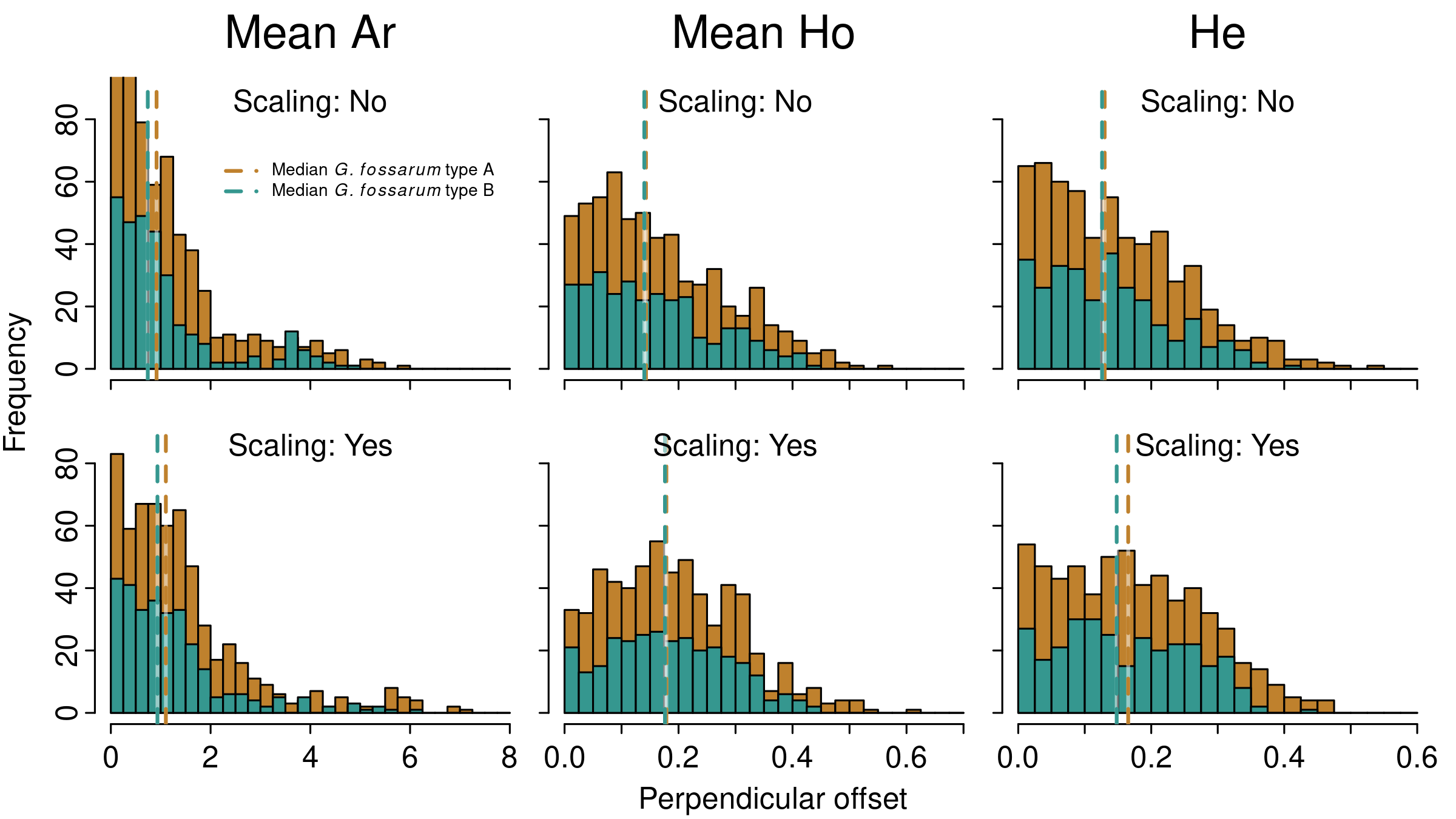


**Figure S9** Scaling the habitat carrying capacity showed the lowest impact on the variability of the response variable. Generally, simulations without scaling of the carrying capacity (*K* = 0) outperformed the ones where carrying capacity scaled with the square-root of the total catchment area.


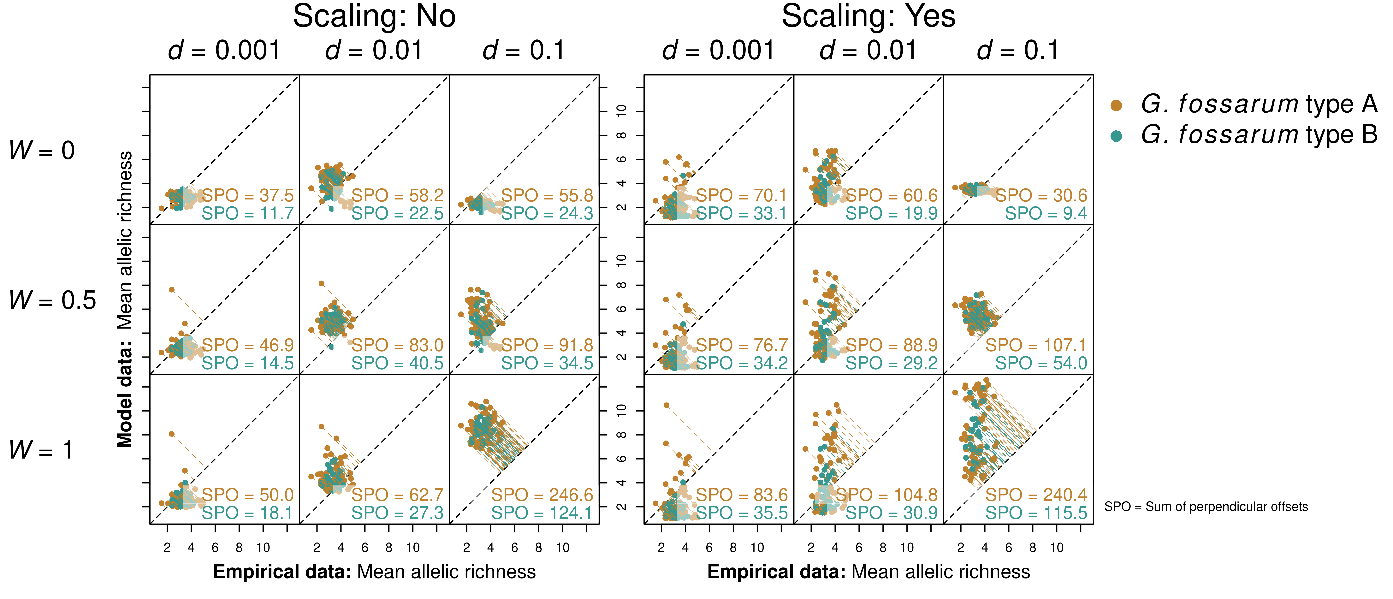


**Figure S10** Perpendicular offsets between mean allelic richness value pairs of all 18 stochastic simulation scenarios and the empirical data. The sum of the perpendicular offsets (SPO) served as a goodness-of-fit measure. SPO takes into account the overall spread of simulated values from their empirical counterpart, where larger SPO indicates a poorer fit.**Table S11** Ranking of the 18 stochastic simulations for their fit to the empirically assessed mean allelic richness values according to their sum of perpendicular offsets (SPO). Listed are the value for SPO and the corresponding varying parameter values (dispersal rate *d*, upstream movement probability *W*, and scaling of carrying capacity *K*).

| Rank | Mean allelic richness | | | | | | | |
| --- | --- | --- | --- | --- | --- | --- | --- | --- |
|  | *G. fossarum* type A | | | | *G. fossarum* type B | | | |
|  | SPO | *d* | *W* | *K* | SPO | *d* | *W* | *K* |
| 1 | 30.61 | 0.1 | 0 | 1 | 9.44 | 0.1 | 0 | 1 |
| 2 | 37.46 | 0.001 | 0 | 0 | 11.67 | 0.001 | 0 | 0 |
| 3 | 46.89 | 0.001 | 0.5 | 0 | 14.46 | 0.001 | 0.5 | 0 |
| 4 | 50.03 | 0.001 | 1 | 0 | 18.13 | 0.001 | 1 | 0 |
| 5 | 55.81 | 0.1 | 0 | 0 | 19.90 | 0.01 | 0 | 1 |
| 6 | 58.16 | 0.01 | 0 | 0 | 22.55 | 0.01 | 0 | 0 |
| 7 | 60.63 | 0.01 | 0 | 1 | 24.34 | 0.1 | 0 | 0 |
| 8 | 62.70 | 0.01 | 1 | 0 | 27.30 | 0.01 | 1 | 0 |
| 9 | 70.12 | 0.001 | 0 | 1 | 29.19 | 0.01 | 0.5 | 1 |
| 10 | 76.69 | 0.001 | 0.5 | 1 | 30.85 | 0.01 | 1 | 1 |
| 11 | 83.01 | 0.01 | 0.5 | 0 | 33.15 | 0.001 | 0 | 1 |
| 12 | 83.62 | 0.001 | 1 | 1 | 34.19 | 0.001 | 0.5 | 1 |
| 13 | 88.85 | 0.01 | 0.5 | 1 | 34.47 | 0.1 | 0.5 | 0 |
| 14 | 91.78 | 0.1 | 0.5 | 0 | 35.48 | 0.001 | 1 | 1 |
| 15 | 104.83 | 0.01 | 1 | 1 | 40.52 | 0.01 | 0.5 | 0 |
| 16 | 107.08 | 0.1 | 0.5 | 1 | 54.04 | 0.1 | 0.5 | 1 |
| 17 | 240.37 | 0.1 | 1 | 1 | 115.53 | 0.1 | 1 | 1 |
| 18 | 246.61 | 0.1 | 1 | 0 | 124.09 | 0.1 | 1 | 0 |


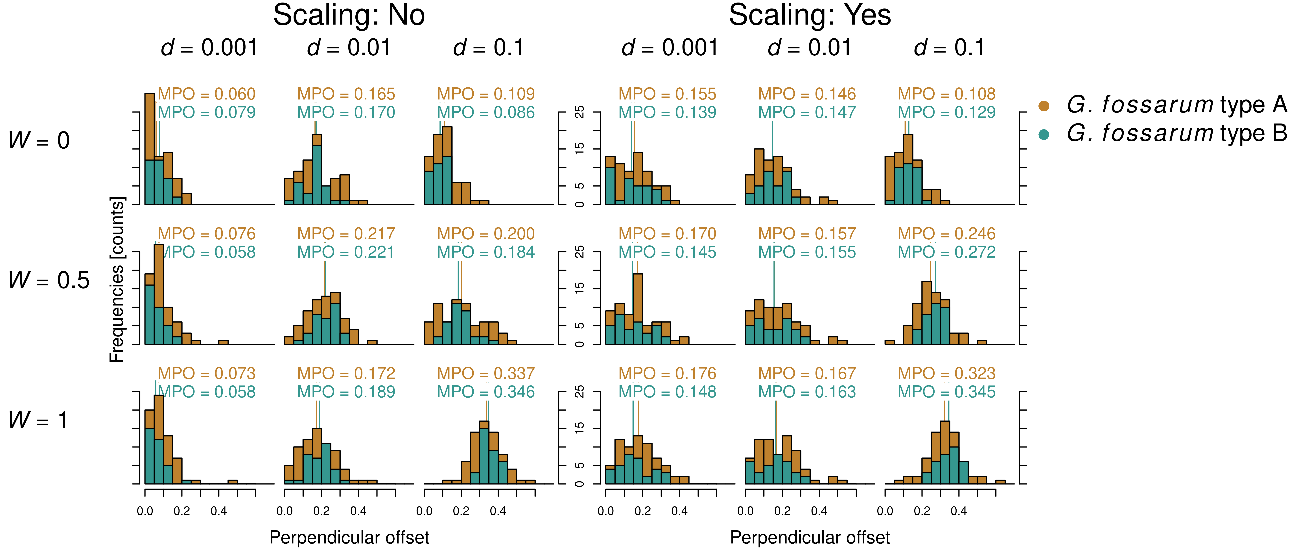


**Figure S12** Histograms and medians of the perpendicular offsets (MPO) between all 18 stochastic simulation scenarios and the empirical mean observed heterozygosity values for both species of the *Gammarus fossarum* complex (type A: orange symbols, type B: cyan symbols). The actual perpendicular offsets of all 18 scenarios is given in Figure S13.
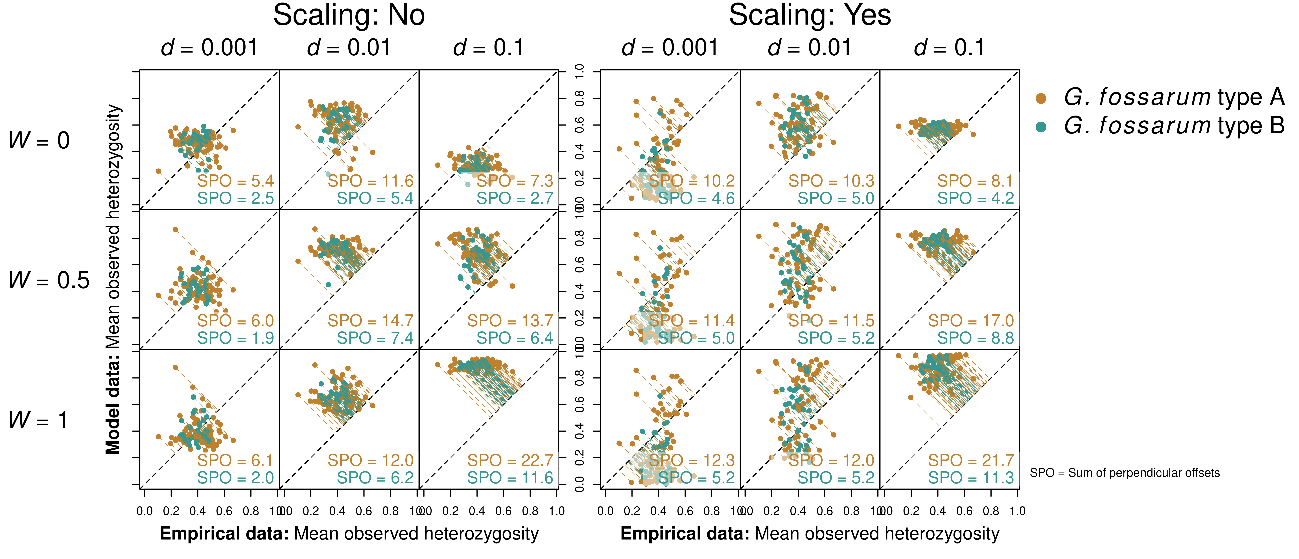


**Figure S13** Perpendicular offsets between mean observed heterozygosity value pairs of all 18 stochastic simulation scenarios and the empirical data. The sum of the perpendicular offsets (SPO) served as a goodness-of-fit measure. SPO takes into account the overall spread of simulated values from their empirical counterpart, where larger SPO indicates a poorer fit.

| Rank | Mean observed heterozygosity | | | | | | | |
| --- | --- | --- | --- | --- | --- | --- | --- | --- |
|  | *G. fossarum* type A | | | | *G. fossarum* type B | | | |
|  | SPO | *d* | *W* | *K* | SPO | *d* | *W* | *K* |
| 1 | 5.36 | 0.001 | 0 | 0 | 1.92 | 0.001 | 0.5 | 0 |
| 2 | 6.00 | 0.001 | 0.5 | 0 | 1.98 | 0.001 | 1 | 0 |
| 3 | 6.09 | 0.001 | 1 | 0 | 2.52 | 0.001 | 0 | 0 |
| 4 | 7.31 | 0.1 | 0 | 0 | 2.68 | 0.1 | 0 | 0 |
| 5 | 8.15 | 0.1 | 0 | 1 | 4.17 | 0.1 | 0 | 1 |
| 6 | 10.22 | 0.001 | 0 | 1 | 4.56 | 0.001 | 0 | 1 |
| 7 | 10.35 | 0.01 | 0 | 1 | 4.96 | 0.001 | 0.5 | 1 |
| 8 | 11.39 | 0.001 | 0.5 | 1 | 4.98 | 0.01 | 0 | 1 |
| 9 | 11.48 | 0.01 | 0.5 | 1 | 5.15 | 0.01 | 1 | 1 |
| 10 | 11.61 | 0.01 | 0 | 0 | 5.17 | 0.001 | 1 | 1 |
| 11 | 12.02 | 0.01 | 1 | 1 | 5.18 | 0.01 | 0.5 | 1 |
| 12 | 12.05 | 0.01 | 1 | 0 | 5.41 | 0.01 | 0 | 0 |
| 13 | 12.29 | 0.001 | 1 | 1 | 6.17 | 0.01 | 1 | 0 |
| 14 | 13.71 | 0.1 | 0.5 | 0 | 6.38 | 0.1 | 0.5 | 0 |
| 15 | 14.70 | 0.01 | 0.5 | 0 | 7.44 | 0.01 | 0.5 | 0 |
| 16 | 17.04 | 0.1 | 0.5 | 1 | 8.81 | 0.1 | 0.5 | 1 |
| 17 | 21.74 | 0.1 | 1 | 1 | 11.29 | 0.1 | 1 | 1 |
| 18 | 22.71 | 0.1 | 1 | 0 | 11.58 | 0.1 | 1 | 0 |

**Table S14** Ranking of the 18 stochastic simulations for their fit to the empirically assessed mean observed heterozygosity values according to their sum of perpendicular offsets (SPO). Listed are the value for SPO and the corresponding varying parameter values (dispersal rate *d*, upstream movement probability *W*, and scaling of carrying capacity *K*).
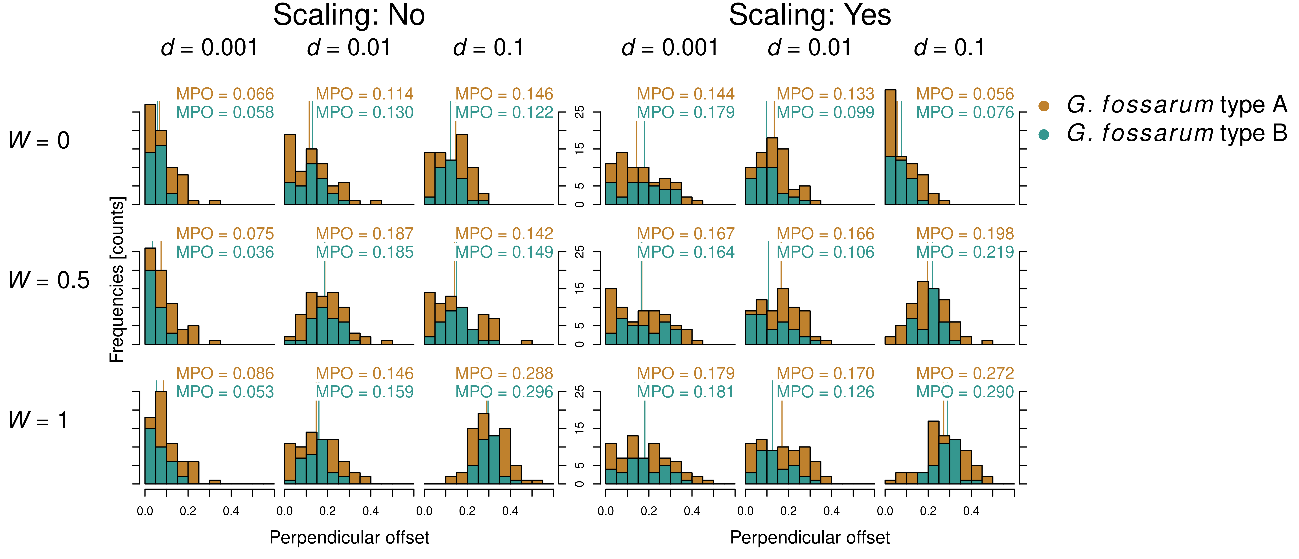


**Figure S15** Histograms and medians of the perpendicular offsets (MPO) between all 18 stochastic simulation scenarios and the empirical expected heterozygosity values for both species of the *Gammarus fossarum* complex (type A: orange symbols, type B: cyan symbols). The actual perpendicular offsets of all 18 scenarios is given in Figure S16.
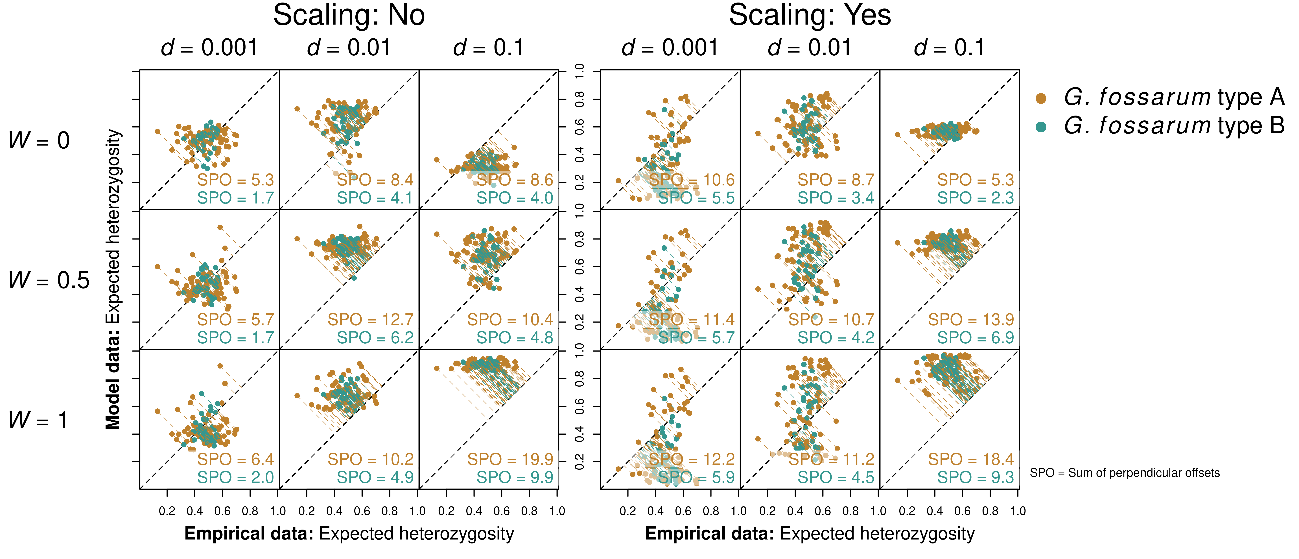


**Figure S16** Perpendicular offsets between expected heterozygosity value pairs of all 18 stochastic simulation scenarios and the empirical data. The sum of the perpendicular offsets (SPO) served as a goodness-of-fit measure. SPO takes into account the overall spread of simulated values from their empirical counterpart, where larger SPO indicates a poorer fit.

| Rank | Expected heterozygosity | | | | | | | |
| --- | --- | --- | --- | --- | --- | --- | --- | --- |
|  | *G. fossarum* type A | | | | *G. fossarum* type B | | | |
|  | SPO | *d* | *W* | *K* | SPO | *d* | *W* | *K* |
| 1 | 5.27 | 0.001 | 0 | 0 | 1.68 | 0.001 | 0.5 | 0 |
| 2 | 5.35 | 0.1 | 0 | 1 | 1.73 | 0.001 | 0 | 0 |
| 3 | 5.66 | 0.001 | 0.5 | 0 | 2.03 | 0.001 | 1 | 0 |
| 4 | 6.41 | 0.001 | 1 | 0 | 2.29 | 0.1 | 0 | 1 |
| 5 | 8.45 | 0.01 | 0 | 0 | 3.36 | 0.01 | 0 | 1 |
| 6 | 8.60 | 0.1 | 0 | 0 | 4.04 | 0.1 | 0 | 0 |
| 7 | 8.69 | 0.01 | 0 | 1 | 4.05 | 0.01 | 0 | 0 |
| 8 | 10.18 | 0.01 | 1 | 0 | 4.19 | 0.01 | 0.5 | 1 |
| 9 | 10.36 | 0.1 | 0.5 | 0 | 4.54 | 0.01 | 1 | 1 |
| 10 | 10.64 | 0.001 | 0 | 1 | 4.80 | 0.1 | 0.5 | 0 |
| 11 | 10.71 | 0.01 | 0.5 | 1 | 4.93 | 0.01 | 1 | 0 |
| 12 | 11.21 | 0.01 | 1 | 1 | 5.51 | 0.001 | 0 | 1 |
| 13 | 11.38 | 0.001 | 0.5 | 1 | 5.67 | 0.001 | 0.5 | 1 |
| 14 | 12.22 | 0.001 | 1 | 1 | 5.87 | 0.001 | 1 | 1 |
| 15 | 12.74 | 0.01 | 0.5 | 0 | 6.21 | 0.01 | 0.5 | 0 |
| 16 | 13.89 | 0.1 | 0.5 | 1 | 6.91 | 0.1 | 0.5 | 1 |
| 17 | 18.43 | 0.1 | 1 | 1 | 9.35 | 0.1 | 1 | 1 |
| 18 | 19.86 | 0.1 | 1 | 0 | 9.88 | 0.1 | 1 | 0 |

**Table S17** Ranking of the 18 stochastic simulations for their fit to the empirically assessed expected heterozygosity values according to their sum of perpendicular offsets (SPO). Listed are the value for SPO and the corresponding varying parameter values (dispersal rate *d*, upstream movement probability *W*, and scaling of carrying capacity *K*).

| Rank | Mean allelic richness | | | | | | | |
| --- | --- | --- | --- | --- | --- | --- | --- | --- |
|  | *G. fossarum* type A | | | | *G. fossarum* type B | | | |
|  | MPO | *d* | *W* | *K* | MPO | *d* | *W* | *K* |
| 1 | 0.352 | 0.1 | 0 | 1 | 0.240 | 0.001 | 0 | 0 |
| 2 | 0.473 | 0.001 | 0 | 0 | 0.261 | 0.1 | 0 | 1 |
| 3 | 0.589 | 0.001 | 0.5 | 0 | 0.339 | 0.001 | 0.5 | 0 |
| 4 | 0.600 | 0.001 | 1 | 0 | 0.462 | 0.01 | 0 | 1 |
| 5 | 0.738 | 0.01 | 1 | 0 | 0.564 | 0.001 | 1 | 0 |
| 6 | 0.740 | 0.01 | 0 | 1 | 0.632 | 0.01 | 0 | 0 |
| 7 | 0.794 | 0.1 | 0 | 0 | 0.748 | 0.1 | 0 | 0 |
| 8 | 0.842 | 0.01 | 0 | 0 | 0.767 | 0.01 | 1 | 0 |
| 9 | 0.980 | 0.01 | 0.5 | 1 | 0.784 | 0.01 | 0.5 | 1 |
| 10 | 1.066 | 0.001 | 0 | 1 | 0.822 | 0.01 | 1 | 1 |
| 11 | 1.092 | 0.01 | 1 | 1 | 0.931 | 0.1 | 0.5 | 0 |
| 12 | 1.117 | 0.001 | 0.5 | 1 | 1.050 | 0.001 | 0 | 1 |
| 13 | 1.157 | 0.1 | 0.5 | 0 | 1.084 | 0.001 | 0.5 | 1 |
| 14 | 1.225 | 0.001 | 1 | 1 | 1.188 | 0.001 | 1 | 1 |
| 15 | 1.288 | 0.01 | 0.5 | 0 | 1.220 | 0.01 | 0.5 | 0 |
| 16 | 1.578 | 0.1 | 0.5 | 1 | 1.592 | 0.1 | 0.5 | 1 |
| 17 | 3.640 | 0.1 | 1 | 0 | 3.373 | 0.1 | 1 | 1 |
| 18 | 3.701 | 0.1 | 1 | 1 | 3.688 | 0.1 | 1 | 0 |

**Table S18** Ranking of the 18 stochastic simulations for their fit to the empirically assessed mean allelic richness values according to their median of perpendicular offsets (MPO). Listed are the value for MPO and the corresponding varying parameter values (dispersal rate *d*, upstream movement probability *W*, and scaling of carrying capacity *K*).

| Rank | Mean observed heterozygosity | | | | | | | |
| --- | --- | --- | --- | --- | --- | --- | --- | --- |
|  | *G. fossarum* type A | | | | *G. fossarum* type B | | | |
|  | MPO | *d* | *W* | *K* | MPO | *d* | *W* | *K* |
| 1 | 0.060 | 0.001 | 0 | 0 | 0.058 | 0.001 | 0.5 | 0 |
| 2 | 0.073 | 0.001 | 1 | 0 | 0.058 | 0.001 | 1 | 0 |
| 3 | 0.076 | 0.001 | 0.5 | 0 | 0.079 | 0.001 | 0 | 0 |
| 4 | 0.108 | 0.1 | 0 | 1 | 0.086 | 0.1 | 0 | 0 |
| 5 | 0.109 | 0.1 | 0 | 0 | 0.129 | 0.1 | 0 | 1 |
| 6 | 0.146 | 0.01 | 0 | 1 | 0.139 | 0.001 | 0 | 1 |
| 7 | 0.155 | 0.001 | 0 | 1 | 0.145 | 0.001 | 0.5 | 1 |
| 8 | 0.157 | 0.01 | 0.5 | 1 | 0.147 | 0.01 | 0 | 1 |
| 9 | 0.165 | 0.01 | 0 | 0 | 0.148 | 0.001 | 1 | 1 |
| 10 | 0.167 | 0.01 | 1 | 1 | 0.155 | 0.01 | 0.5 | 1 |
| 11 | 0.170 | 0.001 | 0.5 | 1 | 0.163 | 0.01 | 1 | 1 |
| 12 | 0.172 | 0.01 | 1 | 0 | 0.170 | 0.01 | 0 | 0 |
| 13 | 0.176 | 0.001 | 1 | 1 | 0.184 | 0.1 | 0.5 | 0 |
| 14 | 0.200 | 0.1 | 0.5 | 0 | 0.189 | 0.01 | 1 | 0 |
| 15 | 0.217 | 0.01 | 0.5 | 0 | 0.221 | 0.01 | 0.5 | 0 |
| 16 | 0.246 | 0.1 | 0.5 | 1 | 0.272 | 0.1 | 0.5 | 1 |
| 17 | 0.323 | 0.1 | 1 | 1 | 0.345 | 0.1 | 1 | 1 |
| 18 | 0.337 | 0.1 | 1 | 0 | 0.346 | 0.1 | 1 | 0 |

**Table S19** Ranking of the 18 stochastic simulations for their fit to the empirically assessed mean observed heterozygosity values according to their median of perpendicular offsets (MPO). Listed are the value for MPO and the corresponding varying parameter values (dispersal rate *d*, upstream movement probability *W*, and scaling of habitat carrying capacity *K*).

| Rank | Expected heterozygosity | | | | | | | |
| --- | --- | --- | --- | --- | --- | --- | --- | --- |
|  | *G. fossarum* type A | | | | *G. fossarum* type B | | | |
|  | MPO | *d* | *W* | *K* | MPO | *d* | *W* | *K* |
| 1 | 0.056 | 0.1 | 0 | 1 | 0.036 | 0.001 | 0.5 | 0 |
| 2 | 0.066 | 0.001 | 0 | 0 | 0.053 | 0.001 | 1 | 0 |
| 3 | 0.075 | 0.001 | 0.5 | 0 | 0.058 | 0.001 | 0 | 0 |
| 4 | 0.086 | 0.001 | 1 | 0 | 0.076 | 0.1 | 0 | 1 |
| 5 | 0.114 | 0.01 | 0 | 0 | 0.099 | 0.01 | 0 | 1 |
| 6 | 0.133 | 0.01 | 0 | 1 | 0.106 | 0.01 | 0.5 | 1 |
| 7 | 0.142 | 0.1 | 0.5 | 0 | 0.122 | 0.1 | 0 | 0 |
| 8 | 0.144 | 0.001 | 0 | 1 | 0.126 | 0.01 | 1 | 1 |
| 9 | 0.146 | 0.01 | 1 | 0 | 0.130 | 0.01 | 0 | 0 |
| 10 | 0.146 | 0.1 | 0 | 0 | 0.149 | 0.1 | 0.5 | 0 |
| 11 | 0.166 | 0.01 | 0.5 | 1 | 0.159 | 0.01 | 1 | 0 |
| 12 | 0.167 | 0.001 | 0.5 | 1 | 0.164 | 0.001 | 0.5 | 1 |
| 13 | 0.170 | 0.01 | 1 | 1 | 0.179 | 0.001 | 0 | 1 |
| 14 | 0.179 | 0.001 | 1 | 1 | 0.181 | 0.001 | 1 | 1 |
| 15 | 0.187 | 0.01 | 0.5 | 0 | 0.185 | 0.01 | 0.5 | 0 |
| 16 | 0.198 | 0.1 | 0.5 | 1 | 0.219 | 0.1 | 0.5 | 1 |
| 17 | 0.272 | 0.1 | 1 | 1 | 0.290 | 0.1 | 1 | 1 |
| 18 | 0.288 | 0.1 | 1 | 0 | 0.296 | 0.1 | 1 | 0 |

**Table S20** Ranking of the 18 stochastic simulations for their fit to the empirically assessed expected heterozygosity values according to their median of perpendicular offsets (MPO). Listed are the value for MPO and the corresponding varying parameter values (dispersal rate *d*, upstream movement probability *W*, and scaling of habitat carrying capacity *K*).


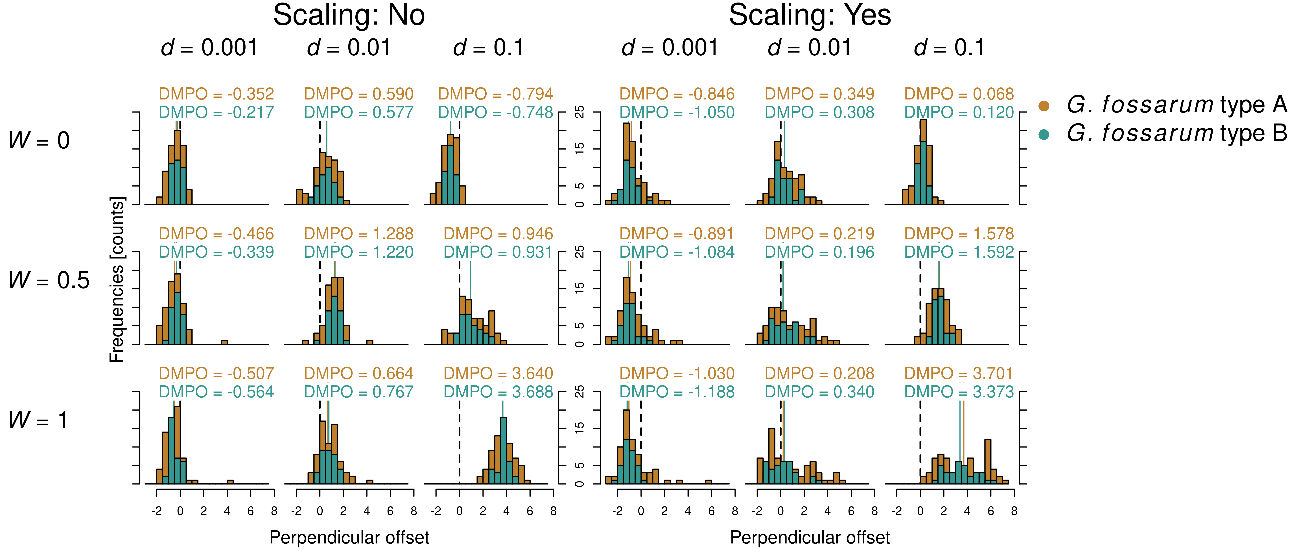
**Figure S21** Histograms and medians of the directed perpendicular offsets (DMPO) between all 18 stochastic simulation scenarios and the empirically assessed mean allelic richness values. The directed perpendicular offset does take into account if points are above or below the vertical (1:1) line. The dotted vertical line shows zero offset, corresponding to a perfect fit between simulation and empirical data.


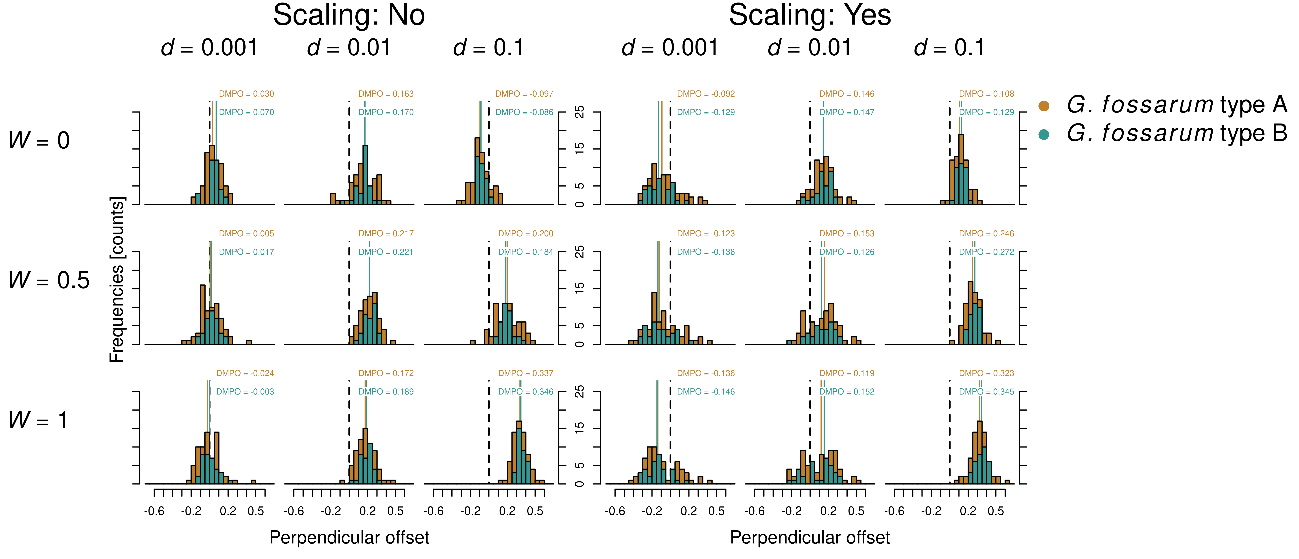


**Figure S22** Histograms and medians of the directed perpendicular offsets (DMPO) between all 18 stochastic simulation scenarios and the empirically assessed mean observed heterozygosity values. The directed perpendicular offset does take into account if points are above or below the vertical (1:1) line. The dotted vertical line shows zero offset, corresponding to a perfect fit between simulation and empirical data.
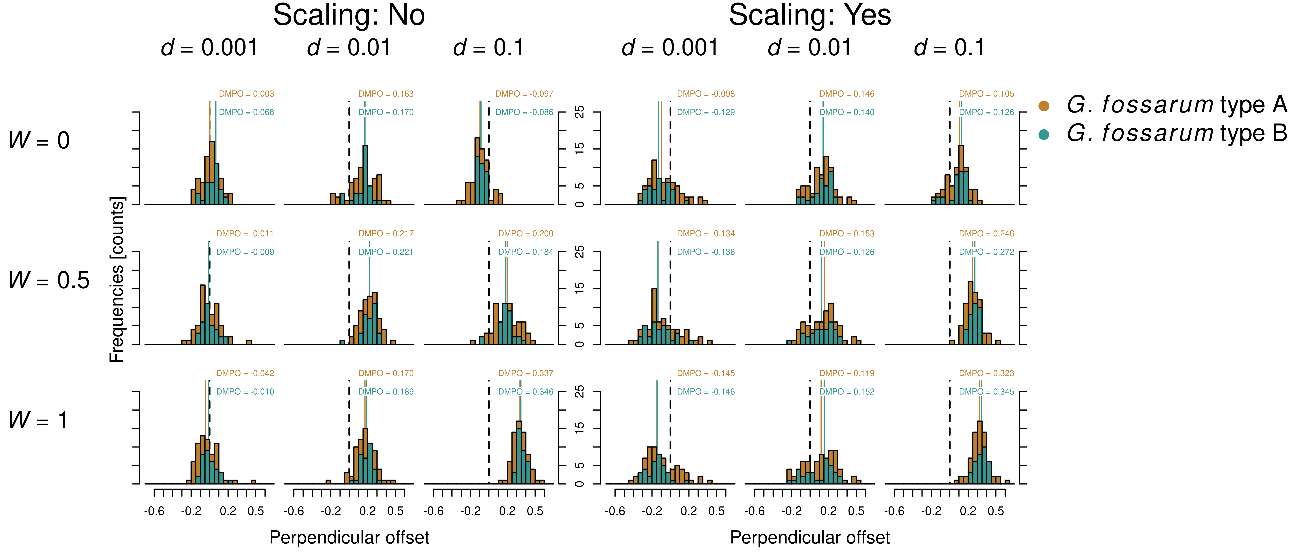


**Figure S23** Histograms and medians of the directed perpendicular offsets (DMPO) between all 18 stochastic simulation scenarios and the empirically assessed expected heterozygosity values. The directed perpendicular offset does take into account if points are above or below the vertical (1:1) line. The dotted vertical line shows zero offset, corresponding to a perfect fit between simulation and empirical data.


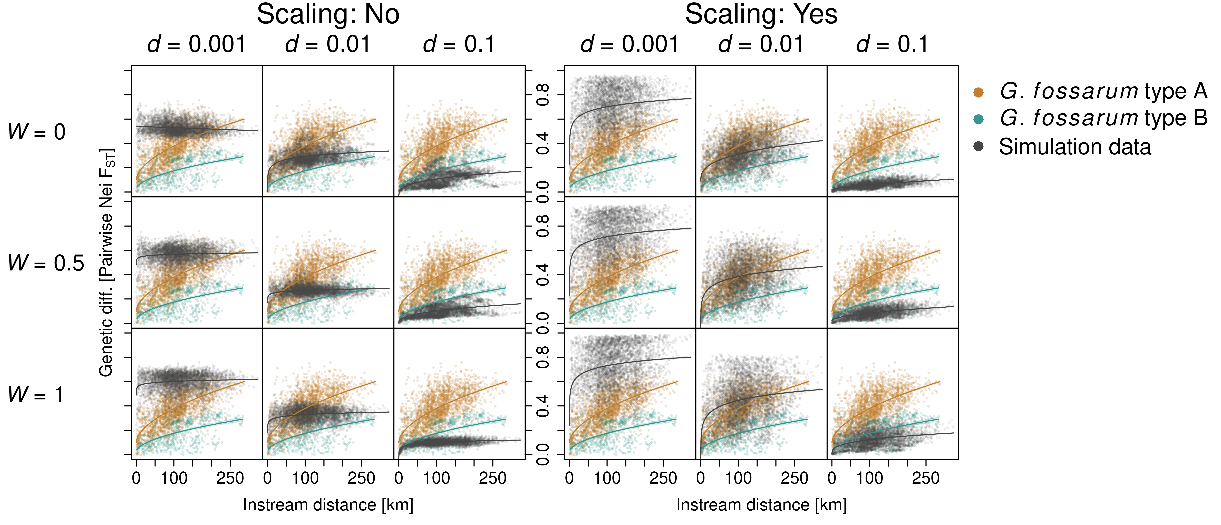


**Figure S24** Pairwise genetic differentiation F_ST_ plotted against the instream distance between nodes of the riverine network (Lines: LM with power term; shading depicts 95% confidence interval). Some simulated data matched the empirical data relatively well, others were completely off. The best-fitting simulations were based on moderate dispersal rates (d = 0.01).


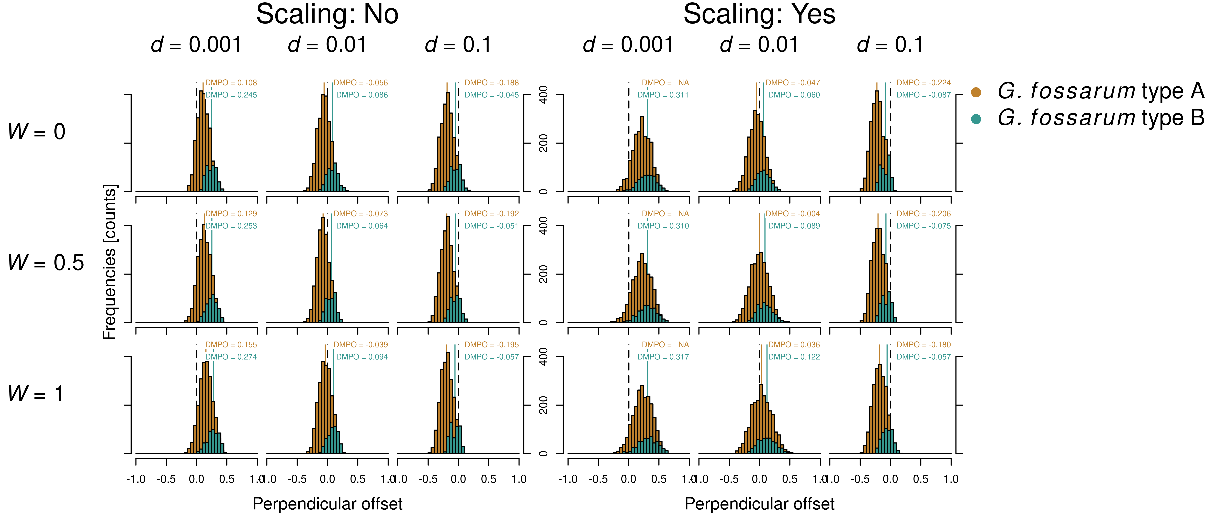


**Figure S25** Histograms and medians of the directed perpendicular offsets (DMPO) between all 18 stochastic simulation scenarios and the empirically assessed pairwise genetic differentiation (Nei F_ST_) values. The directed perpendicular offset does take into account if points are above or below the vertical (1:1) line. The dotted vertical line shows zero offset, corresponding to a perfect fit between simulation and empirical data.

**Table S26** Ranking of the 18 stochastic simulations for their fit to the empirically assessed pairwise genetic differentiation (Nei F_ST_) values according to their sum of perpendicular offsets (SPO). Listed are the value for SPO and the corresponding varying parameter values (dispersal rate *d*, upstream movement probability *W*, and scaling of carrying capacity *K*).

| Rank | Pairwise genetic differentiation | | | | | | | |
| --- | --- | --- | --- | --- | --- | --- | --- | --- |
|  | *G. fossarum* type A | | | | *G. fossarum* type B | | | |
|  | SPO | *d* | *W* | *K* | SPO | *d* | *W* | *K* |
| 1 | 203.2 | 0.01 | 1 | 0 | 41.4 | 0.1 | 1 | 0 |
| 2 | 217.4 | 0.01 | 0 | 0 | 42.0 | 0.01 | 0.5 | 0 |
| 3 | 224.3 | 0.01 | 0.5 | 0 | 42.3 | 0.1 | 0 | 0 |
| 4 | 243.8 | 0.01 | 0 | 1 | 42.3 | 0.1 | 0.5 | 0 |
| 5 | 262.9 | 0.01 | 0.5 | 1 | 43.3 | 0.1 | 1 | 1 |
| 6 | 274.3 | 0.001 | 0 | 0 | 45.9 | 0.1 | 0.5 | 1 |
| 7 | 300.5 | 0.01 | 1 | 1 | 50.3 | 0.1 | 0 | 1 |
| 8 | 316.2 | 0.001 | 0.5 | 0 | 52.2 | 0.01 | 0 | 0 |
| 9 | 361.0 | 0.001 | 1 | 0 | 53.9 | 0.01 | 1 | 0 |
| 10 | 405.1 | 0.1 | 1 | 1 | 54.6 | 0.01 | 0 | 1 |
| 11 | 420.7 | 0.1 | 0 | 0 | 66.4 | 0.01 | 0.5 | 1 |
| 12 | 427.7 | 0.1 | 0.5 | 0 | 83.0 | 0.01 | 1 | 1 |
| 13 | 432.0 | 0.1 | 1 | 0 | 129.3 | 0.001 | 0 | 0 |
| 14 | 452.8 | 0.1 | 0.5 | 1 | 131.3 | 0.001 | 0.5 | 0 |
| 15 | 491.4 | 0.1 | 0 | 1 | 143.0 | 0.001 | 1 | 0 |
| 16 | NA | 0.001 | 0 | 1 | 163.8 | 0.001 | 0 | 1 |
| 17 | NA | 0.001 | 0.5 | 1 | 163.8 | 0.001 | 0.5 | 1 |
| 18 | NA | 0.001 | 1 | 1 | 169.2 | 0.001 | 1 | 1 |

| Rank | Pairwise genetic differentiation | | | | | | | |
| --- | --- | --- | --- | --- | --- | --- | --- | --- |
|  | *G. fossarum* type A | | | | *G. fossarum* type B | | | |
|  | MPO | *d* | *W* | *K* | MPO | *d* | *W* | *K* |
| 1 | 0.082 | 0.01 | 1 | 0 | 0.064 | 0.1 | 1 | 0 |
| 2 | 0.084 | 0.01 | 0 | 0 | 0.068 | 0.1 | 0.5 | 0 |
| 3 | 0.089 | 0.01 | 0.5 | 0 | 0.071 | 0.1 | 0 | 0 |
| 4 | 0.092 | 0.01 | 0 | 1 | 0.071 | 0.1 | 1 | 1 |
| 5 | 0.104 | 0.01 | 0.5 | 1 | 0.075 | 0.01 | 0.5 | 0 |
| 6 | 0.109 | 0.001 | 0 | 0 | 0.077 | 0.1 | 0.5 | 1 |
| 7 | 0.117 | 0.01 | 1 | 1 | 0.085 | 0.01 | 0 | 1 |
| 8 | 0.132 | 0.001 | 0.5 | 0 | 0.087 | 0.1 | 0 | 1 |
| 9 | 0.156 | 0.001 | 1 | 0 | 0.093 | 0.01 | 0 | 0 |
| 10 | 0.180 | 0.1 | 1 | 1 | 0.095 | 0.01 | 1 | 0 |
| 11 | 0.188 | 0.1 | 0 | 0 | 0.105 | 0.01 | 0.5 | 1 |
| 12 | 0.192 | 0.1 | 0.5 | 0 | 0.136 | 0.01 | 1 | 1 |
| 13 | 0.195 | 0.1 | 1 | 0 | 0.245 | 0.001 | 0 | 0 |
| 14 | 0.206 | 0.1 | 0.5 | 1 | 0.253 | 0.001 | 0.5 | 0 |
| 15 | 0.224 | 0.1 | 0 | 1 | 0.274 | 0.001 | 1 | 0 |
| 16 | NA | 0.001 | 0 | 1 | 0.310 | 0.001 | 0.5 | 1 |
| 17 | NA | 0.001 | 0.5 | 1 | 0.311 | 0.001 | 0 | 1 |
| 18 | NA | 0.001 | 1 | 1 | 0.317 | 0.001 | 1 | 1 |

**Table S27** Ranking of the 18 stochastic simulations for their fit to the empirically assessed pairwise genetic differentiation (Nei F_ST_) values according to their median of perpendicular offsets (MPO). Listed are the value for MPO and the corresponding varying parameter values (dispersal rate *d*, upstream movement probability *W*, and scaling of habitat carrying capacity *K*).


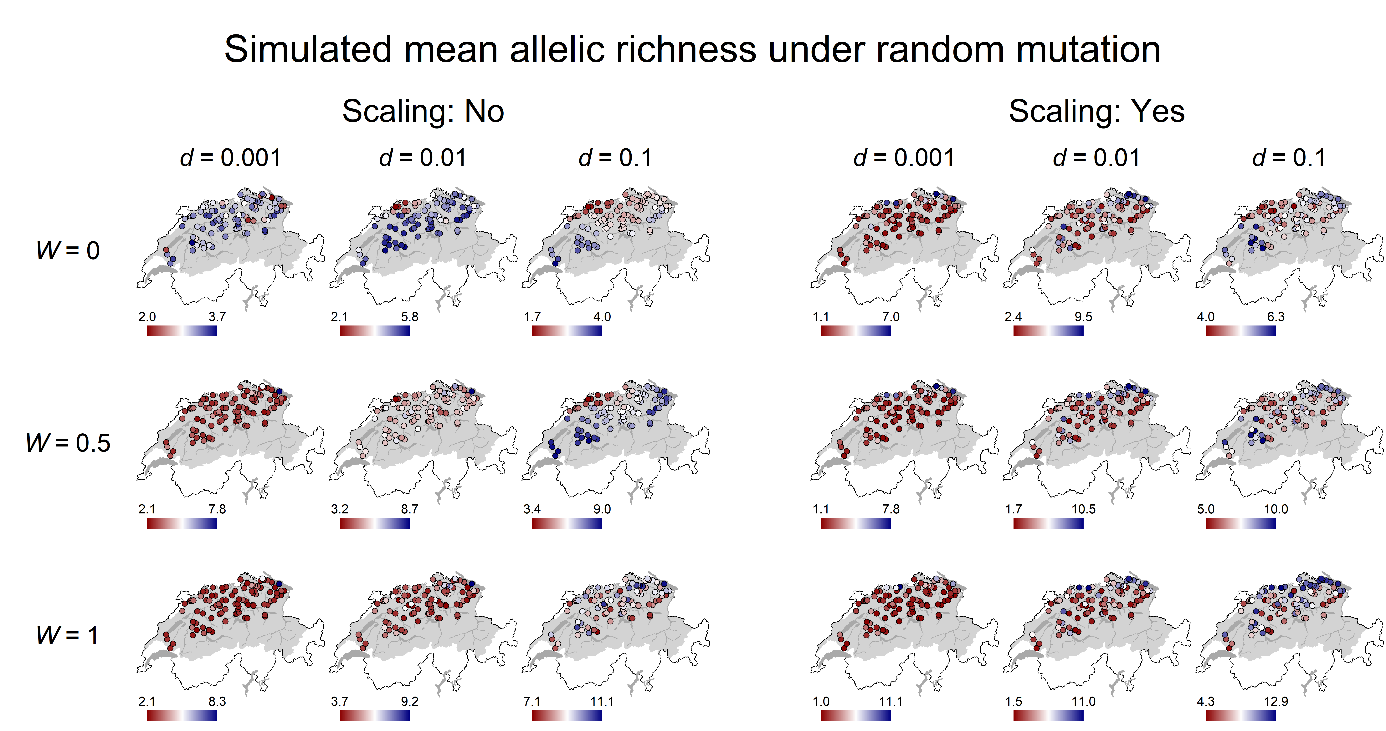


**Figure S28** Maps depicting the predicted mean allelic richness for all 18 stochastic simulation scenarios show different spatial structuring along the Rhine riverine network of Switzerland. The gradient legends show mean allelic richness. Their scale is individually adjusted in each map for the best representation of spatial structuring. The corresponding figure for mean observed heterozygosity is given in Figure S34, the one for expected heterozygosity is given in Figure S40. Geodata source: Federal Office of Topography & Federal Office for the Environment.


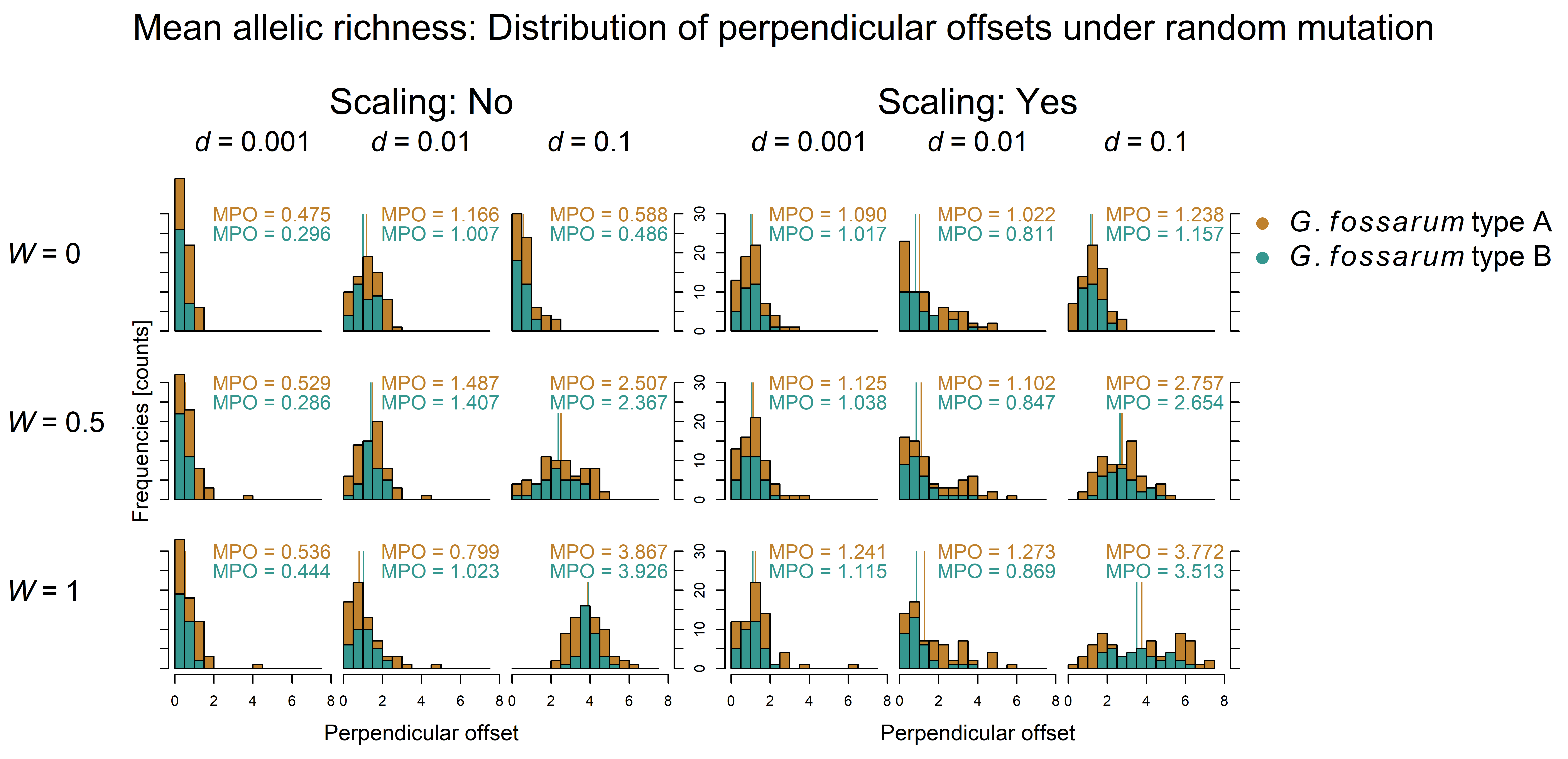


**Figure S29** Histograms and medians of the perpendicular offsets (MPO) between all 18 stochastic simulation scenarios and the empirically assessed mean allelic richness values for both species of the *Gammarus fossarum* complex (type A: orange colour, type B: cyan colour). The more left-skewed a distribution, the better the fit of simulated values to empirical data. The actual perpendicular offsets of all 18 scenarios is given in Figure S30.
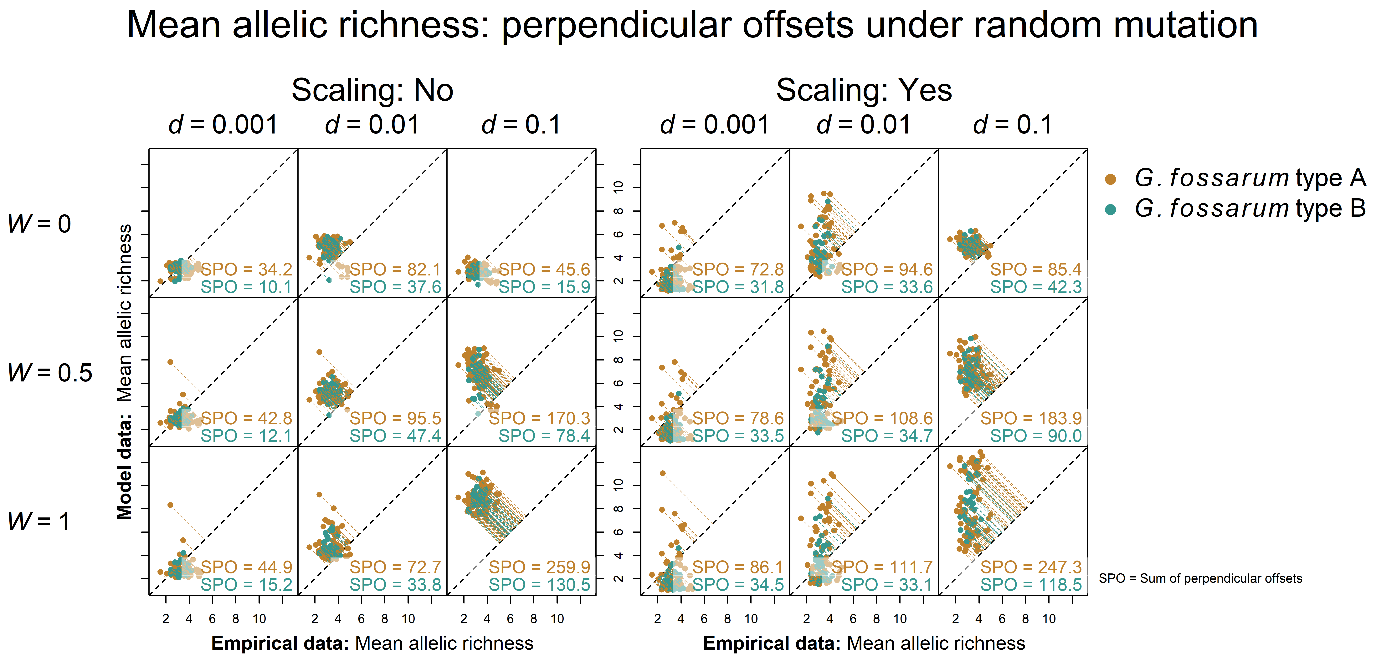


**Figure S30** Perpendicular offsets between mean allelic richness value pairs of all 18 stochastic simulation scenarios and the empirical data. The sum of the perpendicular offsets (SPO) served as a goodness-of-fit measure. SPO takes into account the overall spread of simulated values from their empirical counterpart, where larger SPO indicates a poorer fit.


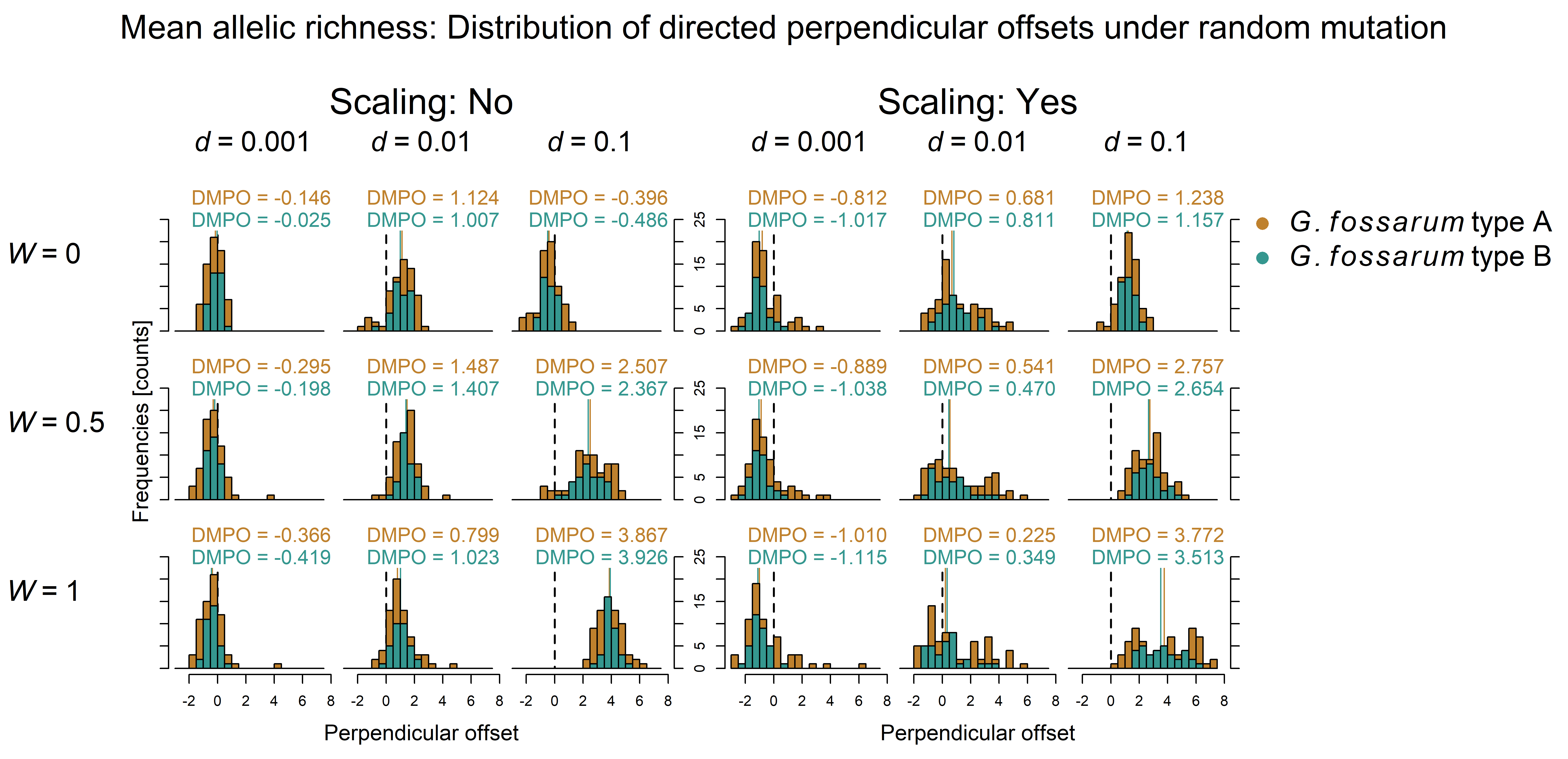
**Figure S31** Histograms and medians of the directed perpendicular offsets (DMPO) between all 18 stochastic simulation scenarios and the empirically assessed mean allelic richness values. The directed perpendicular offset does take into account if points are above or below the vertical (1:1) line. The dotted vertical line shows zero offset, corresponding to a perfect fit between simulation and empirical data.**Table S32** Ranking of the 18 stochastic simulations for their fit to the empirically assessed mean allelic richness values according to their sum of perpendicular offsets (SPO). Listed are the value for SPO and the corresponding varying parameter values (dispersal rate *d*, upstream movement probability *W*, and scaling of carrying capacity *K*).

| Rank | Mean allelic richness | | | | | | | |
| --- | --- | --- | --- | --- | --- | --- | --- | --- |
|  | *G. fossarum* type A | | | | *G. fossarum* type B | | | |
|  | SPO | *d* | *W* | *K* | SPO | *d* | *W* | *K* |
| 1 | 34.20 | 0.001 | 0 | 0 | 10.07 | 0.001 | 0 | 0 |
| 2 | 42.84 | 0.001 | 0.5 | 0 | 12.14 | 0.001 | 0.5 | 0 |
| 3 | 44.88 | 0.001 | 1 | 0 | 15.16 | 0.001 | 1 | 0 |
| 4 | 45.63 | 0.1 | 0 | 0 | 15.92 | 0.1 | 0 | 0 |
| 5 | 72.67 | 0.01 | 1 | 0 | 31.77 | 0.001 | 0 | 1 |
| 6 | 72.81 | 0.001 | 0 | 1 | 33.10 | 0.01 | 1 | 1 |
| 7 | 78.64 | 0.001 | 0.5 | 1 | 33.53 | 0.001 | 0.5 | 1 |
| 8 | 82.06 | 0.01 | 0 | 0 | 33.57 | 0.01 | 0 | 1 |
| 9 | 85.41 | 0.1 | 0 | 1 | 33.83 | 0.01 | 1 | 0 |
| 10 | 86.09 | 0.001 | 1 | 1 | 34.53 | 0.001 | 1 | 1 |
| 11 | 94.64 | 0.01 | 0 | 1 | 34.65 | 0.01 | 0.5 | 1 |
| 12 | 95.54 | 0.01 | 0.5 | 0 | 37.56 | 0.01 | 0 | 0 |
| 13 | 108.62 | 0.01 | 0.5 | 1 | 42.34 | 0.1 | 0 | 1 |
| 14 | 111.71 | 0.01 | 1 | 1 | 47.41 | 0.01 | 0.5 | 0 |
| 15 | 170.27 | 0.1 | 0.5 | 0 | 78.36 | 0.1 | 0.5 | 0 |
| 16 | 183.95 | 0.1 | 0.5 | 1 | 90.00 | 0.1 | 0.5 | 1 |
| 17 | 247.25 | 0.1 | 1 | 1 | 118.53 | 0.1 | 1 | 1 |
| 18 | 259.93 | 0.1 | 1 | 0 | 130.50 | 0.1 | 1 | 0 |

| Rank | Mean allelic richness | | | | | | | |
| --- | --- | --- | --- | --- | --- | --- | --- | --- |
|  | *G. fossarum* type A | | | | *G. fossarum* type B | | | |
|  | MPO | *d* | *W* | *K* | MPO | *d* | *W* | *K* |
| 1 | 0.475 | 0.001 | 0 | 0 | 0.286 | 0.001 | 0.5 | 0 |
| 2 | 0.529 | 0.001 | 0.5 | 0 | 0.296 | 0.001 | 0 | 0 |
| 3 | 0.536 | 0.001 | 1 | 0 | 0.444 | 0.001 | 1 | 0 |
| 4 | 0.588 | 0.1 | 0 | 0 | 0.486 | 0.1 | 0 | 0 |
| 5 | 0.799 | 0.01 | 1 | 0 | 0.811 | 0.01 | 0 | 1 |
| 6 | 1.022 | 0.01 | 0 | 1 | 0.847 | 0.01 | 0.5 | 1 |
| 7 | 1.090 | 0.001 | 0 | 1 | 0.869 | 0.01 | 1 | 1 |
| 8 | 1.102 | 0.01 | 0.5 | 1 | 1.007 | 0.01 | 0 | 0 |
| 9 | 1.125 | 0.001 | 0.5 | 1 | 1.017 | 0.001 | 0 | 1 |
| 10 | 1.166 | 0.01 | 0 | 0 | 1.023 | 0.01 | 1 | 0 |
| 11 | 1.238 | 0.1 | 0 | 1 | 1.038 | 0.001 | 0.5 | 1 |
| 12 | 1.241 | 0.001 | 1 | 1 | 1.115 | 0.001 | 1 | 1 |
| 13 | 1.273 | 0.01 | 1 | 1 | 1.157 | 0.1 | 0 | 1 |
| 14 | 1.487 | 0.01 | 0.5 | 0 | 1.407 | 0.01 | 0.5 | 0 |
| 15 | 2.507 | 0.1 | 0.5 | 0 | 2.367 | 0.1 | 0.5 | 0 |
| 16 | 2.757 | 0.1 | 0.5 | 1 | 2.654 | 0.1 | 0.5 | 1 |
| 17 | 3.772 | 0.1 | 1 | 1 | 3.513 | 0.1 | 1 | 1 |
| 18 | 3.867 | 0.1 | 1 | 0 | 3.926 | 0.1 | 1 | 0 |

**Table S33** Ranking of the 18 stochastic simulations for their fit to the empirically assessed mean allelic richness values according to their median of perpendicular offsets (MPO). Listed are the value for MPO and the corresponding varying parameter values (dispersal rate *d*, upstream movement probability *W*, and scaling of carrying capacity *K*).
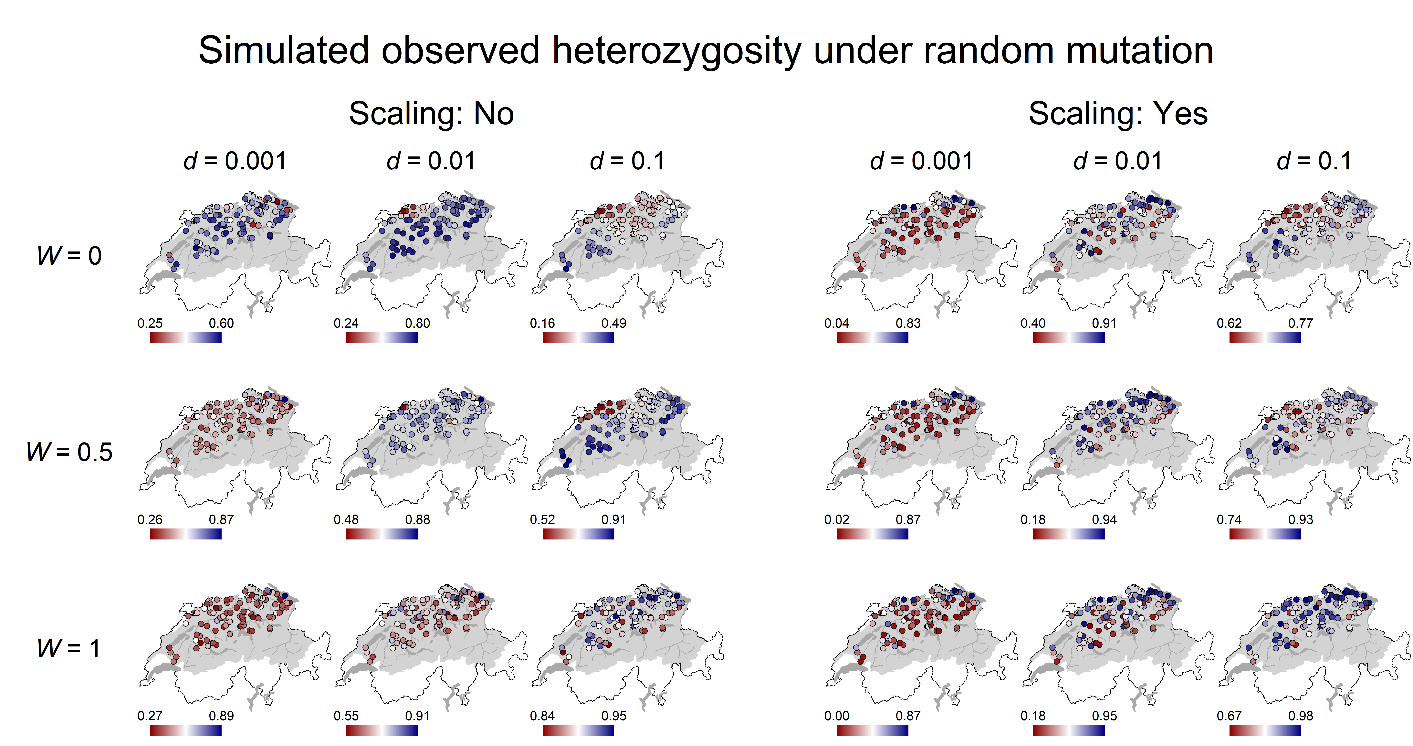


**Figure S34** Maps depicting the predicted mean observed heterozygosity for all 18 stochastic simulation scenarios show different spatial structuring along the Rhine riverine network of Switzerland. The gradient legends show mean observed heterozygosity. Their ranges are adjusted for each map for the best visual representation of spatial structuring. Geodata source: Federal Office of Topography & Federal Office for the Environment.


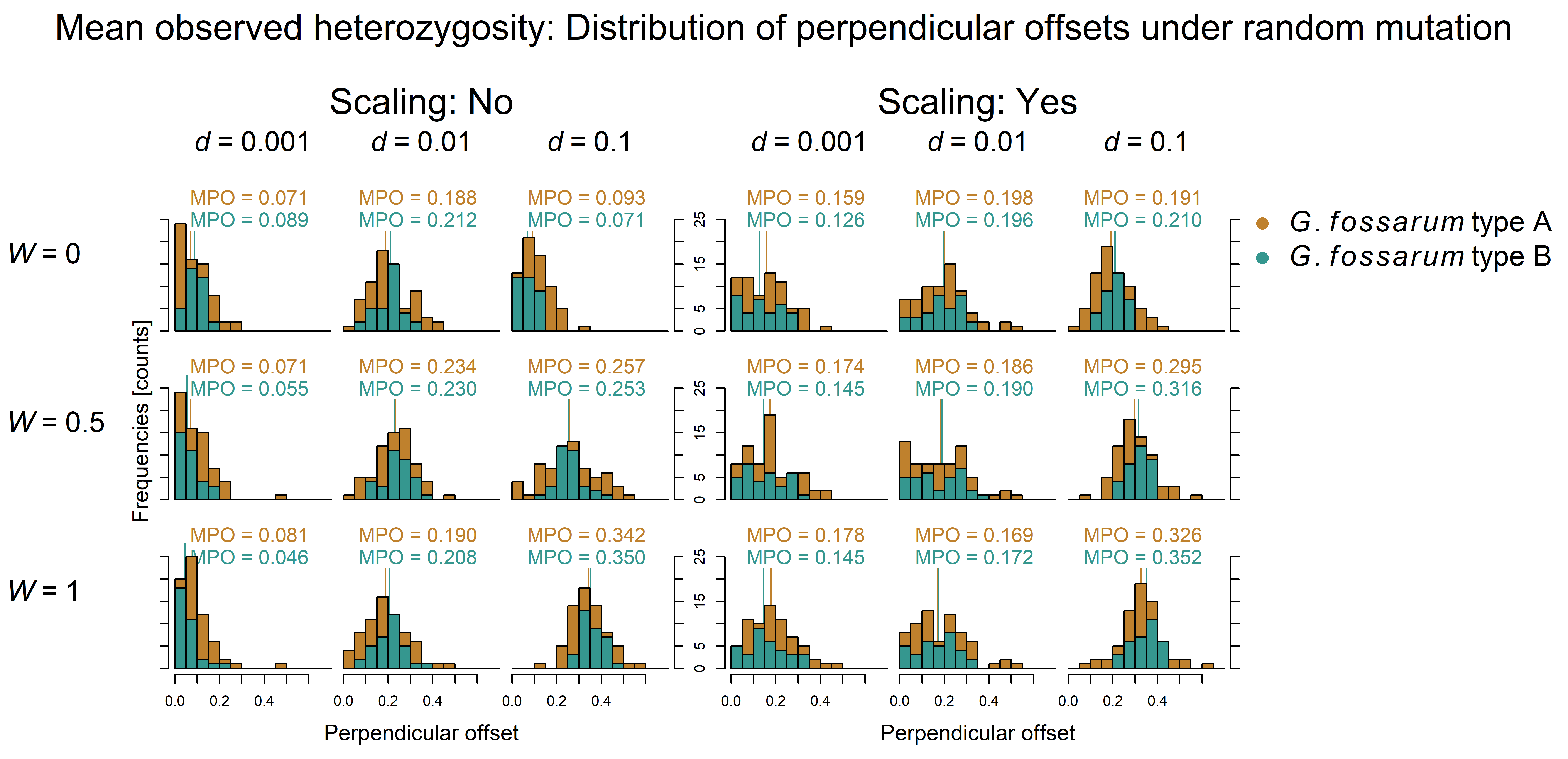
**Figure S35** Histograms and medians of the perpendicular offsets (MPO) between all 18 stochastic simulation scenarios and the empirical mean observed heterozygosity values for both species of the *Gammarus fossarum* complex (type A: orange symbols, type B: cyan symbols). The actual perpendicular offsets of all 18 scenarios is given in Figure S36.
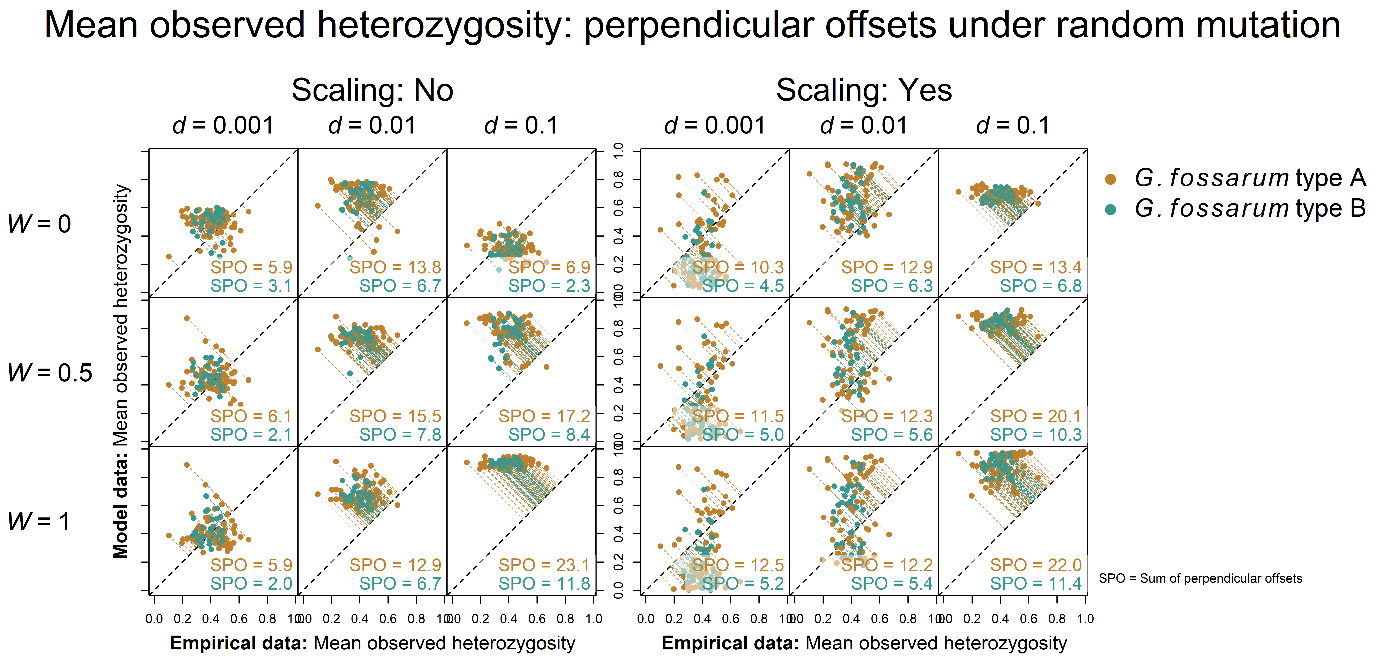


**Figure S36** Perpendicular offsets between mean observed heterozygosity value pairs of all 18 stochastic simulation scenarios and the empirical data. The sum of the perpendicular offsets (SPO) served as a goodness-of-fit measure. SPO takes into account the overall spread of simulated values from their empirical counterpart, where larger SPO indicates a poorer fit.


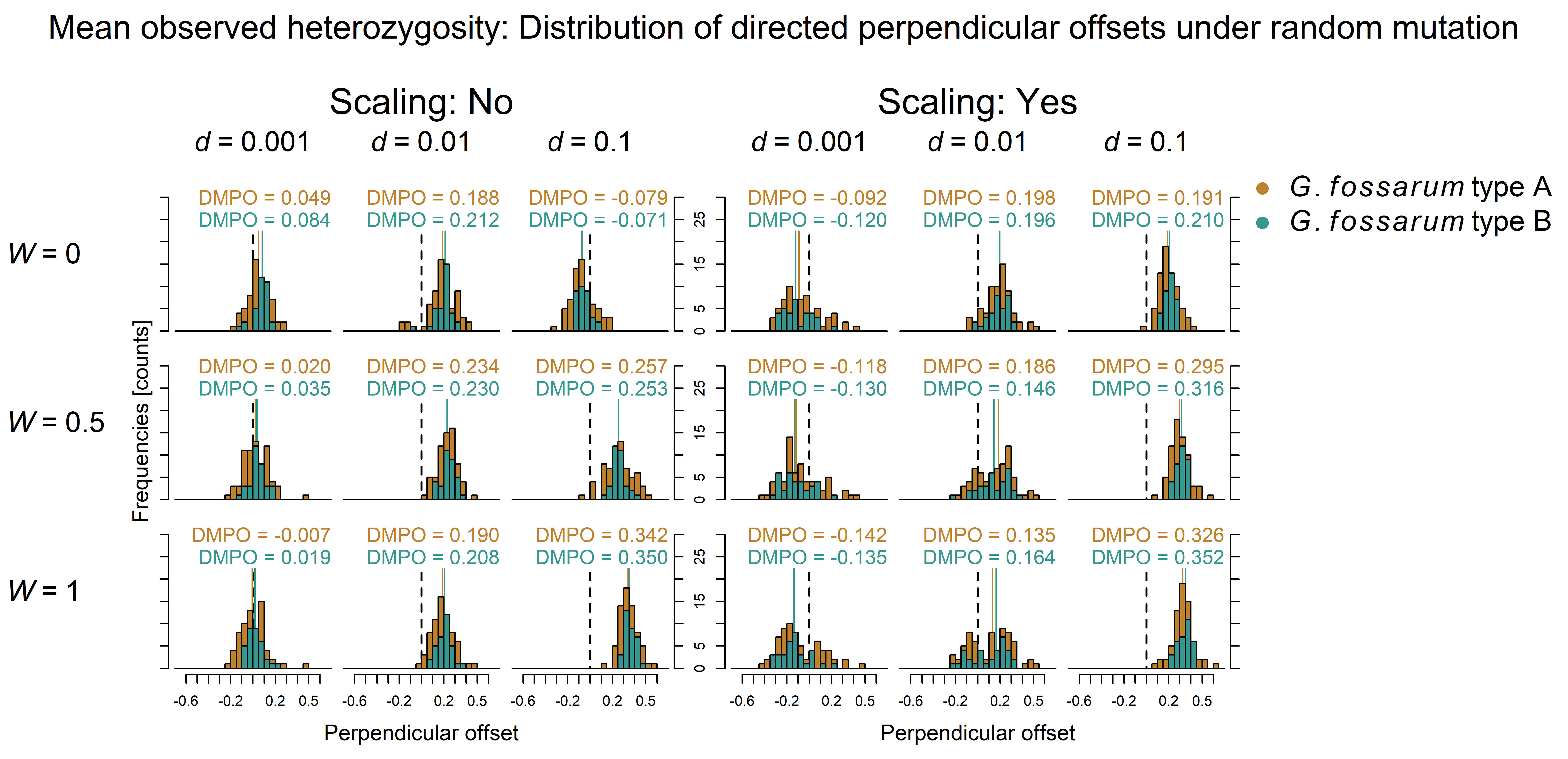


**Figure S37** Histograms and medians of the directed perpendicular offsets (DMPO) between all 18 stochastic simulation scenarios and the empirically assessed mean observed heterozygosity values. The directed perpendicular offset does take into account if points are above or below the vertical (1:1) line. The dotted vertical line shows zero offset, corresponding to a perfect fit between simulation and empirical data.

| Rank | Mean observed heterozygosity | | | | | | | |
| --- | --- | --- | --- | --- | --- | --- | --- | --- |
|  | *G. fossarum* type A | | | | *G. fossarum* type B | | | |
|  | SPO | *d* | *W* | *K* | SPO | *d* | *W* | *K* |
| 1 | 5.91 | 0.001 | 0 | 0 | 2.03 | 0.001 | 1 | 0 |
| 2 | 5.95 | 0.001 | 1 | 0 | 2.11 | 0.001 | 0.5 | 0 |
| 3 | 6.05 | 0.001 | 0.5 | 0 | 2.31 | 0.1 | 0 | 0 |
| 4 | 6.91 | 0.1 | 0 | 0 | 3.10 | 0.001 | 0 | 0 |
| 5 | 10.27 | 0.001 | 0 | 1 | 4.54 | 0.001 | 0 | 1 |
| 6 | 11.51 | 0.001 | 0.5 | 1 | 4.97 | 0.001 | 0.5 | 1 |
| 7 | 12.24 | 0.01 | 1 | 1 | 5.17 | 0.001 | 1 | 1 |
| 8 | 12.31 | 0.01 | 0.5 | 1 | 5.36 | 0.01 | 1 | 1 |
| 9 | 12.46 | 0.001 | 1 | 1 | 5.62 | 0.01 | 0.5 | 1 |
| 10 | 12.89 | 0.01 | 1 | 0 | 6.32 | 0.01 | 0 | 1 |
| 11 | 12.89 | 0.01 | 0 | 1 | 6.66 | 0.01 | 1 | 0 |
| 12 | 13.39 | 0.1 | 0 | 1 | 6.70 | 0.01 | 0 | 0 |
| 13 | 13.75 | 0.01 | 0 | 0 | 6.84 | 0.1 | 0 | 1 |
| 14 | 15.48 | 0.01 | 0.5 | 0 | 7.84 | 0.01 | 0.5 | 0 |
| 15 | 17.23 | 0.1 | 0.5 | 0 | 8.42 | 0.1 | 0.5 | 0 |
| 16 | 20.14 | 0.1 | 0.5 | 1 | 10.32 | 0.1 | 0.5 | 1 |
| 17 | 21.96 | 0.1 | 1 | 1 | 11.37 | 0.1 | 1 | 1 |
| 18 | 23.06 | 0.1 | 1 | 0 | 11.75 | 0.1 | 1 | 0 |

**Table S38** Ranking of the 18 stochastic simulations for their fit to the empirically assessed mean observed heterozygosity values according to their sum of perpendicular offsets (SPO). Listed are the value for SPO and the corresponding varying parameter values (dispersal rate *d*, upstream movement probability *W*, and scaling of carrying capacity *K*).

| Rank | Mean observed heterozygosity | | | | | | | |
| --- | --- | --- | --- | --- | --- | --- | --- | --- |
|  | *G. fossarum* type A | | | | *G. fossarum* type B | | | |
|  | MPO | *d* | *W* | *K* | MPO | *d* | *W* | *K* |
| 1 | 0.071 | 0.001 | 0.5 | 0 | 0.046 | 0.001 | 1 | 0 |
| 2 | 0.071 | 0.001 | 0 | 0 | 0.055 | 0.001 | 0.5 | 0 |
| 3 | 0.081 | 0.001 | 1 | 0 | 0.071 | 0.1 | 0 | 0 |
| 4 | 0.093 | 0.1 | 0 | 0 | 0.089 | 0.001 | 0 | 0 |
| 5 | 0.159 | 0.001 | 0 | 1 | 0.126 | 0.001 | 0 | 1 |
| 6 | 0.169 | 0.01 | 1 | 1 | 0.145 | 0.001 | 1 | 1 |
| 7 | 0.174 | 0.001 | 0.5 | 1 | 0.145 | 0.001 | 0.5 | 1 |
| 8 | 0.178 | 0.001 | 1 | 1 | 0.172 | 0.01 | 1 | 1 |
| 9 | 0.186 | 0.01 | 0.5 | 1 | 0.190 | 0.01 | 0.5 | 1 |
| 10 | 0.188 | 0.01 | 0 | 0 | 0.196 | 0.01 | 0 | 1 |
| 11 | 0.190 | 0.01 | 1 | 0 | 0.208 | 0.01 | 1 | 0 |
| 12 | 0.191 | 0.1 | 0 | 1 | 0.210 | 0.1 | 0 | 1 |
| 13 | 0.198 | 0.01 | 0 | 1 | 0.212 | 0.01 | 0 | 0 |
| 14 | 0.234 | 0.01 | 0.5 | 0 | 0.230 | 0.01 | 0.5 | 0 |
| 15 | 0.257 | 0.1 | 0.5 | 0 | 0.253 | 0.1 | 0.5 | 0 |
| 16 | 0.295 | 0.1 | 0.5 | 1 | 0.316 | 0.1 | 0.5 | 1 |
| 17 | 0.326 | 0.1 | 1 | 1 | 0.350 | 0.1 | 1 | 0 |
| 18 | 0.342 | 0.1 | 1 | 0 | 0.352 | 0.1 | 1 | 1 |

**Table S39** Ranking of the 18 stochastic simulations for their fit to the empirically assessed mean observed heterozygosity values according to their median of perpendicular offsets (MPO). Listed are the value for MPO and the corresponding varying parameter values (dispersal rate *d*, upstream movement probability *W*, and scaling of habitat carrying capacity *K*).


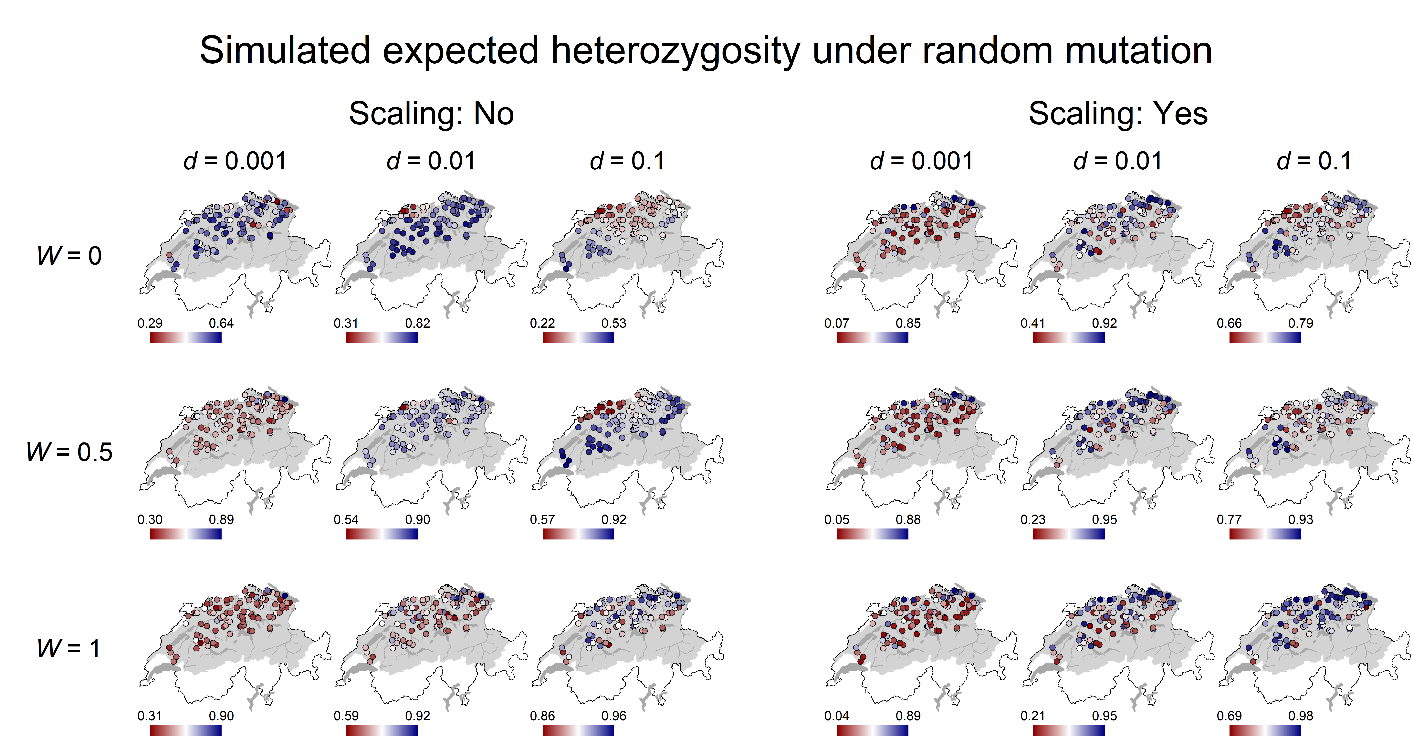


**Figure S40** Maps depicting the predicted expected heterozygosity for all 18 stochastic simulation scenarios show different spatial structuring along the Rhine riverine network of Switzerland. The gradient legends show expected heterozygosity. Their ranges are adjusted for each map for the best visual representation of spatial structuring. Geodata source: Federal Office of Topography & Federal Office for the Environment.


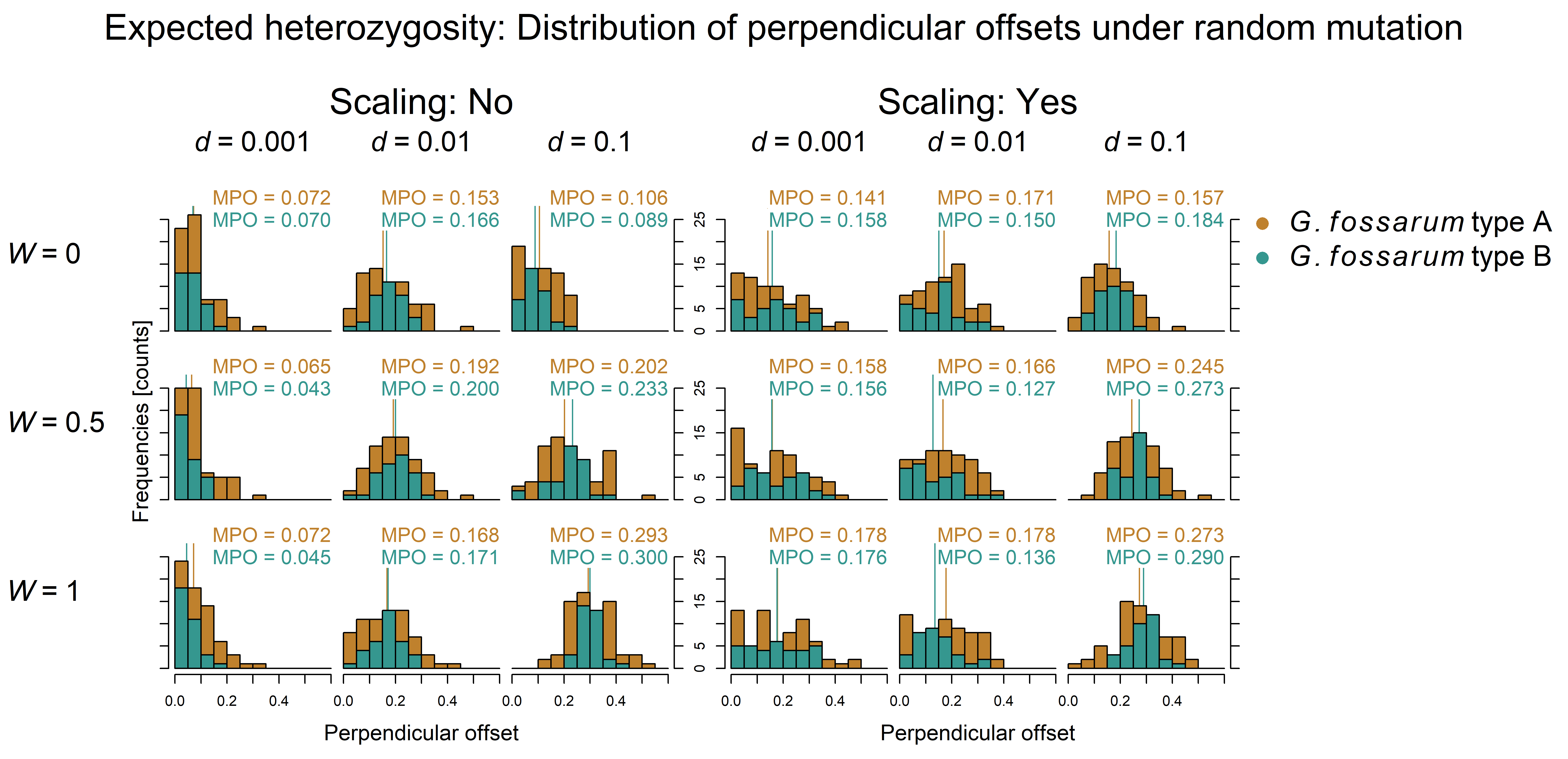


**Figure S41** Histograms and medians of the perpendicular offsets (MPO) between all 18 stochastic simulation scenarios and the empirical expected heterozygosity values for both species of the *Gammarus fossarum* complex (type A: orange symbols, type B: cyan symbols). The actual perpendicular offsets of all 18 scenarios is given in Figure S42.


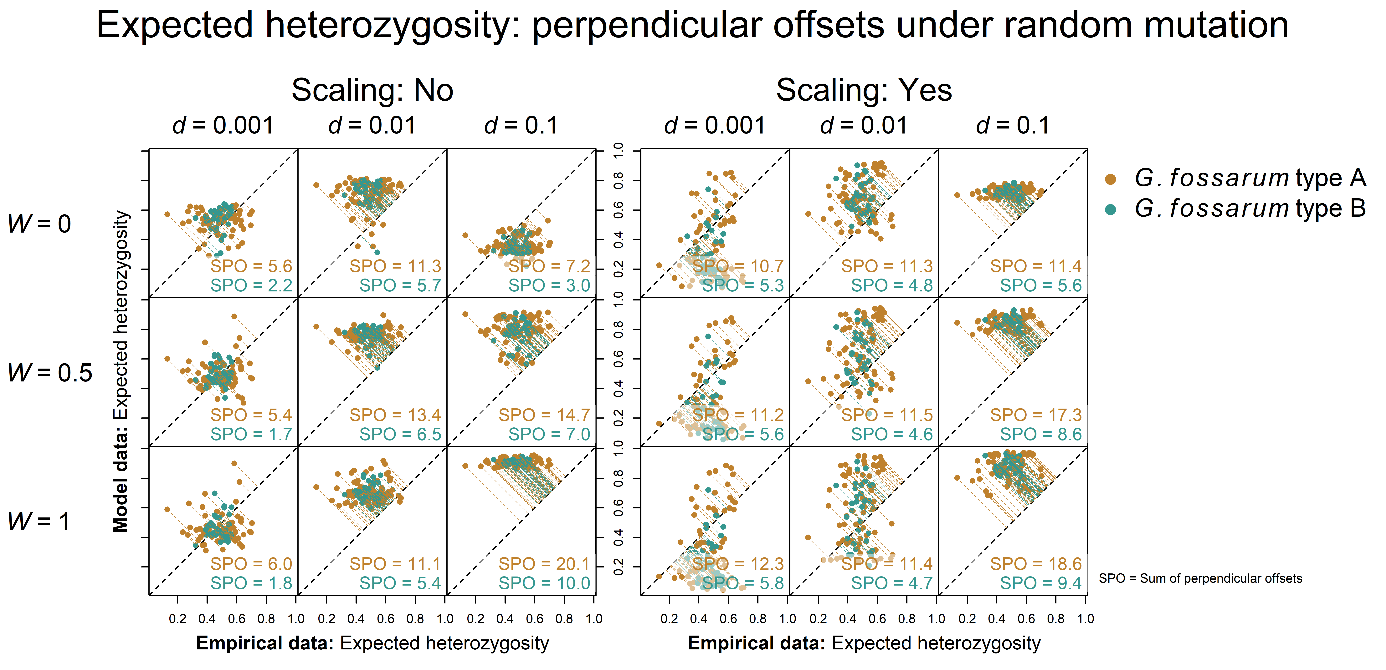


**Figure S42** Perpendicular offsets between expected heterozygosity value pairs of all 18 stochastic simulation scenarios and the empirical data. The sum of the perpendicular offsets (SPO) served as a goodness-of-fit measure. SPO takes into account the overall spread of simulated values from their empirical counterpart, where larger SPO indicates a poorer fit.


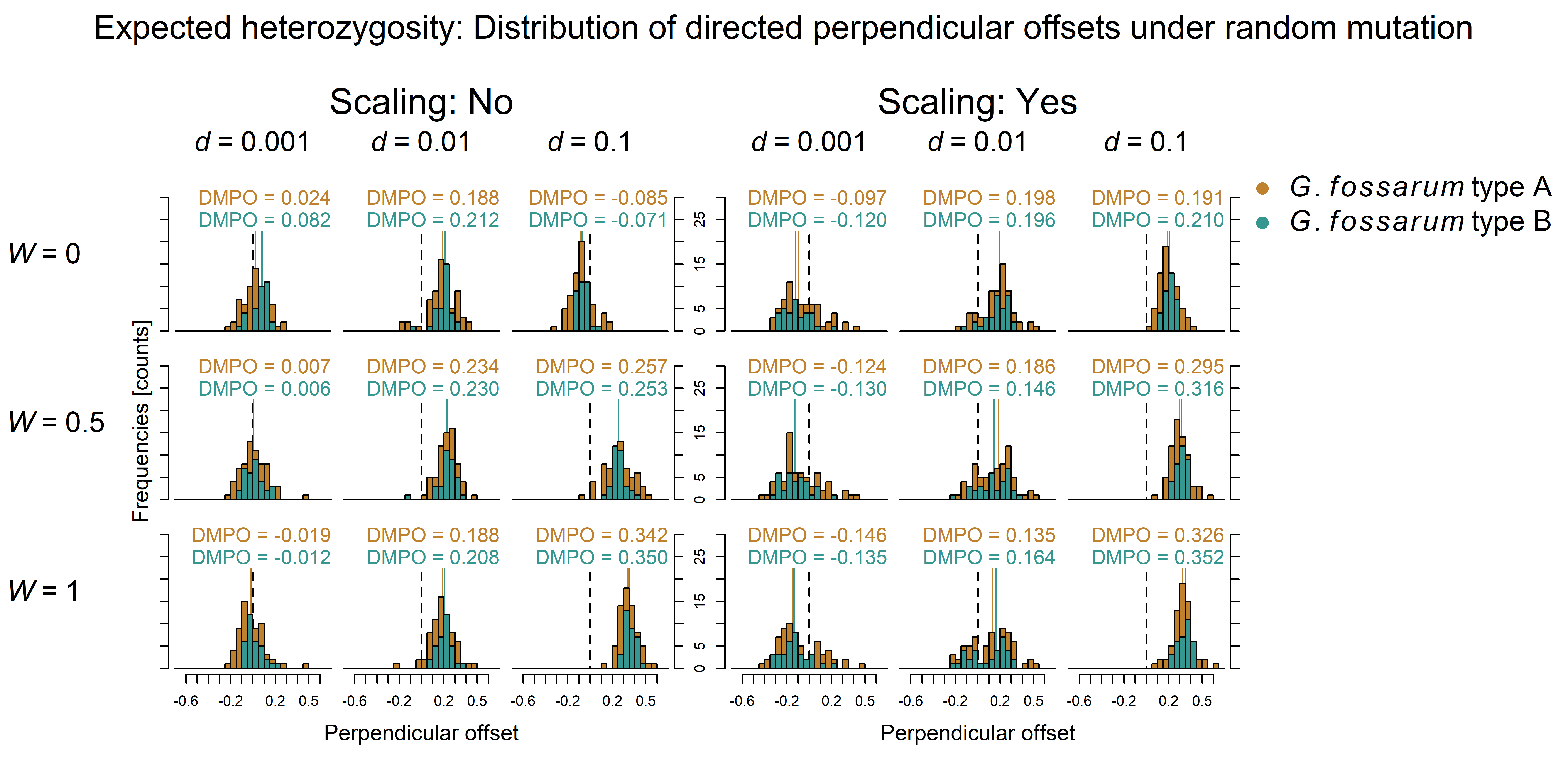


**Figure S43** Histograms and medians of the directed perpendicular offsets (DMPO) between all 18 stochastic simulation scenarios and the empirically assessed expected heterozygosity values. The directed perpendicular offset does take into account if points are above or below the vertical (1:1) line. The dotted vertical line shows zero offset, corresponding to a perfect fit between simulation and empirical data.

| Rank | Expected heterozygosity | | | | | | | |
| --- | --- | --- | --- | --- | --- | --- | --- | --- |
|  | *G. fossarum* type A | | | | *G. fossarum* type B | | | |
|  | SPO | *d* | *W* | *K* | SPO | *d* | *W* | *K* |
| 1 | 5.42 | 0.001 | 0.5 | 0 | 1.67 | 0.001 | 0.5 | 0 |
| 2 | 5.64 | 0.001 | 0 | 0 | 1.76 | 0.001 | 1 | 0 |
| 3 | 6.02 | 0.001 | 1 | 0 | 2.18 | 0.001 | 0 | 0 |
| 4 | 7.17 | 0.1 | 0 | 0 | 2.98 | 0.1 | 0 | 0 |
| 5 | 10.67 | 0.001 | 0 | 1 | 4.55 | 0.01 | 0.5 | 1 |
| 6 | 11.05 | 0.01 | 1 | 0 | 4.72 | 0.01 | 1 | 1 |
| 7 | 11.21 | 0.001 | 0.5 | 1 | 4.80 | 0.01 | 0 | 1 |
| 8 | 11.26 | 0.01 | 0 | 0 | 5.26 | 0.001 | 0 | 1 |
| 9 | 11.29 | 0.01 | 0 | 1 | 5.38 | 0.01 | 1 | 0 |
| 10 | 11.37 | 0.01 | 1 | 1 | 5.58 | 0.001 | 0.5 | 1 |
| 11 | 11.41 | 0.1 | 0 | 1 | 5.63 | 0.1 | 0 | 1 |
| 12 | 11.47 | 0.01 | 0.5 | 1 | 5.72 | 0.01 | 0 | 0 |
| 13 | 12.32 | 0.001 | 1 | 1 | 5.78 | 0.001 | 1 | 1 |
| 14 | 13.36 | 0.01 | 0.5 | 0 | 6.51 | 0.01 | 0.5 | 0 |
| 15 | 14.74 | 0.1 | 0.5 | 0 | 7.04 | 0.1 | 0.5 | 0 |
| 16 | 17.29 | 0.1 | 0.5 | 1 | 8.55 | 0.1 | 0.5 | 1 |
| 17 | 18.60 | 0.1 | 1 | 1 | 9.43 | 0.1 | 1 | 1 |
| 18 | 20.13 | 0.1 | 1 | 0 | 10.02 | 0.1 | 1 | 0 |

**Table S44** Ranking of the 18 stochastic simulations for their fit to the empirically assessed expected heterozygosity values according to their sum of perpendicular offsets (SPO). Listed are the value for SPO and the corresponding varying parameter values (dispersal rate *d*, upstream movement probability *W*, and scaling of carrying capacity *K*).

| Rank | Expected heterozygosity | | | | | | | |
| --- | --- | --- | --- | --- | --- | --- | --- | --- |
|  | *G. fossarum* type A | | | | *G. fossarum* type B | | | |
|  | MPO | *d* | *W* | *K* | MPO | *d* | *W* | *K* |
| 1 | 0.065 | 0.001 | 0.5 | 0 | 0.043 | 0.001 | 0.5 | 0 |
| 2 | 0.072 | 0.001 | 0 | 0 | 0.045 | 0.001 | 1 | 0 |
| 3 | 0.072 | 0.001 | 1 | 0 | 0.070 | 0.001 | 0 | 0 |
| 4 | 0.106 | 0.1 | 0 | 0 | 0.089 | 0.1 | 0 | 0 |
| 5 | 0.141 | 0.001 | 0 | 1 | 0.127 | 0.01 | 0.5 | 1 |
| 6 | 0.153 | 0.01 | 0 | 0 | 0.136 | 0.01 | 1 | 1 |
| 7 | 0.157 | 0.1 | 0 | 1 | 0.150 | 0.01 | 0 | 1 |
| 8 | 0.158 | 0.001 | 0.5 | 1 | 0.156 | 0.001 | 0.5 | 1 |
| 9 | 0.166 | 0.01 | 0.5 | 1 | 0.158 | 0.001 | 0 | 1 |
| 10 | 0.168 | 0.01 | 1 | 0 | 0.166 | 0.01 | 0 | 0 |
| 11 | 0.171 | 0.01 | 0 | 1 | 0.171 | 0.01 | 1 | 0 |
| 12 | 0.178 | 0.001 | 1 | 1 | 0.176 | 0.001 | 1 | 1 |
| 13 | 0.178 | 0.01 | 1 | 1 | 0.184 | 0.1 | 0 | 1 |
| 14 | 0.192 | 0.01 | 0.5 | 0 | 0.200 | 0.01 | 0.5 | 0 |
| 15 | 0.202 | 0.1 | 0.5 | 0 | 0.233 | 0.1 | 0.5 | 0 |
| 16 | 0.245 | 0.1 | 0.5 | 1 | 0.273 | 0.1 | 0.5 | 1 |
| 17 | 0.273 | 0.1 | 1 | 1 | 0.290 | 0.1 | 1 | 1 |
| 18 | 0.293 | 0.1 | 1 | 0 | 0.300 | 0.1 | 1 | 0 |

**Table S45** Ranking of the 18 stochastic simulations for their fit to the empirically assessed expected heterozygosity values according to their median of perpendicular offsets (MPO). Listed are the value for MPO and the corresponding varying parameter values (dispersal rate *d*, upstream movement probability *W*, and scaling of habitat carrying capacity *K*).


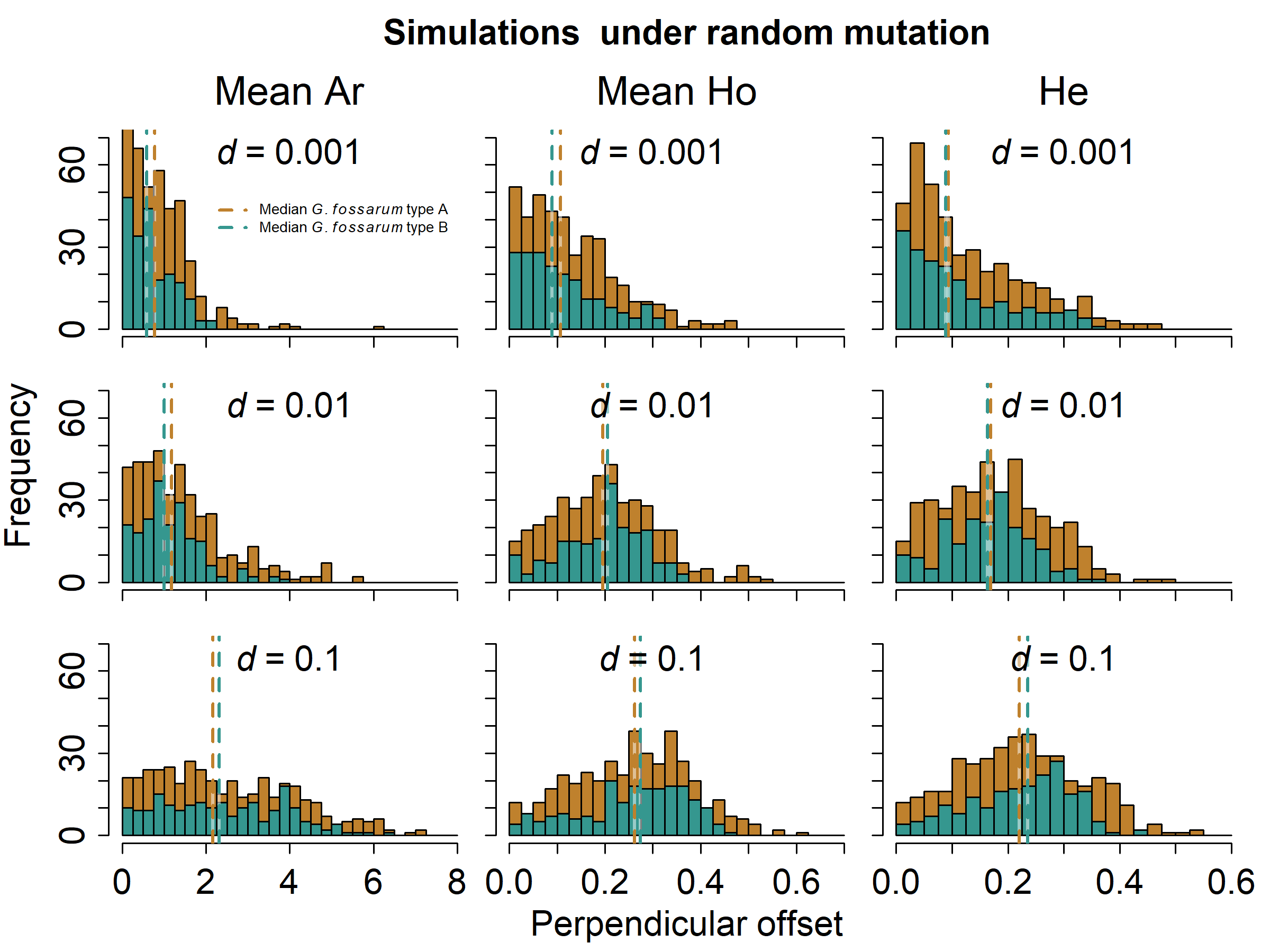


**Figure S46** Different dispersal rates in the simulations resulted in strong variability on the simulation fit, reflected in perpendicular offset differences. Simulations with low dispersal rates (*d* = 0.001) were superior to simulations with higher dispersal rates. High dispersal rates generally resulted in the worst model fits.


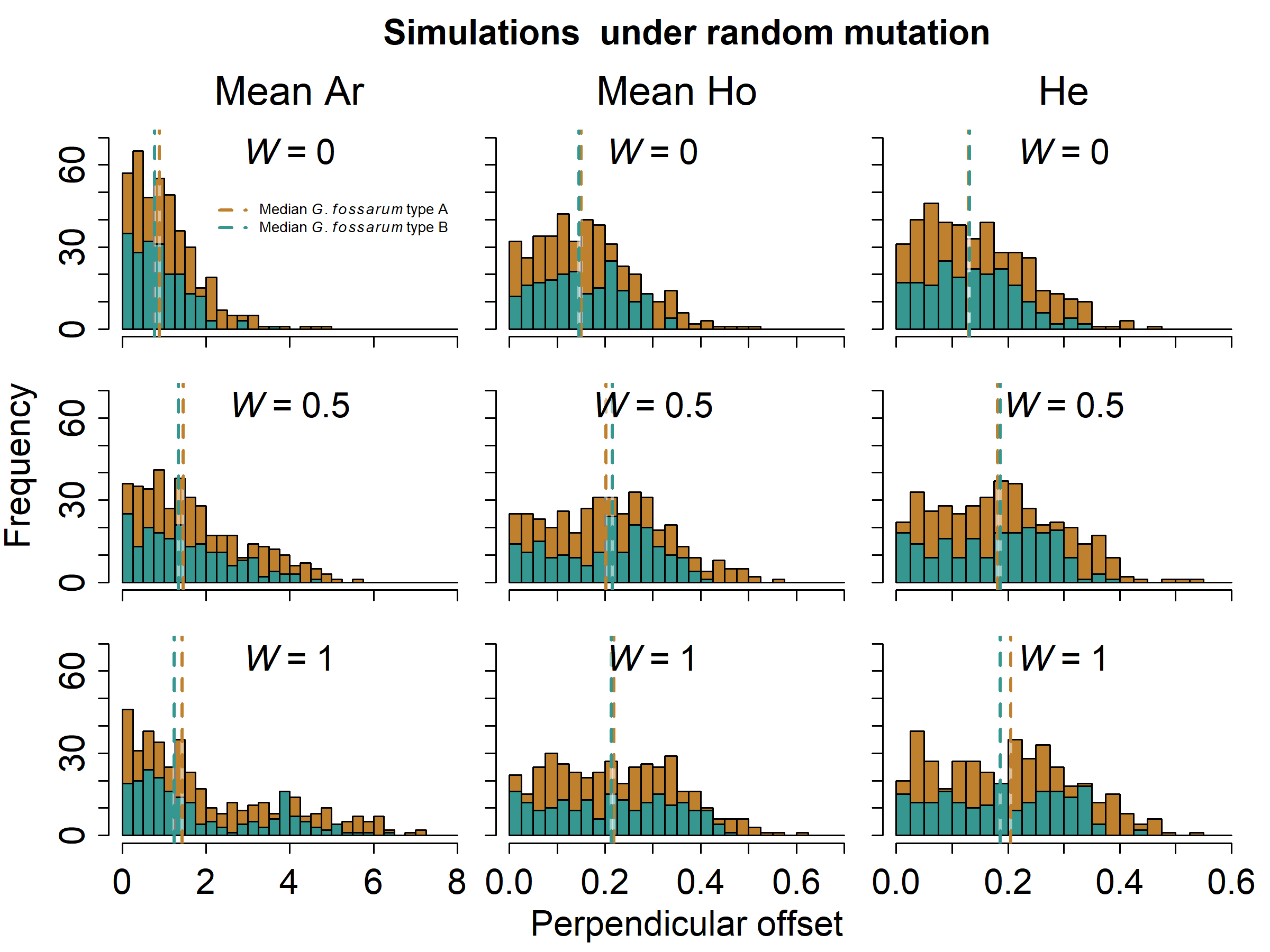


**Figure S47** Changing upstream movement probabilities in the simulations resulted in slight shifts of model fits to empirical data in comparison to dispersal rate (see Fig. S7). The clearest signal results when allowing for upstream dispersal (*W* = 0.5 and *W* = 1), weakening model fits as reflected in perpendicular offset differences. Simulations with no upstream dispersal (*W* = 0) were superior to those simulations.


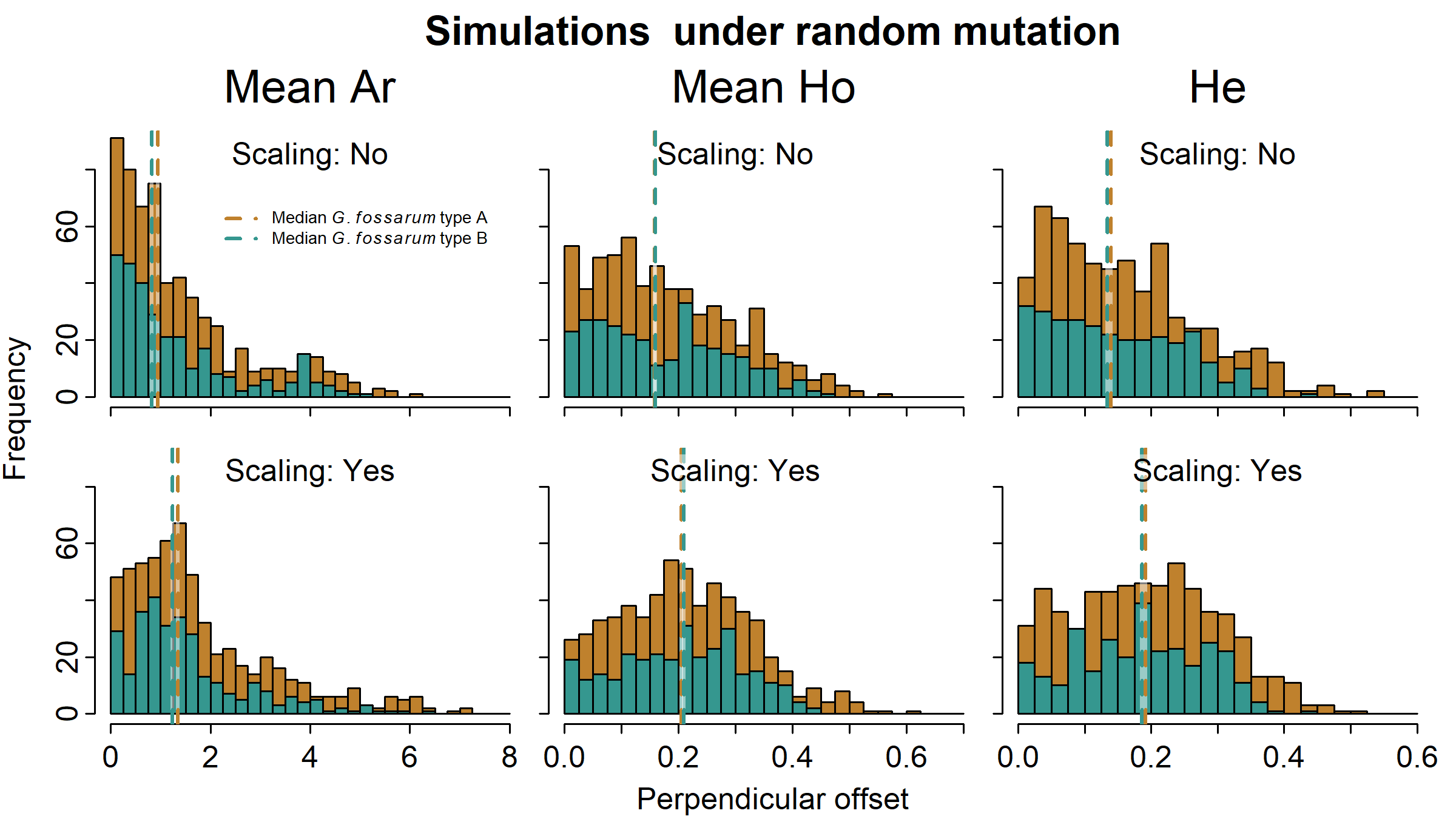


**Figure S48** Scaling the habitat carrying capacity showed the lowest impact on the variability of the response variable. Generally, simulations without scaling of the carrying capacity (*K* = 0) outperformed the ones where carrying capacity scaled with the square-root of the total catchment area.


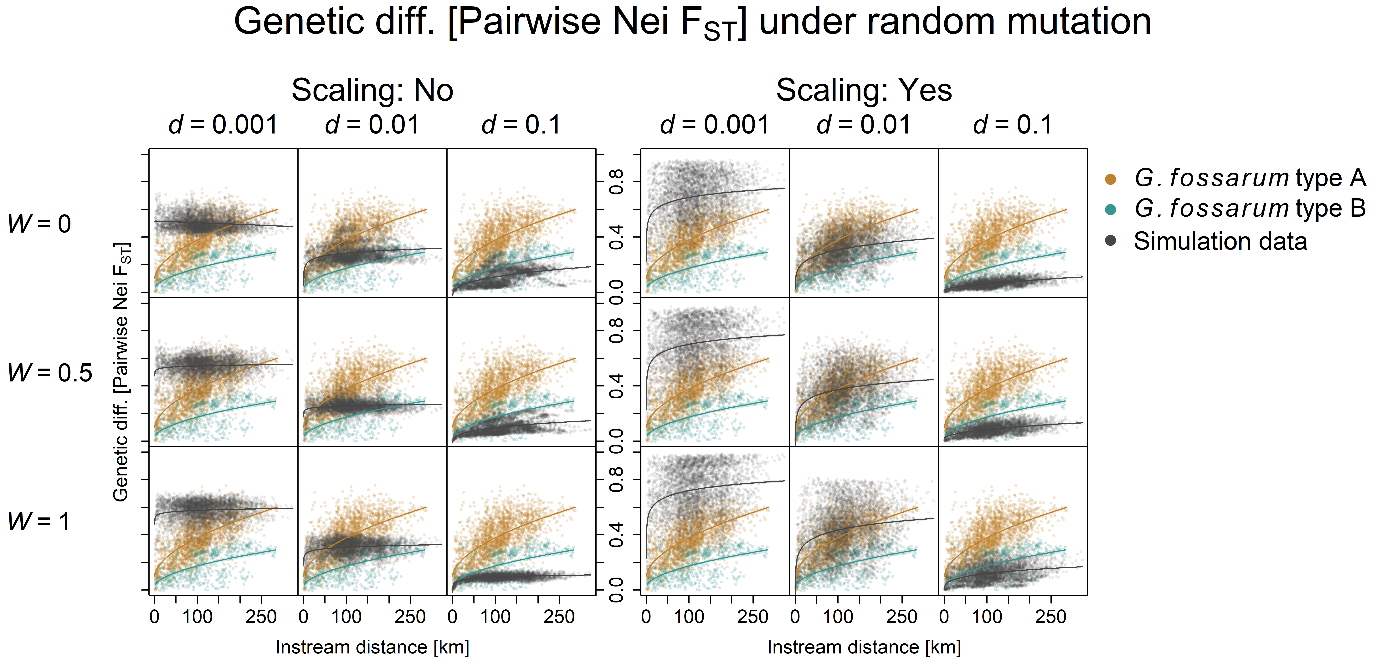


**Figure S49** Pairwise genetic differentiation F_ST_ plotted against the instream distance between nodes of the riverine network (Lines: LM with power term; shading depicts 95% confidence interval). Some simulated data matched the empirical data relatively well, others were completely off. The best-fitting simulations were based on moderate dispersal rates (d = 0.01).


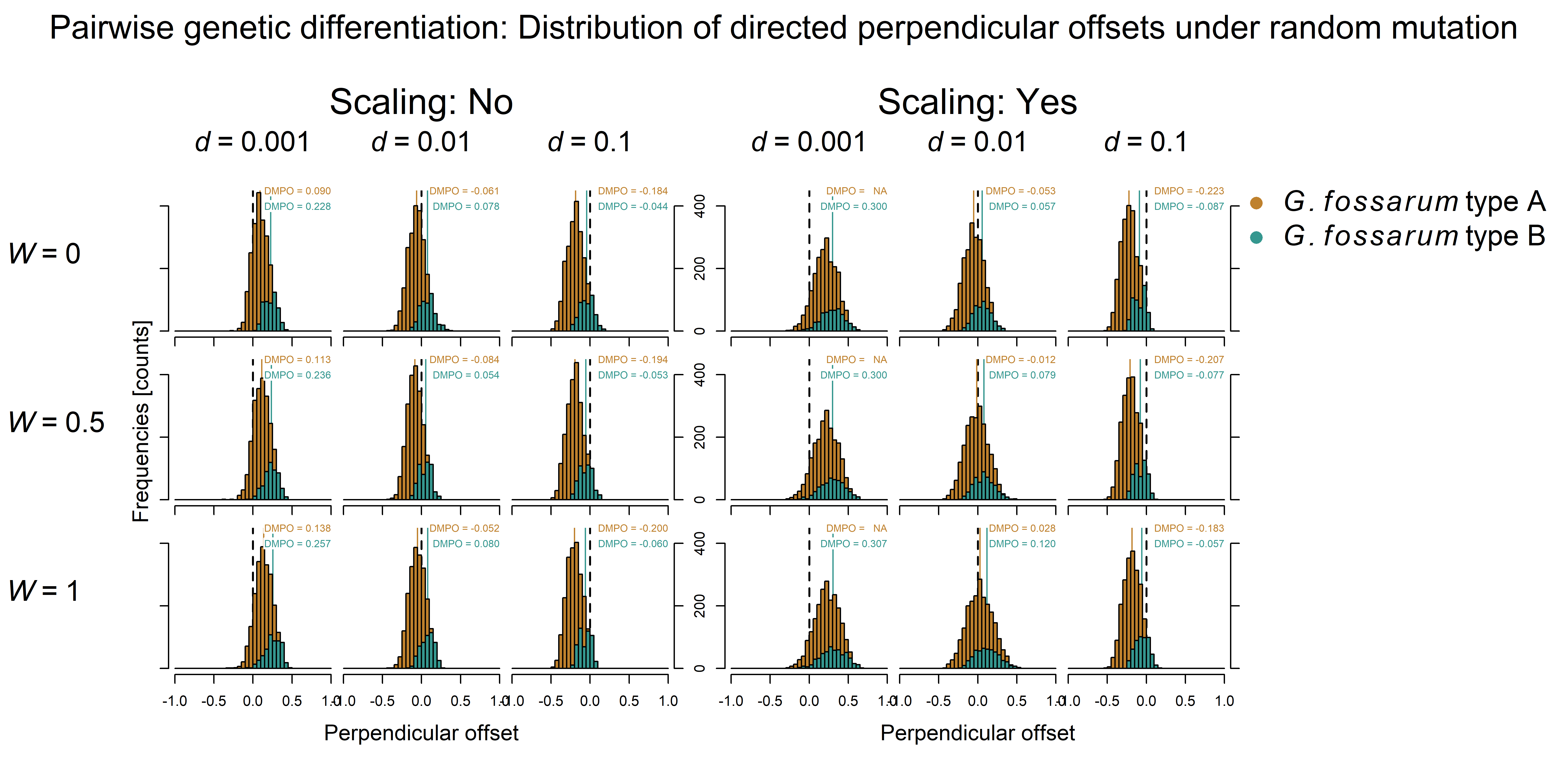


**Figure S50** Histograms and medians of the directed perpendicular offsets (DMPO) between all 18 stochastic simulation scenarios and the empirically assessed pairwise genetic differentiation (Nei F_ST_) values. The directed perpendicular offset does take into account if points are above or below the vertical (1:1) line. The dotted vertical line shows zero offset, corresponding to a perfect fit between simulation and empirical data.

**Table S51** Ranking of the 18 stochastic simulations for their fit to the empirically assessed pairwise genetic differentiation (Nei F_ST_) values according to their sum of perpendicular offsets (SPO). Listed are the value for SPO and the corresponding varying parameter values (dispersal rate *d*, upstream movement probability *W*, and scaling of carrying capacity *K*).

| Rank | Pairwise genetic differentiation | | | | | | | |
| --- | --- | --- | --- | --- | --- | --- | --- | --- |
|  | *G. fossarum* type A | | | | *G. fossarum* type B | | | |
|  | SPO | *d* | *W* | *K* | SPO | *d* | *W* | *K* |
| 1 | 210.4 | 0.01 | 1 | 0 | 39.5 | 0.01 | 0.5 | 0 |
| 2 | 222.3 | 0.01 | 0 | 0 | 42.3 | 0.1 | 1 | 0 |
| 3 | 236.9 | 0.01 | 0.5 | 0 | 42.4 | 0.1 | 0 | 0 |
| 4 | 244.8 | 0.01 | 0 | 1 | 42.8 | 0.1 | 0.5 | 0 |
| 5 | 248.0 | 0.001 | 0 | 0 | 43.4 | 0.1 | 1 | 1 |
| 6 | 264.2 | 0.01 | 0.5 | 1 | 46.2 | 0.1 | 0.5 | 1 |
| 7 | 289.5 | 0.001 | 0.5 | 0 | 49.2 | 0.01 | 1 | 0 |
| 8 | 297.8 | 0.01 | 1 | 1 | 50.2 | 0.1 | 0 | 1 |
| 9 | 332.1 | 0.001 | 1 | 0 | 50.2 | 0.01 | 0 | 0 |
| 10 | 409.2 | 0.1 | 1 | 1 | 52.5 | 0.01 | 0 | 1 |
| 11 | 414.5 | 0.1 | 0 | 0 | 64.3 | 0.01 | 0.5 | 1 |
| 12 | 433.1 | 0.1 | 0.5 | 0 | 80.8 | 0.01 | 1 | 1 |
| 13 | 442.1 | 0.1 | 1 | 0 | 119.5 | 0.001 | 0 | 0 |
| 14 | 454.4 | 0.1 | 0.5 | 1 | 122.9 | 0.001 | 0.5 | 0 |
| 15 | 488.9 | 0.1 | 0 | 1 | 134.1 | 0.001 | 1 | 0 |
| 16 | NA | 0.001 | 0 | 1 | 157.0 | 0.001 | 0 | 1 |
| 17 | NA | 0.001 | 0.5 | 1 | 159.5 | 0.001 | 0.5 | 1 |
| 18 | NA | 0.001 | 1 | 1 | 164.6 | 0.001 | 1 | 1 |

| Rank | Pairwise genetic differentiation | | | | | | | |
| --- | --- | --- | --- | --- | --- | --- | --- | --- |
|  | *G. fossarum* type A | | | | *G. fossarum* type B | | | |
|  | MPO | *d* | *W* | *K* | MPO | *d* | *W* | *K* |
| 1 | 0.084 | 0.01 | 1 | 0 | 0.062 | 0.1 | 1 | 0 |
| 2 | 0.087 | 0.01 | 0 | 0 | 0.069 | 0.1 | 0.5 | 0 |
| 3 | 0.094 | 0.001 | 0 | 0 | 0.070 | 0.1 | 1 | 1 |
| 4 | 0.094 | 0.01 | 0 | 1 | 0.071 | 0.01 | 0.5 | 0 |
| 5 | 0.095 | 0.01 | 0.5 | 0 | 0.072 | 0.1 | 0 | 0 |
| 6 | 0.104 | 0.01 | 0.5 | 1 | 0.078 | 0.1 | 0.5 | 1 |
| 7 | 0.117 | 0.01 | 1 | 1 | 0.083 | 0.01 | 0 | 1 |
| 8 | 0.118 | 0.001 | 0.5 | 0 | 0.085 | 0.01 | 1 | 0 |
| 9 | 0.140 | 0.001 | 1 | 0 | 0.087 | 0.1 | 0 | 1 |
| 10 | 0.183 | 0.1 | 1 | 1 | 0.088 | 0.01 | 0 | 0 |
| 11 | 0.184 | 0.1 | 0 | 0 | 0.100 | 0.01 | 0.5 | 1 |
| 12 | 0.194 | 0.1 | 0.5 | 0 | 0.133 | 0.01 | 1 | 1 |
| 13 | 0.200 | 0.1 | 1 | 0 | 0.228 | 0.001 | 0 | 0 |
| 14 | 0.207 | 0.1 | 0.5 | 1 | 0.236 | 0.001 | 0.5 | 0 |
| 15 | 0.223 | 0.1 | 0 | 1 | 0.257 | 0.001 | 1 | 0 |
| 16 | NA | 0.001 | 0 | 1 | 0.300 | 0.001 | 0 | 1 |
| 17 | NA | 0.001 | 0.5 | 1 | 0.300 | 0.001 | 0.5 | 1 |
| 18 | NA | 0.001 | 1 | 1 | 0.307 | 0.001 | 1 | 1 |

**Table S52** Ranking of the 18 stochastic simulations for their fit to the empirically assessed pairwise genetic differentiation (Nei F_ST_) values according to their median of perpendicular offsets (MPO). Listed are the value for MPO and the corresponding varying parameter values (dispersal rate *d*, upstream movement probability *W*, and scaling of habitat carrying capacity *K*).
